# Orbital Hybridization‐Mediated Decoupling of Electrocatalytic Functions for Paired CO_2_ Electrosynthesis

**DOI:** 10.1002/advs.202522711

**Published:** 2026-01-12

**Authors:** Youjia Wang, Bochen Tian, Yuxin Tian, Wenchao Wang, Xin Ma, Yansheng Liu, Junwei Hou

**Affiliations:** ^1^ State Key Laboratory of Heavy Oil Processing China University of Petroleum Beijing China; ^2^ State Key Laboratory of Heavy Oil Processing China University of Petroleum (Beijing) at Karamay Karamay Xinjiang China

**Keywords:** CO_2_ electroreduction, dual‐scale silver species, morphology‐engineered CeO_2_, orbital hybridization, paired electrosynthesis

## Abstract

The intrinsic trade‐offs between activity, selectivity, and stability pose a fundamental challenge in electrocatalyst design. Here, we address these challenges by constructing a dual‐scale catalytic architecture where traditionally competing functions are decoupled and optimized simultaneously. Our approach is guided by the unique orbital hybridization landscape of CeO_2_ {110} facets, predicted by density functional theory (DFT) to confer a moderate Ag adsorption energy (−4.11 eV), to construct an electronically coupled interface of atomically dispersed Ag_1_ (for CO_2_ activation) and metallic Ag_n_ sub‐nanoclusters (for electron transport). The resulting orbitally hybridized interface boosts oxygen vacancy (O_V_) density by 1.84‐fold and reduces charge‐transfer resistance by 58%. When deployed in a membrane‐free paired electrolyzer, this catalyst enables direct dialkyl carbonate synthesis from CO_2_, achieving 88.53% Faradaic efficiency (FE) for dimethyl carbonate (DMC) at an industrial current density of 52.5 mA·cm^−2^ with 20 h stability, a performance competitive with the state‐of‐the‐art. The versatility of this morphology‐governed orbital hybridization strategy is further demonstrated by the selective production of diethyl carbonate (DEC). This work establishes a rational design principle that controls catalytic synergy through crystallographically defined orbital interactions, offering a promising approach to address persistent trade‐offs in electrocatalysis for CO_2_ valorization.

## Graphical abstract

This study constructs a dual‐scale Ag‐based catalyst on CeO_2_ nanorods via a facet‐guided strategy. This catalyst can induce a cascaded orbital hybridization effect, which synergistically optimizes oxygen vacancy stability, CO desorption kinetics, and charge transfer efficiency. At a current density of 52.5 mA·cm^−2^, the Faradaic efficiency of dimethyl carbonate (DMC) reaches as high as 88.53%.

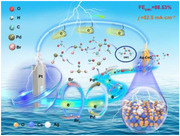

## 1 Introduction

The electrochemical conversion of CO_2_ into value‐added chemicals offers a pivotal route toward sustainable carbon utilization [1, 2, 3], yet its practical deployment is fundamentally limited by persistent activity–selectivity–stability trade‐offs in electrocatalysis [4, 5]. These interdependent constraints are acutely visible in the synthesis of dimethyl carbonate (DMC), a key green chemical and electrolyte [6, 7, 8, 9, 10], where efficient formation requires the precise coordination of CO_2_ activation, CO desorption, and C–O coupling [11, 12], a multistep sequence which has eluded mastery in integrated systems. State‐of‐the‐art electrocatalytic systems consequently fail to exceed 70% Faradaic efficiency (FE) while operating well below the industrial current density benchmark of 50 mA·cm^−2^. A further complication arises from the ubiquitous reliance on the anodic oxygen evolution reaction [13, 14, 15, 16], an energy‐intensive process with high overpotentials exceeding 1.6 V that generates low‐value O_2_, thereby severely compromising the overall energy and economic potential of CO_2_ electrolysis [17, 18, 19].

Established electrocatalyst designs provide only incomplete solutions, inevitably compromising one property for another [20]. Single‐atom catalysts deliver high selectivity and exceptional atom efficiency, but their poor electrical conductivity restricts operation to current densities typically below 12 mA·cm^−2^ [21]. Metallic nanoparticles, in contrast, ensure efficient electron transport [22, 23] yet are plagued by agglomeration and overly strong intermediate binding that poisons active surfaces [24, 25]. Even advanced strategies leveraging metal–support interactions (MSI) often fall short; they typically stabilize only a single type of active site and fail to concurrently meet the divergent requirements of targeted molecular activation and efficient charge transport [26, 27, 28]. Prior studies utilizing support morphology engineering to tune adsorption strengths represent a step forward, but a rational design principle capable of electronically coupling distinct, co‐stabilized active species through a defined physical mechanism remains an unmet challenge in the field [29].

We posit that the key to mitigating electrocatalytic trade‐offs lies in facet‐engineered MSI modulation at the electronic structure level, which transforms the random existence or coexistence of metal species into deliberate functional division of labor, thereby facilitating a synergistic effect superior to simple physical mixtures [30, 31]. Based on density functional theory (DFT) using Ag adsorption energy as a descriptor, we identified a facet‐dependent adsorption behavior of CeO_2_. Calculations reveal excessively strong interaction on {100} facets (−6.79 eV) that drives aggregation, overly weak binding strength on {111} facets (+7.97 eV) that favors atomic dispersion, and moderate adsorption on {110} facets (−4.11 eV) that thermodynamically predisposes co‐stabilization of atomically dispersed Ag_1_ sites and metallic Ag_n_ sub‐nanoclusters. Guided by this prediction, we synthesized {110}‐faceted CeO_2_ nanorods to co‐stabilize atomically dispersed Ag_1_ sites and metallic Ag_n_ sub‐nanoclusters in a single step. X‐ray absorption spectroscopy (XAS) verifies the coexistence of Ag^+^ (Ag–O coordination, 2.09 Å) and Ag^0^ sub‐nanoclusters (Ag–Ag coordination, 2.88 Å). Bader charge analysis quantifies significant interfacial electron transfer (0.30 e^−^ per Ag atom) while integrated crystal orbital Hamiltonian population (ICOHP) calculations provide direct evidence for orbital interactions: electron donation from Ag^0^ 4d to Ce 5d orbitals facilitates oxygen vacancy (O_V_) formation, and optimized coupling between Ag^+^ 4d and O 2p orbitals tailors intermediate adsorption. This synergy yields a 1.84‐fold increase in O_V_ density and 58% reduction in charge‐transfer resistance (R_ct_). Such O_V_ enhancement derives from dual‐scale Ag coupling rather than serving as the key driver. Catalysts with similar O_V_ density but only single‐atom Ag exhibit much faster performance decay over 20 cycles, underscoring the irreplaceable role of dual‐scale Ag in sustaining stability and activity. The intrinsic merit of this design is demonstrated in fundamental CO_2_‐to‐CO conversion (92.51% FE_CO_, at 39.3 mA·cm^−2^). Its greater value is realized in cascade electrosynthesis, where it addresses a key challenge by simultaneously and efficiently supplying the two essential precursors, CO and ^*^CH_3_O^−^, whose availability dictates the DMC yield. Gold foil control experiments corroborate this point. Despite moderate FE_CO_ (82.41%), the gold foil lacks dual‐scale Ag coupling and functional division, leading to only 61.09% FE_DMC_. Physical mixtures of single‐atom and cluster catalysts also fail to replicate this performance (78.06% FE_DMC_), confirming synergistic electronic coupling is indispensable. CO temperature‐programmed desorption (102.8°C), and DFT calculations (near‐thermoneutral ^*^CH_3_O^−^ formation, Gibbs free energy barrier (ΔG) = −0.40 eV) further verify balanced precursor supply that avoids surface poisoning or accumulation. Integrating this cathode into a membrane‐free paired electrolyzer enables direct DMC synthesis with 88.53% FE_DMC_ at an industrial current density of 52.5 mA·cm^−2^ and stable 20 h operation. The design principle's adaptability is confirmed by successful extension to diethyl carbonate (DEC) synthesis. Overall, these findings establish a rational design framework for complex electrocatalysis, demonstrating the potential of facet‐specific orbital hybridization in mitigating persistent property trade‐offs in integrated electrochemical systems.

## 2 Results and Discussion

### 2.1 Facet‐Dependent MSI Engineering Enables Predictive Construction of Dual‐Scale Ag Active Centers

**FIGURE 1:**
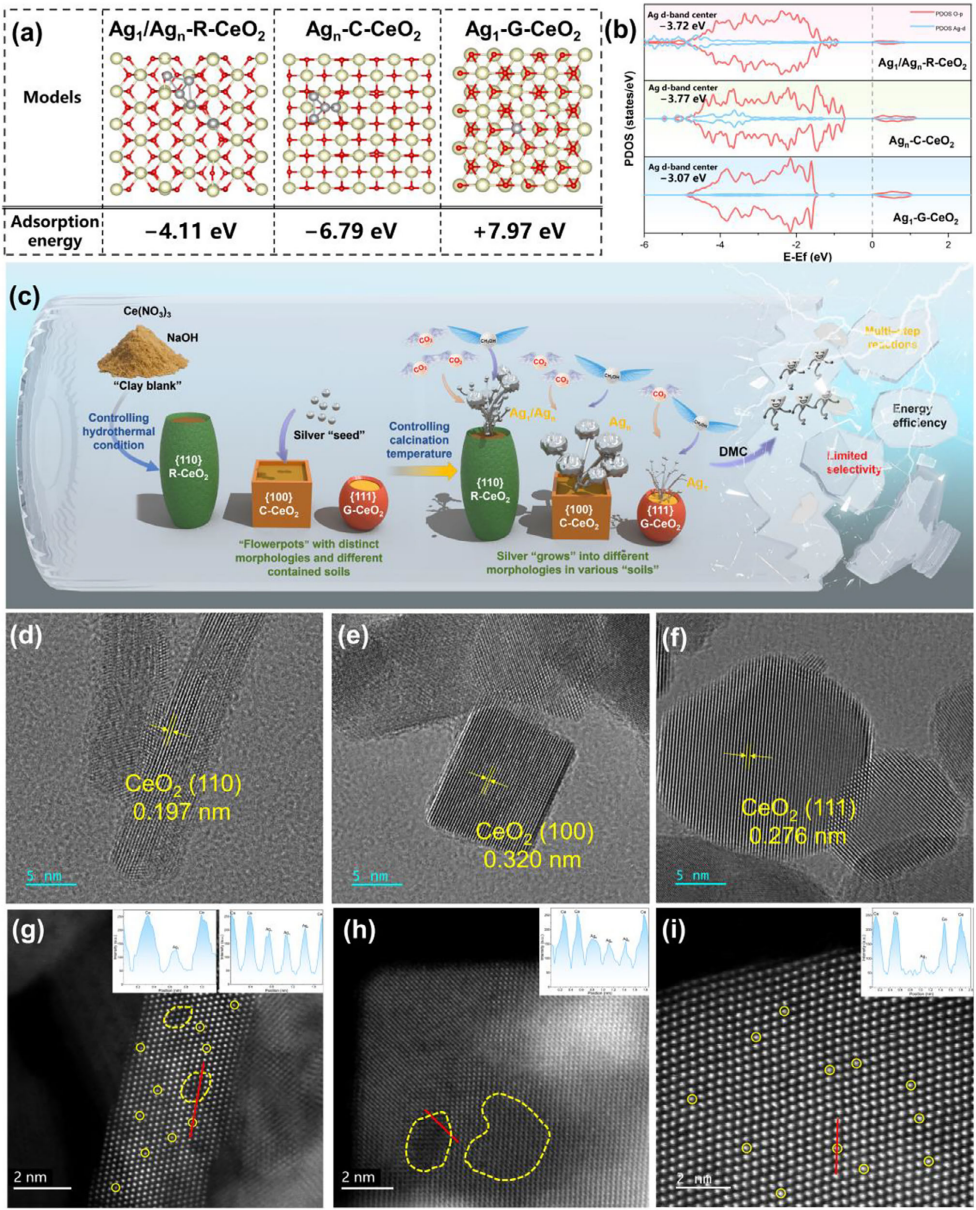
Morphology‐Engineered Ag‐CeO_2_ Catalysts: (a) DFT Calculations of Surface Energy for Different Morphologies, (b) DOS Calculations of d‐band Centers, (c) “Flowerpot‐Soil‐Seedling” Model: Facile Synthesis of Silver‐Based Catalysts Driven by the Crystal‐Facet Effect of Ceria (d–f) TEM Images Showing Facet‐Specific Structures of Ag_1_/Ag_n_‐R‐CeO_2_ ({110} Facets), Ag_n_‐C‐CeO_2_ ({100} Facets), and Ag_1_‐G‐CeO_2_ ({111} Facets), and (g–i) HAADF‐STEM Images of Ag_1_/Ag_n_‐R‐CeO_2_ ({110} Facets), Ag_n_‐C‐CeO_2_ ({100} Facets), and Ag_1_‐G‐CeO_2_ ({111} Facets), Along with Their Corresponding Line‐Scanning Intensity Profiles Demonstrating Ag Species Distribution (Single Atoms and Clusters).

We aimed to establish a rational design principle for Ag‐CeO_2_ catalysts by moving beyond the common occurrence of random metal size distributions. Our strategy was to theoretically identify a specific CeO_2_ facet capable of thermodynamically stabilizing both atomically dispersed Ag (for precise activation) and metallic clusters (for charge transport), and then experimentally realize this targeted dual‐scale architecture. We first employed DFT calculations to decipher the facet‐dependent MSI, constructing models of the three dominant CeO_2_ surface terminations ({111}, {100}, and {110}, (Figure S4) and evaluated the stability of various Ag species on them. Our DFT analysis (Figure 1a) confirms this premise, revealing a clear hierarchy in the thermodynamic stability of Ag species. On the {111} facet, substitutional doping is highly endothermic (+7.97 eV), thermodynamically confining Ag to isolated single atoms. Conversely, the {100} facet exhibits an excessively strong adsorptive MSI (−6.79 eV), which promotes Ag migration and coalescence into large clusters. Crucially, the {110} facet achieves an optimal balance with a moderate adsorption energy (−4.11 eV), theoretically enabling the concurrent stabilization of both substitutional Ag single atoms and adsorbed sub‐nanometric Ag_n_ clusters. Density of states (DOS) calculations clarify the electronic basis of this optimal interaction (Figure 1b). The Ag d‐band center (ε_d_) upshifts to −3.72 eV on {110}, intermediate between the values for {100} (−3.77 eV) and {111} (−3.07 eV). This intermediate position balances adsorbate binding strength, avoiding the weak interaction on {111} and the overly strong interaction on {100}. The tailored electronic state on the {110} facet thus primes the interface for the synergistic integration of the dual‐scale Ag ensemble.

Guided by these DFT predictions of both energetic stability and electronic structure, we developed a synthesis strategy inspired by a “Clay‐Vase‐Seed” concept to fabricate distinct CeO_2_ nanostructures with targeted facet exposures. In this concept, the precursor reagents serve as the “Clay” (Cerium nitrate and NaOH), which, through hydrothermal treatment and calcination, is crystallized and hardened into defined CeO_2_ “Vases” with specific facet geometries ({100}, {110}, or {111}), as shown in Figure 1c (details in the experimental section). Precise control over the “clay” composition (NaOH: 3.6–9 g) and processing conditions (hydrothermal: 90°C–180°C; calcination: 200°C–600°C) allowed for the reproducible fabrication of three pure‐phase architectures, cubic (C‐), rod‐like (R‐), and granular (G‐CeO_2_). Inductively coupled plasma optical emission spectroscopy (ICP‐OES) confirmed that the actual Ag loading in Ag_1_/Ag_n_‐R‐CeO_2_ was 3.04 wt.% (Table S2), closely matching the designed value of 3.0 wt.%. With Ag loading thus fixed, each CeO_2_ “vase” was then used to template a unique Ag morphology through facet‐specific MSI, providing an ideal platform to experimentally validate the theoretical predictions.

Having established the electronic‐structure basis of MSI and its validation through controlled synthesis, we next interrogated how these interactions shape long‐range structural order to stabilize the dual‐scale Ag architecture. X‐Ray Diffraction (XRD) analysis (Figure S5) confirms the preservation of the fluorite CeO_2_ framework (PDF#43‐1002) in all catalysts. The diffraction intensities of metallic Ag (38.1°, PDF#89‐3722) [32], however, starkly contrast the facet‐dependent dispersion. Intense peaks for Ag_n_‐C‐CeO_2_ ({100}) signify severe aggregation, while moderate intensity for Ag_1_/Ag_n_‐R‐CeO_2_ ({110}) indicates limited clustering. Nearly absent peaks for Ag_1_‐G‐CeO_2_ ({111}) corroborate its superior dispersion. We identify the {110} facet's unique lattice strain as a pivotal factor contributing to this stabilization. Williamson–Hall analysis (Table S3) quantifies that the anisotropic growth of R‐CeO_2_ introduces an inherent tensile strain (ε = 0.3887%). Upon Ag incorporation, this strain is selectively relaxed to ε = 0.2090% via a dual‐path mechanism. Atomically dispersed Ag_1_ mitigates strain through charge‐balanced Ag^+^─O─Ce^3+^ bonds, while sub‐nanometric Ag_n_ clusters relieve interfacial stress. Consequently, the {110} facet demonstrates a unique ability to maintain cooperative strain relaxation, while G‐CeO_2_ and C‐CeO_2_ show either negligible or disruptive strain changes, failing to accommodate both silver species. Thus, beyond electronic MSI, the {110} facet's ability to leverage lattice strain provides a critical crystallographic foundation for the synergistic Ag_1_/Ag_n_ ensemble.

Having verified the bulk crystal and strain properties, we directly visualized the resulting morphologies and the facet‐dependent distribution of Ag species. Scanning electron microscopy (SEM) confirms uniform morphological distribution across the entire batch (Figures S6–S8), while transmission electron microscopy (TEM) reveals clear facet orientation (Figure 1d–f; Figures S6–S8). The targeted R‐CeO_2_ forms highly ordered 1D nanostructures (40–90 nm in length, 6–7 nm in diameter, Figure S6) with dominant {110} facets, as indicated by a lattice spacing of 0.197 nm; C‐CeO_2_ forms regular cubes (20–60 nm edge length, Figure S7) with {100} facets (0.320 nm); and G‐CeO_2_ appears as irregular particles (with sizes ranging from 10 to 40 nm, Figure S8) exposing {111} facets (0.276 nm) [33, 34]. The facet‐dependent Ag distribution was investigated at the atomic scale. Atomic‐resolution High‐Angle Annular Dark‐Field Scanning Transmission Electron Microscopy (HAADF‐STEM) imaging (2 nm), complemented by intensity profile analysis and EDS mapping (Figure S9), confirms our DFT predictions. For Ag_1_‐G‐CeO_2_ ({111} facets), only isolated bright spots are observed (Figure 1i). Their corresponding intensity profiles show sharp, atomic‐scale peaks, and EDS confirms a highly dispersed Ag distribution, consistent with exclusively atomically dispersed Ag and the predicted weak MSI. In contrast, Ag_n_‐C‐CeO_2_ ({100} facets) exhibits large, aggregated clusters (≥ 2 nm) (Figure 1h). The intensity profiles across these features show broad plateaus of elevated intensity, and EDS maps show highly concentrated Ag signals, confirming aggregation driven by excessively strong MSI. Critically, Ag_1_/Ag_n_‐R‐CeO_2_ ({110} facets) uniquely shows the coexistence of atomic‐scale spots and uniformly dispersed 1–2 nm sub‐nanometric clusters (Figure 1g). Intensity profiles unambiguously distinguish sharp peaks (single atoms) from broader regions of moderate intensity (clusters). EDS mapping verifies both a dispersed background and localized cluster signals. This direct visualization of dual‐scale Ag species validates the balanced MSI unique to the {110} facet, forming the desired synergistic active sites.

**FIGURE 2:**
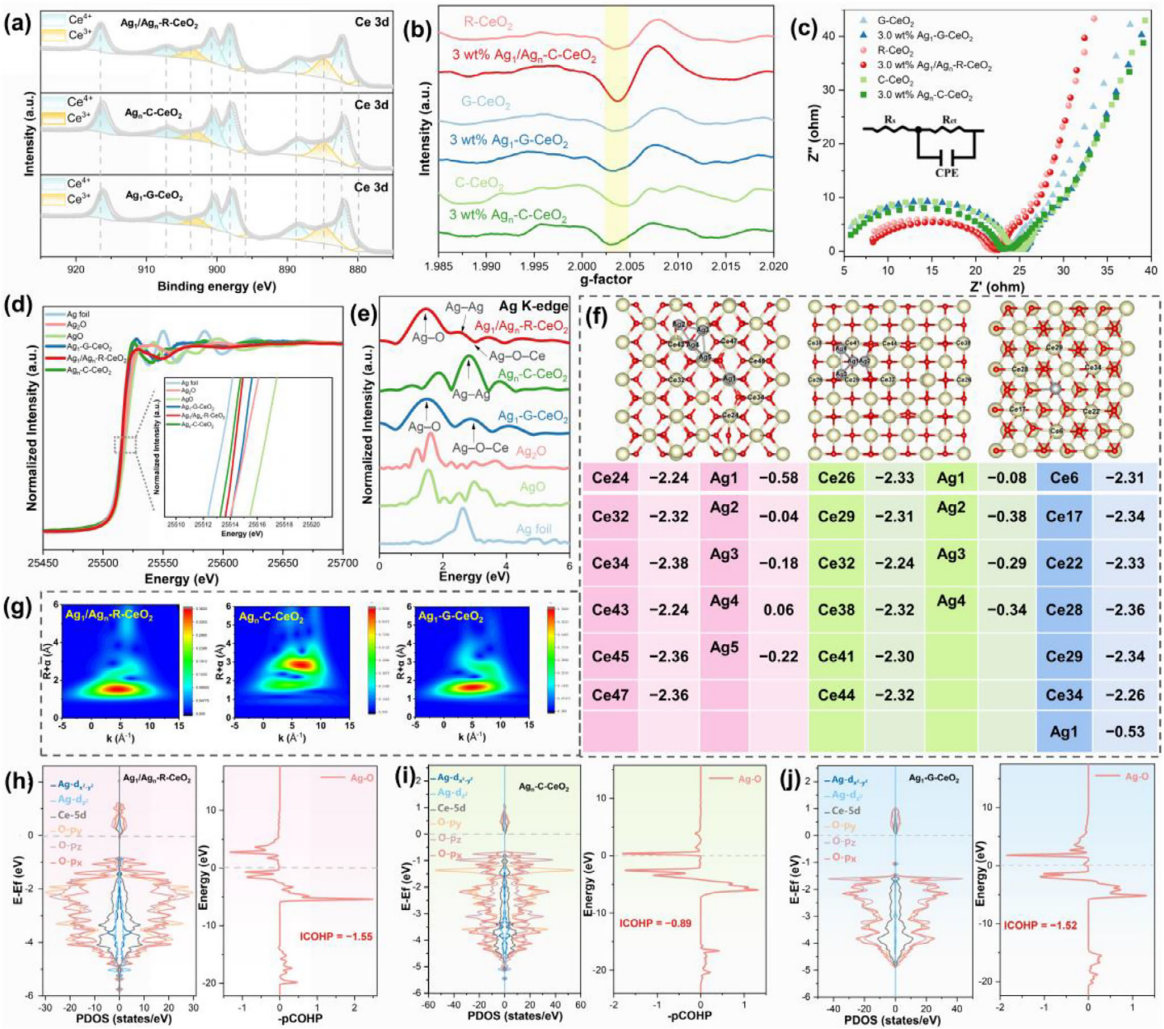
Spectroscopic and Theoretical Insights into the Dual‐Scale Ag‐CeO_2_ Catalyst: (a) XPS Ce 3d Spectra for Quantifying Ce^3+^ Fractions, (b) EPR Spectra Illustrating O_V_ Concentrations, (c) EIS Nyquist Plots Reflecting R_ct_, (d) Ag K‐edge XANES Spectra, (e) Ag K‐edge EXAFS Spectra Depicting Ag Local Coordination Environments, (f) Bader Charge Analysis of Electron Redistribution, (g) WT‐EXAFS spectra of Ag species, and (h–j) PDOS of Ag, Ce, and O Orbitals, Coupled with Corresponding ‐pCOHP Curves and ICOHP Values, for Ag_1_/Ag_n_‐R‐CeO_2_, Ag_n_‐C‐CeO_2_, and Ag_1_‐G‐CeO_2_, Illustrating Orbital Hybridization and Ag─O Bonding Strength.

As predicted by our initial DFT calculations, the intrinsic nature and strength of the MSI directly control the facet‐dependent dispersion of Ag species, which ranges from exclusively isolated single atoms to coexisting single atoms and sub‐nanometric clusters and further to large aggregates. Complementary electronic structure analyses provide further corroboration of this MSI hierarchy. X‐ray Photoelectron Spectroscopy (XPS) (Figure S10a) of Ag_1_/Ag_n_‐R‐CeO_2_, Ag_n_‐C‐CeO_2_, and Ag_1_‐G‐CeO_2_ confirms the coexistence of Ce, O, and Ag in all samples, with facet‐dependent variations in Ag signal intensity reflecting differences in Ag loading and dispersion. A systematic increase in Ag 3d_5/2_ XPS binding energies (Figure S11a) from 368.01 eV on {111} facets to 368.11 eV on {100} and further to 368.28 eV on {110} is observed. The predicted electronic perturbation finds its experimental signature in a 0.27 eV upshift of the Ag 3d_5/2_ XPS binding energy on the {110} facet, corroborating the computational evidence for enhanced interfacial electron donation mediated by facet‐specific orbital hybridization. Quantified by Bader charge analysis (Figure 2f), this electron donation is more pronounced on the {110} facet than on the others, a distinct charge distribution pattern that lays the experimental groundwork for the subsequent electronic‐structure analysis. Collectively, these results demonstrate that our DFT‐guided understanding of MSI directly dictates Ag dispersion. Specifically, weak, substitution‐limited MSI on {111} stabilizes only isolated Ag_1_; strong, adsorption‐driven MSI on {100} induces large cluster aggregation; and only the balanced, dual‐mode MSI on {110} enables the synergistic Ag_1_/Ag_n_ coexistence, laying the critical structural foundation for efficient electroreduction.

Beyond the atomic‐scale interplay of strain and MSI, we hypothesized that the facet‐governed morphology of CeO_2_ would also architecturally define the pore structure, which in turn dictates the spatial distribution and cooperative function of the dual‐scale Ag species. To test this, we performed CO_2_ physisorption at 195 K (Figures S12–S14). The analysis reveals that the rod‐like morphology of Ag_1_/Ag_n_‐R‐CeO_2_, dominated by {110} facets, forms a hierarchically porous architecture with a high Brunauer–Emmett–Teller (BET) surface area (106 m^2^·g^−1^), a substantial micropore area (48.9 m^2^·g^−1^), and a large total pore volume (0.0998 cm^3^·g^−1^). These structural advantages yield property metrics substantially exceeding those of Ag_n_‐C‐CeO_2_ (51.7, 18.1 m^2^·g^−1^, 0.0519 cm^3^·g^−1^) and Ag_1_‐G‐CeO_2_ (28.0, 11.6 m^2^·g^−1^, 0.0282 cm^3^·g^−1^). The type IV isotherm with a distinct H2 hysteresis loop unequivocally confirms the presence of mesopores (∼3.76 nm, ideally sized to accommodate the 1–2 nm Ag_n_ clusters) originating from inter‐rod voids. Beyond merely influencing site organization, this hierarchical porosity is essential for structuring the catalytic sites effectively. The micropores structurally stabilize atomically dispersed Ag^+^–O–Ce^3+^ centers (consistent with high CO_2_ adsorption), while the inherent mesopores provide the necessary confinement to accommodate the 1–2 nm Ag_n_ clusters directly visualized by HAADF‐STEM. Enabling a direct functional decoupling, the atomic sites drive activation while the hosted clusters facilitate electron transfer. Therefore, the {110} facet serves as a unique structural platform that simultaneously engineers the electronic structure (via orbital‐hybridization‐mediated MSI) and the mass‐transport environment (via hierarchical porosity), collectively establishing a multifunctional catalytic architecture that successfully decouples and concurrently optimizes the traditionally antagonistic properties of molecular activation and charge transport.

### 2.2 Orbital Hybridization‐Mediated Concurrent Promotion of O_V_ Formation and Charge Conductivity on CeO_2_ {110}

Building on the established morphological advantages of {110}‐faceted R‐CeO_2_, we now elucidate its unique electronic interplay with silver species. The exceptional electrocatalytic performance of Ag_1_/Ag_n_‐R‐CeO_2_ originates from a distinct electronic interplay between silver species and the {110} facets of CeO_2_ nanorods. Driven by the facet‐specific dual‐mode MSI, a synergistic ensemble comprising atomically dispersed Ag_1_ and metallic Ag_n_ clusters is stabilized through deliberate design, which enables the decoupling and concurrent optimization of two traditionally antagonistic properties, O_V_ density and charge conductivity. We ascribe this electronic synergy to a three‐stage cascaded orbital hybridization mechanism involving Ag, Ce, and O within the CeO_2_ lattice, consistently supported by DFT calculations and comprehensive spectroscopic analysis (Figure 2).

O_V_ formation is intimately tied to electronic interactions triggered by Ag species. X‐ray photoelectron spectroscopy (XPS) measurements (Figure 2a) indicate a Ce^3+^ fraction of 16.83% in Ag_1_/Ag_n_‐R‐CeO_2_, higher than those of Ag_n_‐C‐CeO_2_ (14.85%) and Ag_1_‐G‐CeO_2_ (12.67%). These stable Ag^+^–O–Ce^3+^ configurations inhibit the oxidation of Ce^3+^ sites and are directly correlated with increased O_V_ density [35, 36]. Consistent with this trend, electron paramagnetic resonance (EPR) spectroscopy of Ce^3+^ species (Figure S15) displays the strongest signal intensity (g = 1.965) for Ag_1_/Ag_n_‐R‐CeO_2_, confirming its highest Ce^3+^ content. EPR analysis focused on O_V_ defects (Figure 2b) further reveals a 1.84‐fold increase in O_V_ concentration relative to pristine R‐CeO_2_, substantially surpassing the modest enhancements in Ag_n_‐C‐CeO_2_ (1.05‐fold) andAg_1_‐G‐CeO_2_ (1.13‐fold). Additional support for facet‐selective O_V_ enrichment comes from XPS O 1s spectra (Figure S16a), where Ag_1_/Ag_n_‐R‐CeO_2_ shows the most pronounced O_V_ signal. All XAS fitting curves, including R‐space and K‐space data, are provided in Figure S17 to fulfill characterization requirements.

The initial stage of interfacial interaction involves Ag^0^‐to‐Ce electron transfer, weakening Ce─O bonds for O_V_ formation [37, 38, 39]. Leveraging the optimal adsorption energy of −4.11 eV on {110} facets (Figure 1a), metallic Ag^0^ sub‐nanoclusters anchor firmly. Their 4dx^2^‐y^2^ orbitals engage in effective electronic coupling with Ce 5d and O 2p orbitals near the Fermi level, as shown by partial density of states (PDOS) analysis (Figure 2h). This directional interaction promotes efficient electron transfer from Ag^0^ to Ce^4+^, quantified by Bader charge analysis as a gain of 2.24–2.38 e^−^ per Ce atom (Figure 2f). The subsequent reduction of Ce^4+^ to Ce^3+^ breaks the ideal closed‐shell configuration of Ce^4+^ (4f^0^), generating Ce 5d orbitals accessible for electronic interaction with Ag. Quantitative integrated crystal orbital Hamiltonian population (ICOHP) calculations verify that this electron transfer directly weakens neighboring Ce─O bonds [40]. For the Ag‐doped {110} facet, the Ce─O bond exhibits an ICOHP value of −3.47 eV, less negative than that in the Ag‐doped {100} facet model (−4.31 eV) and the pristine rod‐like CeO_2_ (−4.91 eV). This quantitative evidence confirms that charge transfer thermodynamically destabilizes the Ce–O lattice, establishing the electronic basis for Ov formation and stabilization. In contrast, the weak Ag adsorption energy on {111} facets in Ag_1_‐G‐CeO_2_ limits such electronic coupling, leading to inadequate O_V_ generation.

The second stage of interfacial modulation tailors the active interface by optimizing Ag^+^–O interactions. Electron transfer from Ag^0^ raises the energy of 4dz^2^ orbitals in substitutional Ag^+^ ions, promoting energy matching with O 2p orbitals in the CeO_2_ {110} lattice and forming orbital coupling with σ‐type characteristics. Structural evidence for Ag^+^–O σ–hybridization comes from extended X‐ray absorption fine structure (EXAFS) analysis, which identifies a short‐range Ag–O coordination number of 3.2 at a bond length of 2.09 Å (Figure 2e). The corresponding fitting curves are provided in the Supporting Information. ICOHP analysis quantifies the balanced Ag─O bond strength in Ag_1_/Ag_n_‐R‐CeO_2_ as −1.55 eV, stronger than that in Ag_n_‐C‐CeO_2_ (−0.89 eV) and comparable to the Ag‐doped {111} facet (Ag_1_‐G‐CeO_2_, −1.52 eV). As a result, electron density becomes localized around O_V_ sites, which suppresses their reoxidation and reinforces the stability of the O_V_‐enriched active interface. The functional consequence of this electronic modulation is observed in CO‐temperature programmed desorption (CO‐TPD, Figure 4a), where Ag_1_/Ag_n_‐R‐CeO_2_ shows a distinct desorption peak at 102.8°C. Resulting from the electron depletion in O 2p orbitals induced by Ag^+^–O σ‐hybridization, the distinct desorption peak reflects a moderate Ag–CO binding energy that optimally balances reactant activation with desorption. A control catalyst with similar O_V_ concentration but only a single Ag species (Ag_1_‐G‐CeO_2_) confirms that dual‐scale Ag enhances the intrinsic activity of O_V_ sites via optimized Ag–CO binding energy, rather than merely increasing O_V_ concentration. On {100} facets, however, excessively strong adsorption drives Ag aggregation in Ag_n_‐C‐CeO_2_, disrupting optimal interfacial coupling and impairing O_V_ stability [41].

The third stage focuses on charge transport enhancement via metallic Ag° clusters [42, 43]. Electrochemical impedance spectroscopy (EIS) Nyquist plots (Figure 2c) demonstrate a remarkably low charge transfer resistance (R_ct_) of 12.68 Ω for Ag_1_/Ag_n_‐R‐CeO_2_ (Table S4), which is 3% below that of Ag_n_‐C‐CeO_2_ (13.07 Ω), 19% below Ag_1_‐G‐CeO_2_ (15.63 Ω), and 29%–45% lower than pure CeO_2_ supports. Under EXAFS analysis, the presence of a conductive network formed via d–d orbital coupling between Ag^0^ sub‐nanoclusters is directly confirmed by Ag–Ag coordination spectra showing a coordination number of 1.4 and bond length of 2.88 Å (Figure 2e; Table S5) [44]. The relatively weak Ag–Ag signal intensity in Ag_1_/Ag_n_‐R‐CeO_2_ compared to Ag_n_‐C‐CeO_2_ stems from smaller cluster sizes in the rod‐like structure, as fewer scattering pairs in small clusters yield weaker EXAFS signals. The shorter Ag─Ag bond length aligns with structural contraction commonly observed in ultra‐small clusters [45, 46, 47]. HAADF‐STEM imaging (Figure 1g) further confirms the distribution of dual‐scale Ag species on rod‐like CeO_2_. Wavelet transform (WT) analysis of the Ag K‐edge (Figure 2g) provides further structural resolution. The corresponding contour plot for Ag_1_/Ag_n_‐R‐CeO_2_ exhibits a pronounced Ag–O signal together with a faint tail extending into the high‐k region, representing a characteristic signature of spatially confined Ag–Ag interactions within sub‐nanometric clusters. Such a spectral profile stands in clear contrast to that of Ag_1_‐G‐CeO_2_, where only a singular Ag–O coordination feature is observed, indicative of absent extended conductive pathways. It also differs markedly from Ag_n_‐C‐CeO_2_, in which intense Ag–Ag dominated signals reflect nanoparticle aggregation that leads to increased electron scattering. HAADF‐STEM imaging (Figure 1g) reveals a spatial proximity of ∼1 nm between Ag_1_ and Ag_n_ species, enabling weak inter‐species orbital coupling and collectively forming an electron relay that shuttles electrons from the Ag^0^ network to the Ag^+^–O active sites.

X‐ray absorption spectroscopy (XAS) and EXAFS analyses yield unambiguous evidence for electronic coupling beyond the physical coexistence of Ag_1_ and Ag_n_ species. As shown in Figure 2d, the Ag K‐edge absorption edge position (25 512 eV) in Ag_1_/Ag_n_‐R‐CeO_2_ indicates mixed valence states, intermediate between metallic Ag^0^ (25 508 eV) [48, 49] and Ag^+^ in Ag_2_O (25 515 eV) [50, 51]. EXAFS fitting quantitatively verifies this dual configuration, featuring both short‐range Ag–O coordination and mid‐range Ag–Ag interactions. Complementary Ce K‐edge XANES spectra (Figure S18) demonstrate that Ag incorporation perturbs Ce electronic states, inducing lattice expansion in Ag_1_/Ag_n_‐R‐CeO_2_. EXAFS measurements confirm this structural response, showing a lengthened Ce─O bond of 2.28 Å relative to 2.31 Å in bulk CeO_2_ (Figure S19). WT‐EXAFS of the Ce K‐edge (Figures S20–S22) provides further insight from the support's viewpoint, revealing a discernible Ce–Ag feature in Ag_1_/Ag_n_‐R‐CeO_2_ that reflects weak electronic interactions between Ce and Ag. This observation aligns with our ICOHP calculations, which quantify extremely weak Ce─Ag bonding (ICOHP = −0.18 eV) that is insufficient for stable chemical bonding but enables electron transfer. The corresponding signal in Ag_1_‐G‐CeO_2_ is consistent with a highly localized substitutional environment, whereas Ag_n_‐C‐CeO_2_ exhibits widespread perturbation of Ce coordination caused by interfacial Ag clusters.

The interfacial charge redistribution and bond strength variations are directly quantified by Bader charge analysis and ICOHP calculations (Figure 2h–j; Tables S6 and S7). In the optimal Ag_1_/Ag_n_‐R‐CeO_2_ system, electron transfer leads to a charge gain of 2.24–2.38 e^−^ per Ce atom, significantly exceeding values measured in Ag_1_‐G‐CeO_2_ (1.82–1.95 e^−^) and Ag_n_‐C‐CeO_2_ (1.67–1.81 e^−^). Meanwhile, the Ag species retain near‐neutral Bader charges between −0.22 and +0.06 e^−^, constituting a metastable, relay‐like state characteristic of the synergistic ensemble. Referenced against the 4.20% excess Ce^3+^ detected by XPS, this extent of charge transfer corresponds to an extra 0.13–0.14 e^−^ localized per newly formed Ce^3+^ site, which quantitatively explains the 0.03 Å elongation of the Ce─O bond observed in EXAFS. ICOHP calculations further confirm that Ag incorporation on the {110} facet strengthens the Ag–O interaction while simultaneously weakening the neighboring Ce─O bonds. The weakened Ce─O bonding provides a quantitative electronic‐structure basis for the facilitated formation of O_Vs_. PDOS analysis (Figure 2h) further illuminates the atomic‐scale basis of this distinctive charge distribution. The superior charge transfer on {110} facets results from directional orbital coupling between Ag^+^ 4d and O 2p, and between Ag^0^ 4d and Ce 5d, near the Fermi level. These interactions exhibit distinct symmetry characteristics that collectively propel an efficient electron relay from Ag into the CeO_2_ lattice. As it propagates through the lattice, this primary Ag–O interaction perturbs the Ce–O network and generates a cascaded effect that optimizes intermediate adsorption. The collective evidence establishes that the complete three‐stage interfacial modulation occurs exclusively on the {110} facet, a singular synergy that underpins the exceptional electrocatalytic performance elaborated in subsequent sections.

### 2.3 A Synergistic Dual‐Scale Silver Catalyst for Versatile CO_2_ Electrosynthesis

**FIGURE 3:**
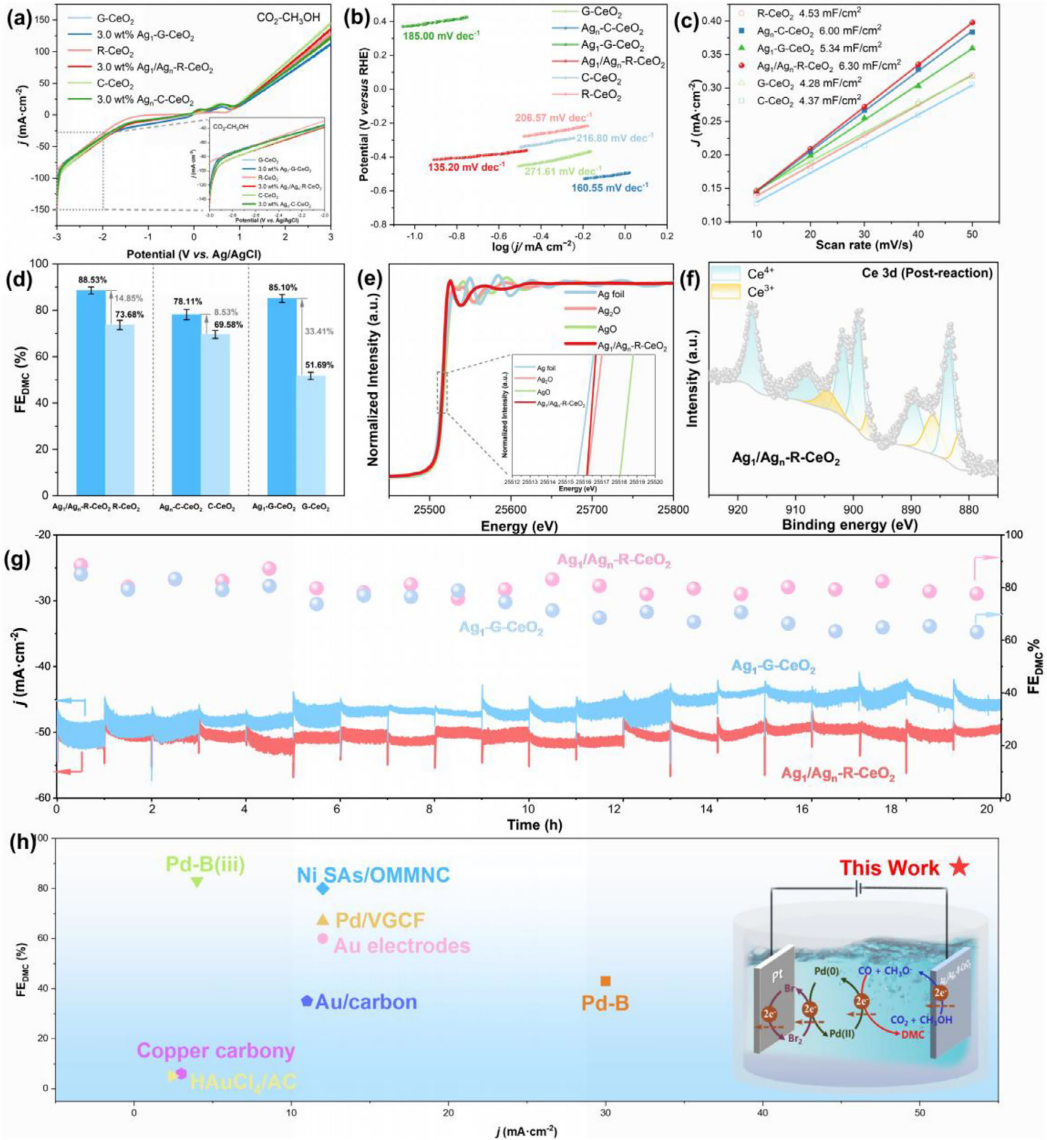
Dual‐scale Ag synergy enables high‐selectivity, high‐current DMC electrosynthesis with structural robustness: (a) LSV of Ag‐modified CeO_2_ cathodes in CO_2_‐saturated CH_3_OH/NaBr electrolyte, (b) Tafel plots for CO_2_‐to‐^*^COOH activation step, (c) Electrochemical Cdl measurements, (d) FE_DMC_ at −2.2 V vs Ag/AgCl, demonstrating morphology‐dependent selectivity, (e) Ag K‐edge EXAFS Spectra of Ag_1_/Ag_n_‐R‐CeO_2_ Catalysts Post‐Reaction (After 20 Operational Cycles), Depicting the Post‐Reaction Ag Local Coordination Environments, (f) Ce 3d XPS spectra of Ag_1_/Ag_n_‐R‐CeO_2_ after catalysis, indicating increased Ce^3+^ content, (g) 20 h durability test of Ag_1_/Agn‐R‐CeO_2_ and Ag_1_‐G‐CeO_2_ at ∼50 mA·cm^−2^, showing the stable current density and FE_DMC_ of Ag_1_/Ag_n_‐R‐CeO_2_ in comparison with the performance of Ag_1_‐G‐CeO_2_, (h) Performance benchmarking of our system against state‐of‐the‐art CO_2_‐to‐DMC and CO‐to‐DMC routes from literature, Data for FE and product concentration represent the mean values of three independent measurements. Error bars indicate the standard deviation.

Having established the interfacial electronic coupling of Ag_1_/Ag_n_‐R‐CeO_2_, we integrate it into a membrane‐free paired electrolyzer to directly synthesize dialkyl carbonates from CO_2_. By integrating the Ag_1_/Ag_n_‐R‐CeO_2_ cathode with a homogeneous Pd/Br^−^ mediator, this configuration strategically replaces the energy‐intensive oxygen evolution reaction with value‐added bromide oxidation (Figure 3h). Three control experiments conducted under identical electrolyte and CO_2_ flow conditions confirm the indispensability of this electrochemical‐thermochemical synergy for DMC formation (Gaseous CO was quantified by online gas chromatography, and liquid products were analyzed by GC‐FID with an internal standard, for details, see the Methods section). A membrane‐separated H‐type electrolyzer produced only CO with a FE_CO_ exceeding 92% and no detectable DMC (Figure S23). An electrolyte without methanol yielded no DMC, and only CO. A system without Pd/C failed to form DMC, verifying the critical role of Pd/C‐mediated thermochemical C–O coupling and confirming that both methanol and Pd/C are indispensable for DMC synthesis [52, 53] (Figure S24).

Before evaluating the complex DMC synthesis, we probed the intrinsic activity of the catalysts for the fundamental CO_2_ reduction reaction in a standard H‐type cell. Linear sweep voltammetry (LSV, Figure 3a) under a CO_2_ atmosphere demonstrates that the Ag_1_/Ag_n_‐R‐CeO_2_ cathode delivers a current density of 48.7 mA·cm^−2^ at −2.2 V (vs. Ag/AgCl), exceeding the 40 mA·cm^−2^ industrial threshold and showing a modest improvement over Ag_1_‐G‐CeO_2_ (47.3 mA·cm^−2^) and Ag_n_‐C‐CeO_2_ (47.1 mA·cm^−2^). Control LSV measurements in an Ar‐saturated electrolyte (Figure S25) were conducted to deconvolute the current contributions. Strikingly, although the result reveals that the rod‐like morphology of the pure R‐CeO_2_ support itself contributes to a substantial hydrogen evolution reaction (HER) background (26.5 mA·cm^−2^), the introduction of the dual‐scale silver centers on Ag_1_/Ag_n_‐R‐CeO_2_ not only supersedes this HER background but also directs the majority of the FE toward CO_2_ reduction. More importantly, under potentiostatic conditions, Ag_1_/Ag_n_‐R‐CeO_2_ achieved an FE_CO_ of 92.51%, while Ag_1_‐G‐CeO_2_ and Ag_n_‐C‐CeO_2_ achieved 84.77% and 81.96%, respectively (Figure S23). Although the absolute FE_CO_ difference may appear modest, achieving and maintaining such high selectivity at near‐industrial current densities highlights the advantage of the Ag_1_/Ag_n_‐R‐CeO_2_ structure in efficiently and stably providing the crucial carbon feedstock (CO) for downstream C–O coupling. These findings confirm the dual‐scale Ag ensemble's superior intrinsic activity and selectivity.

Kinetic analysis via Tafel plots (Figure 3b) further elucidates how the electronic synergy arising from orbital hybridization accelerates the rate‐determining ^*^COOH formation step. Ag_1_/Ag_n_‐R‐CeO_2_ exhibits the lowest Tafel slope (135.2 mV·dec^−1^) compared to Ag_1_‐G‐CeO_2_ (185.0 mV·dec^−1^) and Ag_n_‐C‐CeO_2_ (160.6 mV·dec^−1^), demonstrating superior kinetics as a direct kinetic signature of the cascaded orbital hybridization, which constructs efficient dual‐channel charge transport pathways. Faster kinetics arise from combined contributions of electron‐rich Ag° clusters that facilitate rapid interfacial electron transfer and substitutional Ag^+^ ions anchoring and stabilizing ^*^COOH intermediates at O_V_‐rich domains. Electrochemical double‐layer capacitance (Cdl, Figure 3c) analysis corroborates this conclusion, showing that Ag_1_/Ag_n_‐R‐CeO_2_ achieves the highest Cdl (6.30 mF·cm^−2^), as derived from cyclic voltammetry (CV) measurements in a non‐Faradaic potential region (Figures S26–S28), indicative of an expanded electrochemically active surface area (ECSA) [54, 55, 56]. Hierarchical porosity combined with highly exposed {110} facets provides the structural foundation for this enhancement by compartmentalizing the dual‐scale Ag species into stable and accessible microdomains. In contrast, catalysts containing only one type of Ag species (Ag_1_ or Ag_n_) suffer from limited ECSA due to either poor electronic conductivity or insufficient active site density, confirming that the high current output is enabled by the synergistic coexistence of both types of Ag sites, which is uniquely facilitated by the dual‐mode interaction on the {110} facet.

The high FE_CO_ confirms efficient CO_2_ activation. However, for integrated DMC electrosynthesis, the catalyst must concurrently and stably supply both CO and ^*^CH_3_O^−^ intermediates. We therefore evaluated if our dual‐function architecture meets this challenge. To underscore that our facet‐specific design is crucial for meeting this integrated demand, a control experiment was performed using a commercial gold foil cathode with a geometric area identical to our catalyst electrode under otherwise identical conditions. The gold foil achieved a FE_CO_ of 82.41% (Figure S29), consistent with literature reports for pure Au in CO_2_RR yet only a FE_DMC_ of 61.09% (Figure S30). This discrepancy originates from the combined effects of inferior CO generation efficiency and limited CO utilization efficiency of the gold foil. Compared to our Ag_1_/Ag_n_‐R‐CeO_2_ catalyst, the gold foil exhibits inherently lower FE_CO_ (82.41% vs 92.51%) which reduces the total amount of CO available for downstream coupling. As a pure metallic catalyst, gold also lacks the {110} facet‐specific dual‐scale Ag synergy and O_V_‐rich environment that characterize our catalyst. Such structural differences lead to lower effective utilization of generated CO, where CO species on gold surfaces tend to remain adsorbed or undergo minor oxidation prior to diffusion into the solution phase, limiting their participation in the Pd/Br^−^‐mediated coupling reaction. In contrast, our catalyst optimizes CO generation both quantitatively with higher FE_CO_ and qualitatively through enhanced CO accessibility via electronic coupling, ensuring a steady supply of active CO that efficiently engages in cascaded coupling.

We then assessed the catalyst performance within the integrated single membrane‐free electrolyzer for direct DMC synthesis. Under optimized potentiostatic conditions, as shown in Figure 3d, Ag_1_/Ag_n_‐R‐CeO_2_ achieved an FE_DMC_ of 88.53%, significantly surpassing the performance of control catalysts: Ag_1_‐G‐CeO_2_ (85.10%), Ag_n_‐C‐CeO_2_ (78.11%), and undoped R‐CeO_2_ (73.68%). The FE_DMC_ was calculated based on a two‐electron process per DMC molecule (see 1. Experiments Section in Supporting Information for details). The moles of DMC produced were quantified by GC using an internal standard method (calibration curve R^2^> 0.999, Figure S2), with its formation unambiguously confirmed by GC‐MS and ^1^H NMR (Figure S31 and Table S8, Figures S32–S34). Online gas analysis and liquid product screening identified methyl formate as the only major by‐product, with a FE below 4%, ensuring no significant unaccounted electron consumption.

To unequivocally confirm that the high performance stems from the facet‐specific integration of dual‐scale silver rather than a mere physical mixture of Ag_1_ and Ag_n_ sites, we prepared and evaluated a 1:1 physical mixture of Ag_1_‐G‐CeO_2_ and Ag_n_‐C‐CeO_2_. Results from the physical mixture catalyst confirm the inferior performance with only 78.06% FE_DMC_ and 33.5 mA·cm^−2^ (Figure S35), substantially lower than values obtained with the integrated Ag_1_/Ag_n_‐R‐CeO_2_ catalyst (88.53% and 52.5 mA·cm^−2^). Marked by a pronounced performance gap, these results demonstrate that the simple physical blending of both Ag species is insufficient to replicate the catalytic excellence. In the physical mixture, the Ag_1_ and Ag_n_ sites are spatially and electronically isolated on distinct CeO_2_ supports, preventing the efficient electronic coupling and cooperative action that are hallmarks of the Ag_1_/Ag_n_‐R‐CeO_2_ architecture. These findings definitively prove that the {110} facet‐specific electronic environment, which co‐stabilizes both species within a single, integrated interface and enables their electronic communication via orbital hybridization, is indispensable for creating the synergistic ensemble that concurrently optimizes CO_2_ activation and electron transfer.

Long‐term operational durability was validated over 20 continuous cycles at approximately −50 mA·cm^−2^, during which both current density and FE_DMC_ remained stable, with the final performance retaining 77.64%. Post‐reaction quantitative characterization confirms the structural origin of this resilience. Post‐reaction ICP‐OES analysis measured an Ag content of 2.3 wt.%, down from the initial 3.0 wt.% (Table S9). Notably, this level of performance retention significantly outperforms the relative Ag loss, indicating that the intrinsic activity per remaining Ag site is exceptionally well‐preserved. Robust {110} facet‐specific electronic coupling thereby plays a critical role in maintaining the structural and electronic integrity of the active Ag species. The post‐reaction EXAFS fitting resultss (Figure 3e; Figures S36–S39 and Table S10) reveal the Ag–O coordination number (3.3) and bond length (2.28 Å) are nearly identical to those of the fresh catalyst (3.2, 2.09 Å), while the Ag–Ag coordination number (1.3) and bond length (2.91 Å) show no significant deviation from the initial state (1.4, 2.88 Å). These results confirm the local chemical environment of both atomic Ag_1_ and metallic Ag_n_ clusters remains intact after long‐term operation, with no detectable aggregation or structural rearrangement. XPS measurements (Figure 3f; Figure S16b) further highlight the stability of the catalyst's electronic structure, with supporting evidence from the marked increase in O_V_ content and Ce^3+^ fraction. The O_V_ content increased from 16.28% to 70.39% (Figure S16b), and the Ce^3+^ fraction rose from 25.30% to 27.80% (Figure 3f), indicating a redox‐active cycle that regenerates O_V_ and maintains electron conductivity. TEM imaging (Figure S40) confirmed that the rod‐like morphology remained intact, with no observed Ag aggregation. Furthermore, to demonstrate that merely having a high O_V_ concentration is insufficient for long‐term performance, we compared the stability of Ag_1_‐G‐CeO_2_, which has a similar O_V_ content mechanism but lacks the dual‐scale Ag structure. After the same 20‐cycle durability test, the FE_DMC_ retention for Ag_1_‐G‐CeO_2_ was only 63.12%, significantly lower than the 77.64% for Ag_1_/Ag_n_‐R‐CeO_2_ (Figure 3g). This comparative result underscores that the stable electronic coupling between atomically dispersed Ag_1_ and metallic Ag_n_ clusters, enabled by {110} facet orbital hybridization, is crucial for maintaining the integrity and synergistic function of the active sites under operating conditions, going beyond simply increasing O_V_ concentration. Besides, the catalyst synthesis exhibits promising reproducibility. Three independent batches of Ag_1_/Ag_n_‐R‐CeO_2_ showed FE_DMC_ values of 88.53%, 87.09%, and 89.52%, with current density variation below 3%.

To validate the generality of our catalytic strategy, we further explored the applicability of this system for the synthesis of other carbonates. When the methanol feed was replaced by ethanol, the integrated system successfully converted CO_2_ and ethanol into DEC. A FE for DEC of 30.51% was achieved at the optimized potential while maintaining a current density of approximately 7.5 mA·cm^−2^ (Figure S41). The formation of DEC was unambiguously confirmed by GC‐MS (Figure S42 and Table S11). Although the efficiency for DEC is currently lower than that for DMC, this result decisively demonstrates that our designed dual‐scale Ag‐CeO_2_ catalytic interface is capable of activating different primary alcohols and facilitating C–O coupling. The difference in yield is primarily attributed to the slower nucleophilic attack kinetics of ethanol on the Pd(CO)Br_2_ intermediate and its lower degree of dissociation in the electrolyte, as corroborated by DFT calculations, which identified a substantially ΔG for the solution‐phase ethoxide coupling step (∼14 kcal·mol^−1^ higher than for methoxide, Figure S43). Importantly, these results demonstrate the transferability of our catalyst design principle, enabling the electrosynthesis of diverse value‐added dialkyl carbonates beyond a single product.

Figure 3h benchmarks the Ag_1_/Ag_n_‐R‐CeO_2_||Pd(CO)Br_2_ system against the most advanced CO_2_‐to‐DMC and CO‐to‐DMC routes in the accompanying literature (see Table S12 for a comprehensive comparison). When benchmarked against state‐of‐the‐art CO_2_‐to‐DMC systems, our catalytic system's performance stands out. Notably, this study represents the first reported example of an Ag‐doped CeO_2_ catalyst employed for DMC synthesis in a single membrane‐free electrolyzer, distinguishing it from systems based on noble metals or single‐metal oxides in the literature. For instance, Lee et al. [17] and Li et al. [57] reported FE_DMC_ of 60% and 80%, respectively, both operating at around 12 mA·cm^−2^. Our system achieves a superior FE_DMC_ of 88.53% at a significantly higher current density of 47–53 mA·cm^−2^ without external CO [58]. Furthermore, our system exhibits markedly superior stability compared to systems suffering rapid activity loss [59]. Overall, the integrated system achieves a stable DMC production rate of 0.822 mmol·h^−1^·cm^−2^ (37.9 mg·h^−1^·cm^−2^) at 88.53% FE_DMC_, an energy consumption of 2.68 kWh·kg^−1^/h of operation (for calculation details, see the Experiments Section in the Supporting Information). Calculation of this energy efficiency metric based on the total cell voltage of 3.4 V, consistent with the potentiostatic cathode potential of −2.2 V, aligns with reporting standards in top‐tier studies. In summary, the comprehensive performance profile, underpinned by a morphology‐engineered catalytic architecture that harnesses facet‐specific orbital hybridization, sets a new benchmark and provides a versatile paradigm for CO_2_ electrosynthesis.

### 2.4 Regulating Intermediate Dynamics via Dual‐Scale Silver for Efficient CO_2_‐to‐DMC Conversion

**FIGURE 4:**
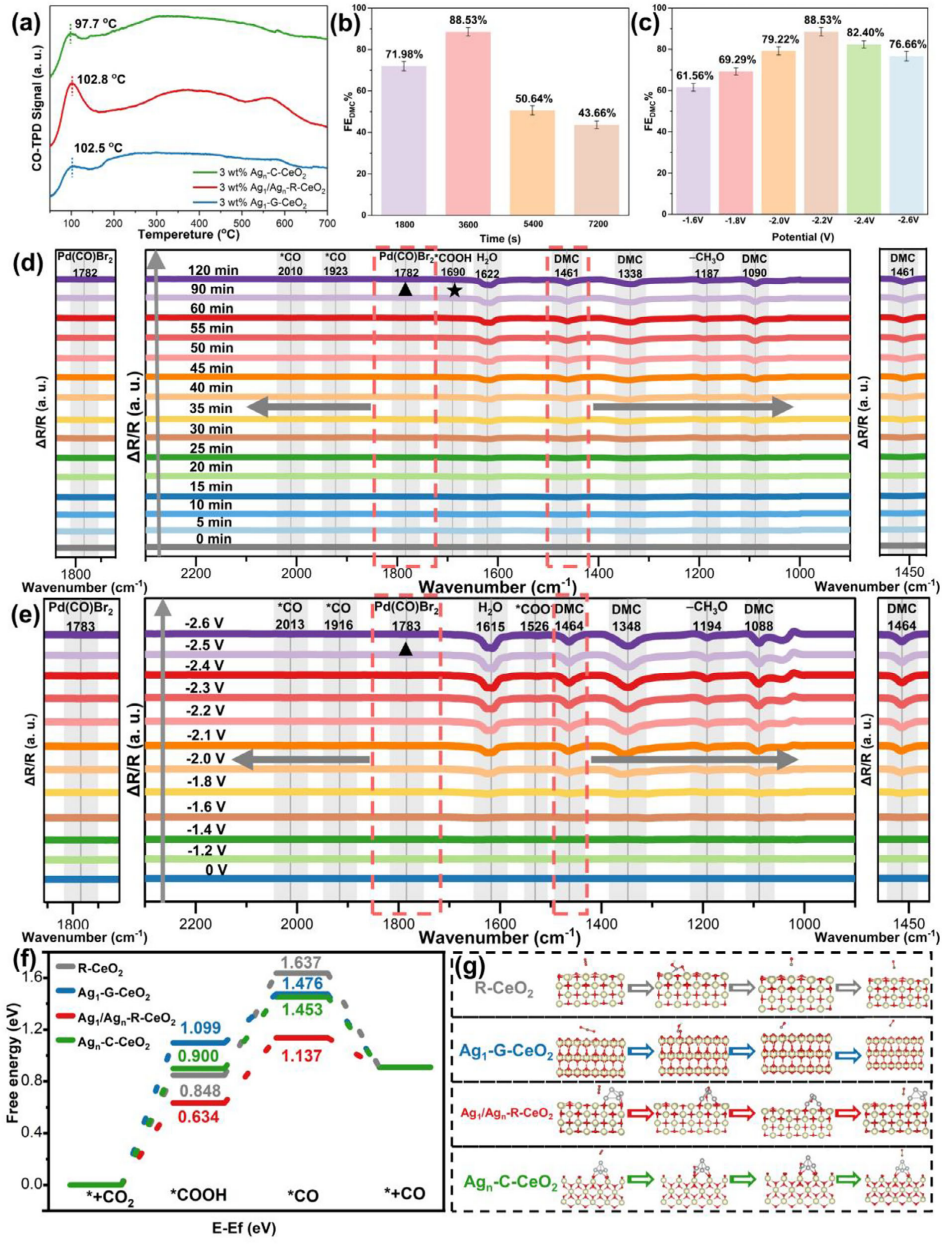
Dual‐scale Ag species orchestrate intermediate dynamics for efficient CO_2_‐to‐DMC electrosynthesis: (a) CO‐TPD spectra of Ag‐based catalysts showing distinct CO desorption features, (b) FE_DMC_ as a function of reaction time at −2.2 V, the data are averaged over three repeated measurements, Error bars indicate the standard deviation, (c) FE_DMC_ as a function of applied potential, the data are averaged over three repeated measurements, Error bars indicate the standard deviation, (d) Time‐dependent in situ ATR–FTIR spectra tracking key intermediates (^*^COOH, ^*^CO, Pd(CO)Br_2_, DMC) during electrolysis at −2.2 V over Ag_1_/Ag_n_‐R‐CeO_2_, (e) Potential‐dependent in situ ATR–FTIR spectra showing the evolution of intermediates, ▲ denotes key intermediates and products, (f) DFT‐calculated ΔG diagrams for the CO_2_ reduction pathway on different catalysts, (g) Proposed intermediates for CO_2_ reduction to DMC catalyzed by different catalysts.

To unravel how the facet‐specific dual‐mode MSI dynamically orchestrates the complex reaction network, we systematically probed the operational landscape. The FE_DMC_ at −2.2 V followed a characteristic trajectory over time (Figure 4b), rising from 71.98% at 0.5 h to a peak of 88.53% at 1 h, before declining to 43.66% at 2 h. Optimal performance at 88.53% FE_DMC_ represents the perfect balance between intermediate conversion efficiency and structural integrity of the orbitally hybridized catalytic interface. To elucidate the origin of the performance decline after 1 h, we investigated the evolution of the reaction system. Quantitative analysis via ICP‐OES confirmed a continuous consumption of Br^−^ ions over time (Table S13), which is integral to the formation of the Pd(CO)Br_2_ intermediate. Concurrently, in situ ATR–FTIR spectroscopy revealed the progressive accumulation of Pd(CO)Br_2_ beyond the 1 h optimum. We therefore attribute the late‐stage FE_DMC_ drop to a kinetic bottleneck in the catalytic cycle. The increasing demand for Br^−^ to sustain the Pd‐mediated coupling, coupled with the saturation of Pd active sites by excess Pd(CO)Br_2_, collectively perturbs the delicate balance of the reaction network, leading to the observed efficiency decay. Voltage‐dependent performance mapping (Figure 4c) further defined a distinct operational window. The peak efficiency emerged uniquely at −2.2 V, where the kinetics of CO_2_ activation at substitutional MSI‐generated Ag_1_–O_V_ sites and electron delivery from Ag_n_ clusters reached an optimal balance. Deviations from this potential, to either −1.6 V with insufficient electron flux yielding a FE_DMC_ of 61.56%, or −2.6 V where excessive electron flux perturbs critical Ag─O bonding, resulting in a FE_DMC_ of 76.66%, led to significant performance loss.

Regulation of intermediate dynamics by the dual‐scale Ag species gives rise to superior and tunable performance, with this mechanism being unequivocally validated by a suite of characterization techniques. CO‐TPD profiles (Figure 4a) revealed that Ag_1_/Ag_n_‐R‐CeO_2_ exhibits a unique CO desorption peak at 102.8°C, reflecting a finely tuned Ag–CO binding strength governed by the hybridized Ag^+^–O–Ce^3+^ electronic structure at the {110} interface. Critically, the peak area was 2.96‐fold and 2.69‐fold larger than that of Ag_1_‐G‐CeO_2_ at 102.5°C and Ag_n_‐C‐CeO_2_ at 97.7°C, respectively. Greater peak area directly evidences how the dual‐mode MSI on {110} facets delivers not only optimal Ag–CO binding energy but also enhanced active site density, thereby ensuring a steady and substantial flux of CO intermediates for downstream coupling. The structural origins of these differences are clear. Ag_1_‐G‐CeO_2_ lacks adsorptive MSI‐stabilized Ag_n_ clusters, which are essential for establishing an efficient electron pathway for CO desorption, while Ag_n_‐C‐CeO_2_ suffers from severe Ag nanoparticle aggregation, drastically reducing the number of accessible active sites for CO adsorption and creating a non‐uniform interfacial environment.

Time‐resolved in situ ATR–FTIR spectroscopy at −2.2 V (Figure 4d) provided real‐time insights into how the dual‐mode MSI orchestrates the evolution of key intermediates. Background spectra were collected from the NaBr‐methanol electrolyte under a CO_2_ atmosphere. The evolution of key intermediates was then quantified by normalizing the peak area of each species to its maximum intensity observed during the experiment, with that maximum defined as 100%. Time‐dependent spectral analysis further revealed that Ag_1_/Ag_n_‐R‐CeO_2_ exhibits faster accumulation of CeO_2_–OH (3388 cm^−1^, CO_2_ activation) and adsorbed –CH_3_ (2949, 2838 cm^−1^, methanol activation) compared to R‐CeO_2_ (Figure S44). For Ag_1_/Ag_n_‐R‐CeO_2_, the ^*^COOH intermediate characterized by its C = O stretching vibration at 1690 cm^−^^1^ built up rapidly [60], reaching 40.28% of its maximum peak area within 0.5 h, facilitated by the highly active Ag^+^–O–Ce^3+^ sites created by substitutional MSI. At this early stage, the signals for CO at 2010 cm^−1^ for Ag_1_‐bound CO and 1923 cm^−1^ for Ag_n_‐bound CO remained weak, with the signal at 1923 cm^−1^ being only 8.34% of its maximum intensity [61]. Weak CO signals at this stage reflect delayed ^*^COOH to ^*^CO conversion arising from initially inadequate electronic coupling from the Ag_n_ clusters, a process mediated by adsorptive MSI. By 1 h, the electronic coupling between Ag_n_ clusters and the CeO_2_ support, enabled through Ag^0^–Ag^0^ d–d orbital coupling during the third hybridization stage, had facilitated accelerated electron delivery to the interface, driving a 65% conversion of ^*^COOH to ^*^CO and boosting the Pd(CO)Br_2_ signal at 1782 cm^−1^ to 86.79% of its peak intensity within a mere 5 min. Following this rapid cascade, complete DMC formation was observed at 1090 cm^−1^, corresponding to the peak FE_DMC_. The Pd(CO)Br_2_ band persisted for over 45 min, and its intensity exhibited a direct correlation with the final FE_DMC_, underscoring its role as a crucial persistent intermediate. Beyond the 1 h optimum, the continued accumulation of surface intermediates, including Pd(CO)Br_2_, no longer efficiently converts to product, directly coinciding with the FE_DMC_ decline and highlighting that optimal DMC yield requires not only sufficient CO supply but also balanced coupling kinetics. In stark contrast, pristine R‐CeO_2_, which lacks MSI, showed markedly slower COOH buildup, reaching only 58.34% of the intensity observed on Ag_1_/Ag_n_‐R‐CeO_2_ (Figure S45a). It also exhibited 54.87% weaker CO signals, and consequently, lower Pd(CO)Br_2_ accumulation, yielding only 70.52% of the DMC intensity achieved by the optimized catalyst. Additionally, a weak absorption band at ∼1620 cm^−1^ is observed in the spectra, which is assigned to the O‐H bending vibration of H_2_O. As a by‐product of the DMC synthesis reaction (CO_2_ + CH_3_OH → DMC + H_2_O), H_2_O is generated throughout the reaction process [62, 63, 64, 65, 66]. Marked performance differences definitively establish Ag species as essential components for constructing the highly active interface needed for efficient intermediate generation and conversion.

Voltage‐dependent in situ ATR–FTIR analysis (Figure 4e) further underscored the role of MSI as a dynamic regulator that orchestrates the reaction pathway by controlling electron flow. Complementing this temporal insight, voltage‐dependent spectra (Figure S46) provided direct evidence of enhanced signal intensities for CeO_2_–OH (3351 cm^−1^) and adsorbed –CH_3_ (2946, 2835 cm^−1^) on Ag_1_/Ag_n_‐R‐CeO_2_ at the optimal potential of −2.2 V vs R‐CeO_2_, confirming its superior, potential‐tunable activation of both CO_2_ and methanol precursors. At −1.6 V, the weak electron flux from the Ag_n_ clusters (due to an insufficient driving force) resulted in a negligible ^*^CO signal, which severely limited the downstream coupling steps and restricted the FE_DMC_ to 61.56%. At the optimal −2.2 V, the balanced interplay between substitutional MSI (optimizing ^*^COOH activation at Ag_1_ sites) and adsorptive MSI (ensuring efficient ^*^CO formation and desorption via Ag_n_ clusters) pushed the FE_DMC_ to its peak of 88.53%. At the excessive potential of −2.6 V, the heightened reductive environment disrupted the precise Ag─O bonding crucial for substitutional MSI, diverting electrons toward proton reduction and consequently lowering the FE_DMC_ to 76.66%. In stark contrast, pristine R‐CeO_2_, which lacks such MSI‐mediated regulation, failed to achieve meaningful optimization with applied potential (Figure S45b). Its FE_DMC_ remained poor, staying below 55% throughout the tested voltage window, which unequivocally highlights the indispensable role of the engineered Ag sites.

At the cathode interface, both CO_2_RR and methanol‐to‐^*^CH_3_O^−^ conversion occur simultaneously. These two processes provide critical precursors for the subsequent coupling reaction and are therefore of great significance for the efficient synthesis of DMC via the cascaded coupling system. DFT calculations under experimentally relevant conditions with a pH of 6 and at 298.15 K provided the thermodynamic foundation for the observed kinetics, unequivocally validating the central role of the dual‐mode MSI (Figure 4f,g). Critical thermodynamic analysis confirms that the rate‐determining step of the cathodic CO_2_RR is the transformation of CO_2_ to ^*^COOH. This conclusion is supported by the consistent energy barrier trend across all catalysts. Ag_1_/Ag_n_‐R‐CeO_2_ achieved the lowest ΔG of 0.63 eV, outperforming Ag_n_‐C‐CeO_2_ at 0.90 eV, R‐CeO_2_ at 0.85 eV, and Ag_1_‐G‐CeO_2_ at 1.10 by 0.27, 0.22, and 0.47 eV, respectively. Facet‐specific orbital hybridization drives this significant reduction by enabling a synergistic catalytic cycle that preactivates and bends the CO_2_ molecule at O_V_ (Figures S47–S50) and stabilizes the Ag^+^–O–Ce^3+^ motifs via strong p–d orbital mixing to lower the ^*^COOH stabilization energy, a process initiated by electronic coupling between Ag^0^ 4dx^2^‐y^2^ and Ce 5d orbitals. This coupling promotes Ce^4+^ reduction to Ce^3+^ and O_V_ generation. Bader charge analysis confirmed a net electron flow from the Ag species to adjacent Ce sites, corresponding to a gain of 2.24 to 2.38 e^−^ per Ce atom. Such electron redistribution quantitatively facilitates ^*^COOH formation and is fully consistent with the rapid kinetics observed by in situ ATR–FTIR. In addition to regulating CO_2_RR, the cathode material is also crucial for the generation of ^*^CH_3_O^−^ from methanol. For the concurrent methanol‐to‐^*^CH_3_O^−^ conversion (Figure S51), the dual‐mode MSI finely tunes the electron density of the Ag–O_V_ ensemble, yielding a near‐thermoneutral ΔG of −0.40 eV for ^*^CH_3_O^−^ generation. Over‐stabilization and poisoning of the surface by ^*^CH_3_O^−^ are thereby avoided, preserving intermediate mobility for diffusion to Pd coupling sites. In stark contrast, the larger exothermicity observed on Ag_n_‐C‐CeO_2_ at −1.69 eV and Ag_1_‐G‐CeO_2_ at −1.09 eV leads to strong adsorption and severe methoxy poisoning, which inhibits the overall process. The simultaneous optimization of the thermodynamics for both the CO_2_ reduction and methanol‐to‐^*^CH_3_O^−^ processes on Ag_1_/Ag_n_‐R‐CeO_2_, therefore, ensures a rapid and efficient supply of the CO feedstock, which is a critical prerequisite for sustaining the downstream solution‐phase coupling cycle that dictates the overall DMC production rate.

### 2.5 A Pd(CO)Br_2_‐Centered Mechanism for CO_2_‐to‐DMC Electrosynthesis Driven by MSI‐Generated CO

**FIGURE 5:**
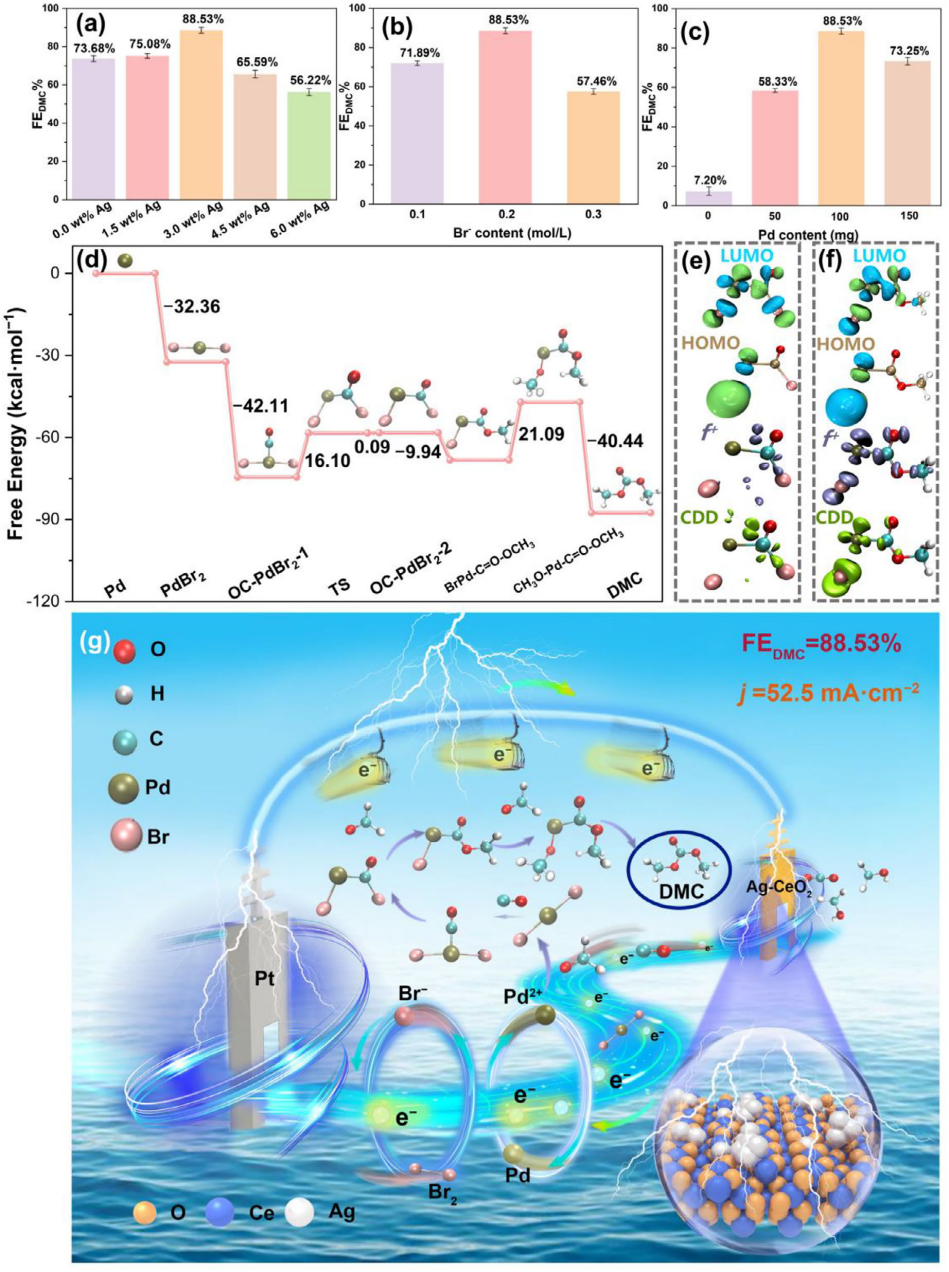
Full‐cell performance optimization and the Pd(CO)Br_2_‐centered mechanism for CO_2_‐to‐DMC electrosynthesis: (a) Optimization of FE_DMC_ as a function of Ag loading on the CeO_2_ support, the data are averaged over three repeated measurements, Error bars indicate the standard deviation, (b) Optimization of FE_DMC_ as a function of Br^−^ concentration in the electrolyte, the data are averaged over three repeated measurements, Error bars indicate the standard deviation, (c) Optimization of FE_DMC_ as a function of Pd/C catalyst amount, the data are averaged over three repeated measurements, Error bars indicate the standard deviation, (d) DFT‐calculated free energy profile for the Pd(CO)Br_2_‐mediated catalytic cycle, identifying the rate‐determining step (C–O coupling), (e)–(f) Electronic structure analysis of the key Pd(CO)Br_2_ and CH_3_O–Pd(CO)Br intermediate: HOMO‐LUMO distributions, Fukui function (*f^+^*), and charge density difference map, (g) Schematic illustration of the synergistic full‐cell operation, highlighting the spatially separated cathode and anode reactions integrated by the solution‐phase Pd(CO)Br_2_‐mediated catalytic cycle.

Performance optimization confirms that each component of this multi‐phase system requires precise balancing to maintain synergy (Figure 5a–c). The entire reaction cascade originates from the efficient generation of CO, the essential carbon feedstock for DMC synthesis, at the dual‐scale Ag‐CeO_2_ interface. XRD analysis (Figure S52) confirms that the fluorite structure of CeO_2_ is maintained across Ag loadings from 1.5 to 6.0 wt.%. A loading of 3.0 wt.% yields the optimal O_V_ density and R_ct_, resulting in the highest FE_DMC_ of 88.53%; higher loadings lead to Ag aggregation and performance loss. Similarly, the Br^−^ concentration must be tuned to 0.2 m to match the CO production rate without causing competitive adsorption on the oxygen vacancies critical for CO_2_ activation. The Pd/C dosage is optimized at 100 mg to efficiently transform the CO flux while avoiding catalyst aggregation. Thus, the combination of parameters (3.0 wt.% Ag, 0.2 m Br^−^, 100 mg Pd/C) collectively maximizes the efficiency of the entire cascade process.

Notably, the dual‐scale Ag_1_/Ag_n_‐R‐CeO_2_ cathode serves as the foundational driver of the cascade by mediating efficient CO_2_RR to generate CO and facilitating the formation of CH_3_O^−^, both of which are indispensable precursors for DMC synthesis. Complementary gold foil control experiments further corroborate the structural advantage of Ag_1_/Ag_n_‐R‐CeO_2_: despite the gold foil's ability to reduce CO_2_ to CO, its inferior performance in FEDMC (61.09% vs 88.53% of Ag_1_/Ag_n_‐R‐CeO_2_) highlights the critical role of the dual‐scale Ag‐CeO_2_ interface in balancing CO generation and accessibility for downstream coupling. However, efficient DMC synthesis cannot be achieved by the cathode alone. The anodic oxidative generation of Br_2_ and the subsequent coupling reaction mediated by the heterogeneous Pd/C catalyst in solution are equally essential, as Br_2_ acts as the oxidant for Pd activation and the solution‐phase coupling of CO with CH_3_O^−^ directly determines the final product yield. To clarify the intrinsic mechanism of this multistep cascade, especially the solution‐phase coupling process that complements the cathodic reaction, we performed DFT calculations to unravel the detailed catalytic cycle.

These DFT calculations at the B3LYP/Def2‐TZVP level, with implicit solvation (methanol) at 298 K (see 3.2 Computational Details of Gaussian in Supporting Information for full details), unveil the catalytic cycle, beginning with the oxidative addition of Br_2_ to Pd (ΔG = −32.36 kcal·mol^−1^). Subsequent CO insertion then forms the key Pd(CO)Br_2_ intermediate (ΔG = −42.11 kcal·mol^−1^). Bond order analysis identifies the C─Br bond (0.954) as the preferred site for nucleophilic attack. Transformation via a transition state yields Pd(CO)Br_2_‐2, which undergoes methoxide attack to form CH_3_O–Pd(CO)Br (ΔG = −9.94 kcal·mol^−1^). The subsequent nucleophilic addition of a second CH_3_O^−^ to CH_3_O–Pd(CO)Br, identified as the rate‐determining step of this Pd‐mediated solution‐phase cycle with a ΔG of 21.09 kcal·mol^−1^, produces CH_3_O–Pd(CO)–CH_3_O. Resulting CH_3_O–Pd(CO)–CH_3_O then undergoes reductive elimination to form DMC with a ΔG of −40.44 kcal·mol^−1^. Overall, the Pd‐mediated pathway achieves an 82% reduction in the activation barrier relative to the uncatalyzed route (118.29 kcal·mol^−1^), a kinetic enhancement consistent with the long‐lived in situ ATR–FTIR signature of Pd(CO)Br_2_ at 1782 cm^−1^.

To unravel the origin of the lower FE for DEC compared to DMC, we performed DFT calculations on the solution‐phase Pd‐mediated coupling cycle (Figure S43). The reaction pathways for both carbonates are identical, with the rate‐determining step being the nucleophilic addition of the second alkoxide to the CH_3_O–Pd(CO)Br or CH_3_CH_2_O–Pd(CO)Br intermediate. A direct comparison reveals that this critical step has a significantly higher ΔG for DEC formation (35.0 kcal·mol^−1^) than for DMC formation (21.1 kcal·mol^−1^). Such a marked energy difference, amounting to ∼14 kcal·mol^−1^, reflects the combined effects of greater steric hindrance and reduced nucleophilicity of ethoxide (CH_3_CH_2_O^−^) vs methoxide (CH_3_O^−^). Consequently, the kinetic bottleneck for DEC production is decisively located in the solution‐phase C–O coupling, while our dual‐scale Ag‐CeO_2_ cathode proficiently fulfills its role by efficiently generating the requisite CO and alkoxide intermediates from CO_2_ and the respective alcohol. Together, these mechanistic insights delineate the functional domains within our integrated system and establish the Ag‐CeO_2_ architecture as a versatile platform for activating different alcohols, where the ultimate carbonate yield depends on the intrinsic kinetics of the subsequent homogeneous reaction.

The high reactivity of the Pd(CO)Br_2_ intermediate is rooted in its electronic structure (see Figures S53 and S54 for full details). As visualized in Figure 5e, HOMO–LUMO distributions and Fukui function analysis (*f* ^+^ = 0.2662) pinpoint the C─Br bond as the key electrophilic site, primed for nucleophilic attack by CH_3_O^−^. Charge density difference maps further show electron accumulation at the C─Br bond and depletion at the Pd center, enhancing its attraction to the methoxy nucleophile. The increase in the HOMO–LUMO gap from 61.8 kcal·mol^−1^ for Pd(CO)Br_2_ to 74.7 kcal·mol^−1^ for CH_3_O–Pd(CO)Br rationalizes the kinetic penalty for the first ligand exchange step. Notably, the rate‐determining intermediate CH_3_O–Pd(CO)Br retains key electronic features inherited from its Pd(CO)Br_2_ precursor (Figure 5f). Fukui function analysis confirms the electrophilic character of the C─Br bond (*f* ^+^ = 0.2411), highlighting a conserved electronic profile across catalytic intermediates. Maintained through the electronic influence of Pd, this persistent electrophilicity secures the C─Br bond as the preferred site for nucleophilic attack throughout the catalytic cycle. The significant electron depletion at the Pd center across all key intermediates not only enhances the electrophilicity of the C─Br bond but also facilitates the final reductive elimination by stabilizing the electron‐rich Pd center in the transition state, thereby directly contributing to the lowered overall energy barrier of the Pd‐mediated cycle.

In summary, the catalytic cycle operates via a redox‐neutral sequence that integrates heterogeneous cathodic electrochemistry and molecular coupling mediated by the Pd/C catalyst in solution. Efficient CO generation initiates at the dual‐scale Ag_1_/Ag_n_‐R‐CeO_2_ {110} cathode, where facet‐specific electronic coupling between Ag^0^ 4d and Ce 5d orbitals facilitates O_V_ formation. Simultaneously, p‐d orbital interaction between Ag^+^ 4d and O 2p orbitals optimizes CO adsorption. Continuous electron supply for CO production is sustained through electronic communication among Ag^0^ sub‐nanoclusters, a key feature of the dual‐scale Ag configuration. The resulting electrogenerated CO diffuses into solution and adds oxidatively to Pd and Br_2_, forming the pivotal Pd(CO)Br_2_ intermediate. Its distinct electronic structure, with an electrophilic C─Br bond and an electron‐depleted Pd center, directs nucleophilic attack by CH_3_O^−^. The cycle progresses through a solution‐phase rate‐determining second methoxide addition to CH_3_O–Pd(CO)Br, which has a calculated barrier of 21.09 kcal·mol^−1^, followed by reductive elimination to release DMC. Throughout this integrated pathway, redox neutrality is maintained by balancing the electrons consumed in cathodic CO_2_ reduction with those released during anodic bromide oxidation, thereby avoiding parasitic reactions. Collectively, the entire mechanism encompassing cathode electronic coupling‐regulated CO production and Pd‐mediated C–O coupling is governed by the electronic properties of key intermediates at each step, which underlines the indispensable synergistic role of the dual‐scale Ag‐CeO_2_ {110} cathode and the Pd/Br^−^ mediator in achieving efficient, selective DMC synthesis.

## 3 Conclusions

We present a design paradigm that mitigates classic electrocatalytic trade‐offs by decoupling catalytic functions through facet‐specific electronic coupling. Targeting the {110} facets of CeO_2_, we engineered a facet‐specific electronic interface that co‐stabilizes atomically dispersed Ag_1_ and sub‐nanometer Ag_n_ clusters. The resulting dual‐scale architecture forms a synergistic ensemble rather than a random mixture. Within this ensemble, a functional division of labor is established, with CO_2_ activation occurring at atomic sites and electron transport proceeding through metallic clusters. This division is enabled by specific orbital interactions, the nature of which has been verified through quantitative bader and ICOHP calculations. Demonstrating its intrinsic superiority, this interface achieves 92.51% FE_CO_ in a standard H‐type cell. Leveraging this fundamentally integrated and efficient precursor supply, the catalyst within a membrane‐free paired electrolyzer enables direct DMC synthesis with 88.53% FE_DMC_ at an industrial current density of 52.5 mA·cm^−2^, maintaining stable operation for 20 h (<11% activity loss), outperforming recent advanced systems, which typically operate below 80% efficiency at current densities around 12 mA·cm^−2^. Control experiments, including comparisons with gold foil and physical mixtures, provide evidence that the high performance stems from the in situ electronic synergy of the dual‐scale Ag, rather than merely from the coexistence of sites. Operationally, this high efficiency translates to a stable space‐time yield of 37.9 mg·h^−1^·cm^−2^ while maintaining an energy consumption of 2.68 kWh·kg^−1^. The catalytic interface also facilitates DEC synthesis, confirming the generality of the design principle beyond a single product. Mechanistic studies reveal that the high performance is orchestrated by the dual‐scale Ag species regulating intermediate dynamics, with a C–O coupling pathway that proceeds via preferential C–Br cleavage. More broadly, this study demonstrates that facet‐governed electronic coupling offers a viable and rational strategy to design multifunctional interfaces for complex electrosynthesis, and could contribute to a framework for integrating catalyst design with molecular mediation to efficiently produce value‐added chemicals from CO_2_.

## Author Contributions

Y.W. and J.H. supervised this study. J.H. secured funding. Y.W. and B.T. designed the experiments. Y.W. performed the material synthesis and structural characterizations (including TEM, XRD, XPS). B.T. conducted the electrochemical measurements (LSV, EIS, stability tests) and data analysis. Y.T. and W.W. contributed to the DFT calculations and theoretical analysis. Y.W. and X.M. participated in the catalytic performance evaluation and result verification. Y.L. provided key resources and oversight. Y.W., B.T., and J.H. co‐wrote the paper. All authors discussed the results and commented on the manuscript.

## Conflicts of Interest

The authors declare no conflicts of interest.

## Supporting information




**Supporting File 1**: advs73776‐sup‐0001‐SuppMat.docx.

**Supporting File 2**: advs73776‐sup‐0002‐DataFile.zip.

## Data Availability

The data that support the findings of this study are available from the corresponding author upon reasonable request.
