# Supplementary material for: Orbital Hybridization‐Mediated Decoupling of Electrocatalytic Functions for Paired CO2 Electrosynthesis
Source: Adv Sci (Weinh). 2026 Jan 12;13(17):e22711. doi: 10.1002/advs.202522711 (PMC13042488; doi:10.1002/advs.202522711)
Supplement: Supplementary file 1 — Supporting File 1: advs73776‐sup‐0001‐SuppMat.docx. [file ADVS-13-e22711-s002.docx]

**Orbital Hybridization-Mediated Decoupling of Electrocatalytic Functions for Paired CO_2_ Electrosynthesis**

Youjia Wang^a^, Bochen Tian^b^, Yuxin Tian^b^, Wenchao Wang^b^, Xin Ma^b^, Yansheng Liu^b,*^, Junwei Hou^b,*^

*^a^ State Key Laboratory of Heavy Oil Processing, China University of Petroleum, Beijing 102249, China*

*^b^ State Key Laboratory of Heavy Oil Processing, China University of Petroleum (Beijing) at Karamay, Karamay, Xinjiang 834000, China*

1. **Experiments Section**

***Materials and reagents:*** Sodium bromide and silver nitrate were purchased from China Pharmaceutical Group Co. Ltd; Sodium hydroxide, Cerium(III) nitrate hexahydrate, methanol and anhydrous ethanol were purchased from Shanghai Titan Scientific Co. Ltd; hydrophobic carbon paper with microporous layer was purchased from Toray Industries.

***Synthesis of CeO_2_ and Ag-CeO_2_:***

**Table S1. Synthesis of CeO_2_ and Ag-CeO_2_.**

| **Sample** | **Morphology** | **Precursors (30 mL Deionized Water as Solvent)** | **Hydrothermal Conditions** | **Post-Treatment Procedures** |
| --- | --- | --- | --- | --- |
| **R-CeO_2_** | Rod | Ce(NO_3_)_3_·6H_2_O: 0.651 g; NaOH: 7.2 g | 100 ℃; 24 h | Washed with ethanol until pH reached 7; dried at 60 °C overnight; calcined in static air at 200 °C for 12 h. |
| **C-CeO_2_** | Cube | Ce(NO_3_)_3_·6H_2_O: 0.651 g; NaOH: 3.6 g | 180 ℃; 24 h | Washed with ethanol until pH reached 7; dried at 60 °C overnight; calcined in static air at 200 °C for 12 h. |
| **G-CeO_2_** | granular | Ce(NO_3_)_3_·6H_2_O: 0.651 g; NaOH: 9.0 g | 90 ℃; 24 h | Washed with ethanol until pH reached 7; dried at 80 °C for 8 h; calcined in static air at 600 °C for 5 h. |
| **Ag_1_/Ag_n_-R-CeO_2_** | Rod | Ce(NO_3_)_3_·6H_2_O: 0.651 g; NaOH: 7.2 g; AgNO_3_: varying masses | 100 ℃; 24 h | Washed with ethanol until pH reached 7; dried at 60 °C overnight; calcined in static air at 200 °C for 12 h. |
| **Ag_n_-C-CeO_2_** | Cube | Ce(NO_3_)_3_·6H_2_O: 0.651 g; NaOH: 3.6 g; AgNO_3_: varying masses | 180 ℃; 24 h | Washed with ethanol until pH reached 7; dried at 60 °C overnight; calcined in static air at 200 °C for 12 h. |
| **Ag_1_-G-CeO_2_** | granular | Ce(NO_3_)_3_·6H_2_O: 0.651 g; NaOH: 9.0 g; AgNO_3_: varying masses | 90 ℃; 24 h | Washed with ethanol until pH reached 7; dried at 80 °C for 8 h; calcined in static air at 600 °C for 5 h. |

***Preparation of Ag-CeO_2_/GDL electrode:*** 10 mg of powder was added to 200 μl of ethanol and 45 μl of Nafion solution, ultrasonicated for 5 min to mix well, and the solution was applied to hydrophobic carbon paper with microporous layer, dried naturally, and then sealed and bagged.

***Characterization:*** SEM Performed on a [ZEISS Sigma 300] microscope to observe the macroscopic morphology, particle size distribution, and surface topography of the samples; TEM and STEM conducted using a [JEOL JEM-F200 and Thermo Fisher Titan Themis G2 60-300] instrument, and TEM provides high-resolution images of crystal lattice fringes and internal defects, while STEM enables elemental mapping with nanoscale spatial resolution to confirm the uniform distribution of components; ICP-OES conducted using a [Agilent 5110(OES] instrument, which is used for quantitative analysis of major and minor metallic elements in the material, providing accurate bulk elemental content; XRD recorded on a [Rigaku SmartLab SE], which is used to identify crystalline phases, calculate lattice parameters, and determine crystallite size (via the Scherrer equation); XPS measured on a [Thermo Scientific K-Alpha] , which analyzes the elemental composition of the sample surface and the chemical valence states of target elements; EPR carried out on a [Bruker EMXPlus], which can detect oxygen vacancies and characterizes their local coordination environment; BET performed using a [Micromeritics ASAP 2460], which calculates the specific surface area, while the Barrett-Joyner-Halenda (BJH) model determines the pore size distribution and total pore volume; TPD conducted on a [Micromeritics AutoChem II 2920], this technique characterizes the type, density, and strength of surface active sites for CO adsorption by monitoring the desorbed gas signal during temperature ramping; *in situ* ATR–FTIR collected on a [Thermo Scientific Nicolet iS50],and the dynamic evolution of surface adsorbed species (e.g., reactants, intermediates, products) during the reaction process, revealing the reaction mechanism by identifying characteristic functional group peaks; SR Techniques utilized at [Spring8 BL14B2], and SR-based XAFS (including XANES and EXAFS) analyzes the local atomic structure of target elements under in situ conditions; GC-MS employed on a [Agilent 7890A GC-5975C MS], which is used for qualitative and quantitative analysis of products generated during reactions.

***Electrochemical CO_2_RR measurement:*** CO_2_RR for electrocatalytic CO and DMC production were conducted using a CHI660E electrochemical workstation (CH Instruments, USA), with Pt foil as the counter electrode and Ag/AgCl as the reference electrode in both setups. For CO production, an H-type electrolytic cell with a Nafion 117 membrane separating the cathodic and anodic chambers was used, with 0.2 M NaBr-H_2_O as the electrolyte. Prior to reaction, high-purity CO_2_ was bubbled through the electrolyte for at least 30 min to saturate it, and a mass flow controller maintained a CO_2_ flow rate of 50 sccm during the process. For DMC production, a single-compartment electrolytic cell was employed, with 0.2 M NaBr-CH_3_OH as the electrolyte. And the anode and cathode were horizontally positioned with a 2 cm spacing.

***Coupled anodic-cathodic electrolysis:***

A 1 × 1 cm^2^ Pt foil serves as the anode, while a 1.5 × 1.3 cm^2^ Ag-CeO_2_/GDL material functions as the cathode. An Ag/AgCl electrode is utilized as the reference electrode. The electrolyte comprises 20 ml of NaBr-CH_3_OH solution, and the electrocatalytic performance of the Ag-CeO_2_/GDL material is assessed based on the generation of dimethyl carbonate (DMC) in a 30 ml single-compartment electrolytic cell.

Prior to the reaction, CO_2_ is introduced into a single-compartment electrolytic cell at a flow rate of 50 ml/min for 30 minutes to displace the air within the cell. A potentiostat is then used to perform cyclic voltammetry (CV) scans over a voltage range of −3 ~ 3 V for 10 cycles to activate and reduce the electrode material, stabilizing the sample surface, with a scan rate of 50 mV/s.

linear sweep voltammetry (LSV) is initiated by applying a voltage range of −3 ~ 3 V to obtain the corresponding continuously varying current response, with a scan rate of 10 mV/s. The current density is observed as a function of voltage to determine the onset potential and the limiting current density, thereby identifying the potential range for CO_2_ catalytic reduction. Finally, the voltage is set to initiate the reaction.

To further investigate the merits of the Ag-CeO_2_/GDL material, we conducted the following tests. First, we performed electrochemical double-layer capacitance (Cdl) measurements to characterize the electrochemical active surface area (ECSA) of the electrode material. The testing method involved applying different scan rates to the non-Faradaic current region for Cdl testing. In the experiment, CV was used to set five different scan rates of 10 mV/s, 20 mV/s, 30 mV/s, 40 mV/s, and 50 mV/s for the Ag-CeO_2_/GDL material within a voltage range of 0.1 ~ 0.2 V for Cdl calculations. Second, we conducted electrochemical impedance spectroscopy (EIS) to analyze and reveal the interfacial charge transfer kinetics of different materials. The experiment was performed at a high frequency of 1 * 10^6^ Hz and a low frequency of 0.01 Hz, at an open-circuit voltage of −2.2 V. Third, we utilized the current-voltage data from the linear sweep voltammetry (LSV) tests to calculate the Tafel slopes for different samples. These tests provide a comprehensive assessment of the electrocatalytic performance of the Ag-CeO_2_/GDL material in CO_2_RR.

***The FE of DMC/DEC and CO were calculated by using the following equations:***

$\text{F}\text{E}_{\text{CO}}\text{=}\frac{\text{J}_{\text{CO}}}{\text{J}_{\text{total}}}\text{=}\frac{\text{ν}_{\text{CO}}\text{×}\text{N}\text{×}\text{F}}{\text{J}_{\text{CO}}}$ (1)^[1]^

$\text{J}_{\text{CO}}$: partial current density for CO production, A;

$\text{J}_{\text{total}}$: total current density, A;

$\text{N}$: the number of the electron transferred for forming a product molecule, which is 2 for CO;

$\text{ν}_{\text{CO}}$: the product rate of CO, which was calculated based on the equation of

$\text{ν}_{\text{CO}}\text{=}\frac{\text{V}_{\text{CO}}\text{×}\text{ν}_{\text{CO}_{\text{2}}}}{\text{V}_{\text{m}}}$ ,using the volume concentration of CO ($\text{V}_{\text{CO}}$) measured by GC, the CO_2_ flow rate ($\text{ν}_{\text{CO}_{\text{2}}}$) and the molar volume of gas ($\text{V}_{\text{m}}$);

*F*: Faradaic constant, 96485 C mol^−1^.

$\text{F}\text{E}_{\text{DMC}\text{/}\text{DEC}}\text{=}\frac{\text{2}\text{F}\text{×}\text{n}_{\text{DMC}\text{/}\text{DEC}}}{\text{Q}}$ (2)^[1]^

$\text{n}_{\text{DMC}\text{/}\text{DEC}}$: the moles of DMC, mol;

$\text{Q}$: the total charge passed, C.

***Economic benefits of the electrocatalyst was calculated by the equation:***

$\text{SEC}\text{=}\frac{\text{V}_{\text{cell}}\text{·}\text{n}_{\text{e}^{\text{−}}}\text{·96485}}{\text{FE}_{\text{DMC}}\text{·}\text{M}_{\text{DMC}}\text{·3600}}$ (3)^[2]^

$\text{SEC}$: Electric energy consumption per unit product, kWh·kg^−1^;

$\text{V}_{\text{cell}}$: Operating voltage of the full electrolytic cell, 4.2 V;

$\text{n}_{\text{e}^{\text{−}}}$: Cathodic charge transfer amount, 2 e^−^;

$\text{M}_{\text{DMC}}$: the relative molecular mass of DMC, 90.08 g/mol.

***Product Calculations:***

The gas products of electrolysis were continuously plunged into the gas sampling loop equipped with a thermal conductivity detector (TCD), a flame ionization detector (FID), and a capillary column. High purity argon (99.9999%) was used as the carrier gas for the gas chromatography. The quantification for each gas was determined by external standard method. All data were taken as the average of three tests.Figure S1 shows the CO standard curve.

The liquid phase product from electrolysis was continuously injected into the sampling loop of a gas chromatograph equipped with a TCD, a FID, and a capillary column. High purity argon (99.9999%) was used as the carrier gas. Quantitative analysis of the liquid phase products was determined by the internal standard method, and all data were taken as the average of three tests. Figure S2 shows the DMC standard curve, and Figure S3 shows the DEC standard curve (with n-propanol as the internal standard).

***Statistical Analysis***

All quantitative data are reported as the mean of three independent experiments (n=3). Error bars in figures represent the standard deviation (SD). Statistical comparisons were performed using a two-tailed Student’s t-test or one-way ANOVA with Tukey’s test, as appropriate. A p-value < 0.05 was considered significant. Data were processed using OriginPro 2021.


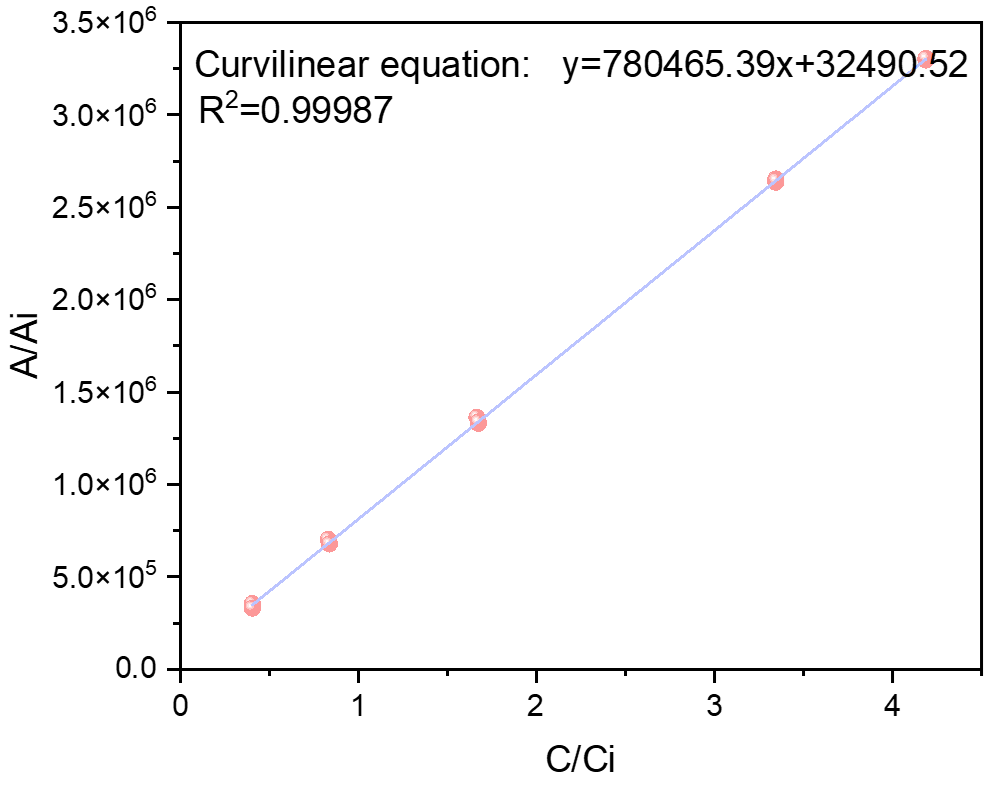


**Figure S1. CO standard curve.**


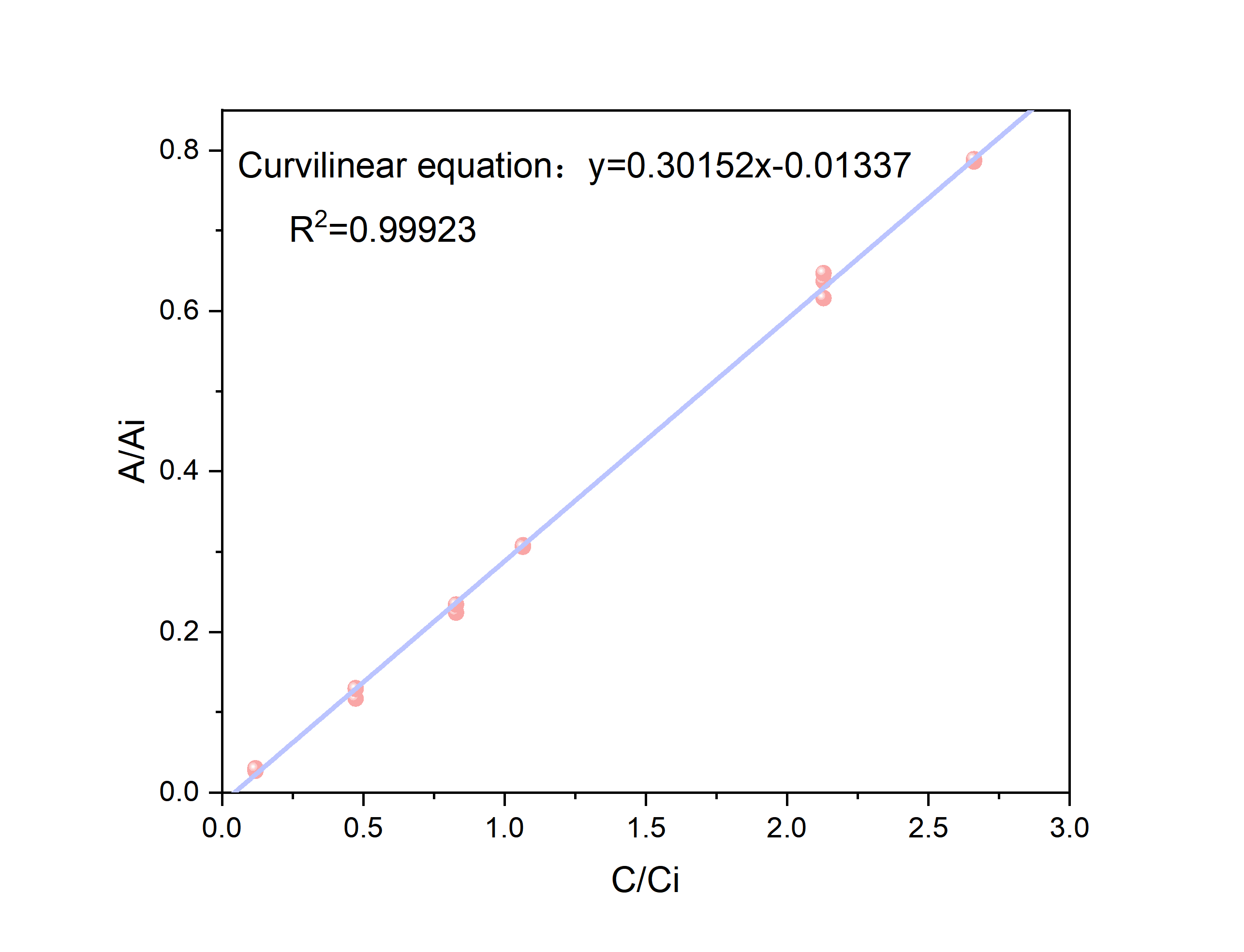


**Figure S2. DMC standard curve.**


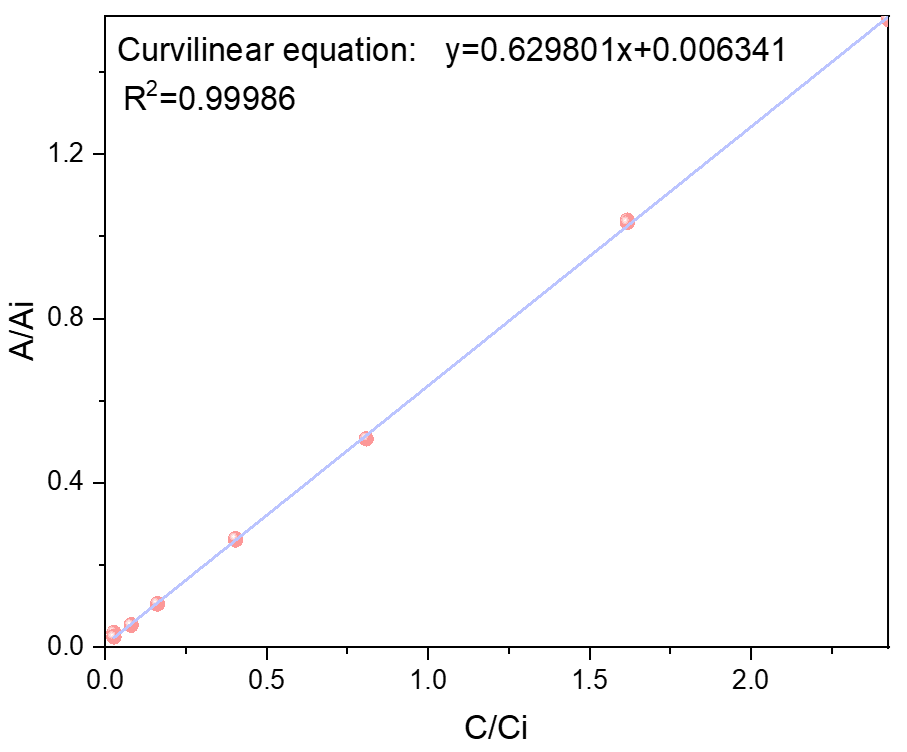


**Figure S3. DEC standard curve.**

1. **Figures and Tables**

**
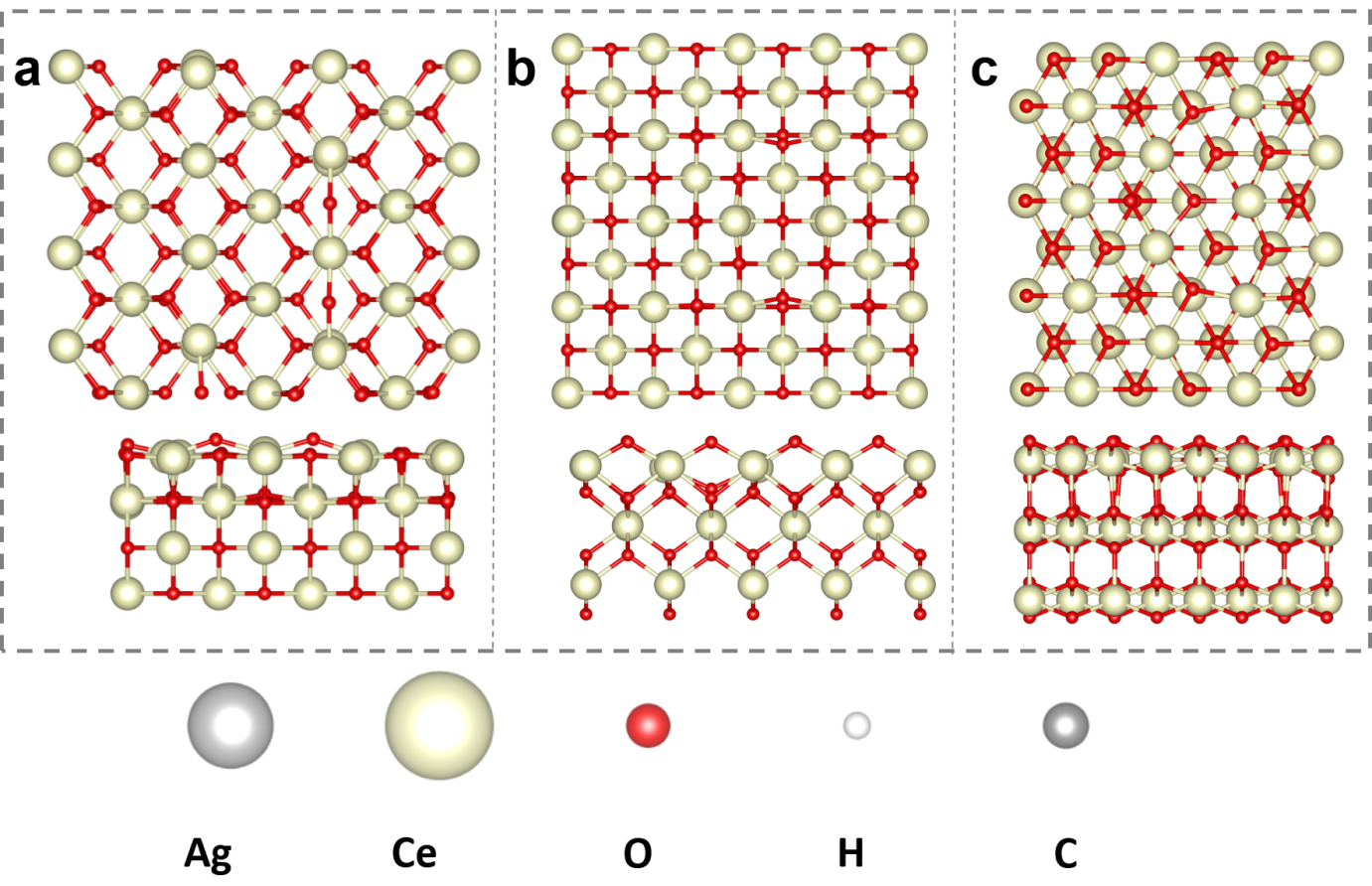
**

**Figure S4. DFT-Optimized Adsorption Configurations of Different Morphologies (a: R-CeO_2_; b: C-CeO_2_ and c: G-CeO_2_).**


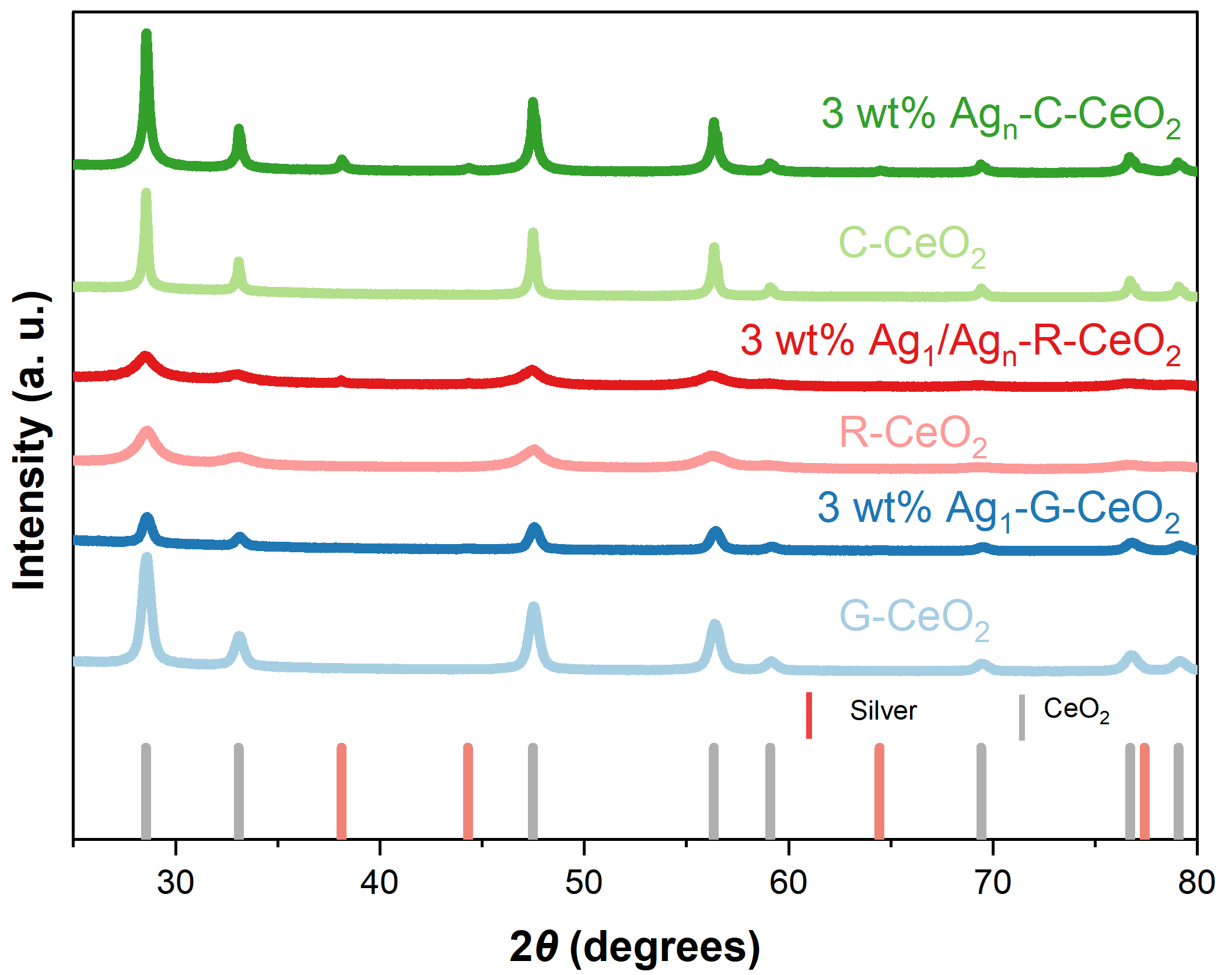


**Figure S5. XRD patterns of CeO_2_, silver-modified CeO_2_ catalysts.**

***
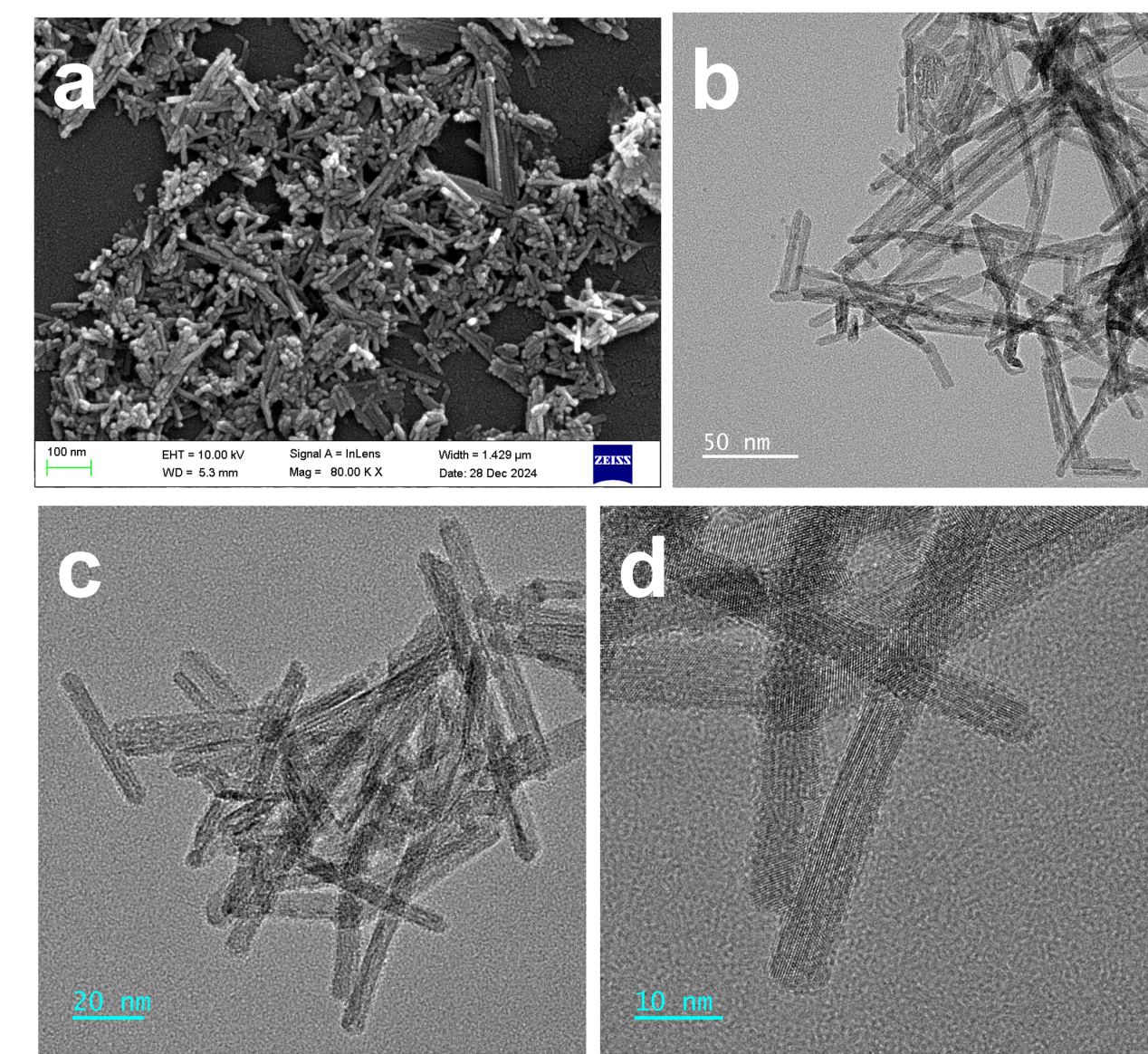
***

**Figure S6. a: SEM image of Ag_1_/Ag_n_-R-CeO_2_; b-d: TEM image of Ag_1_/Ag_n_-R-CeO_2_.**


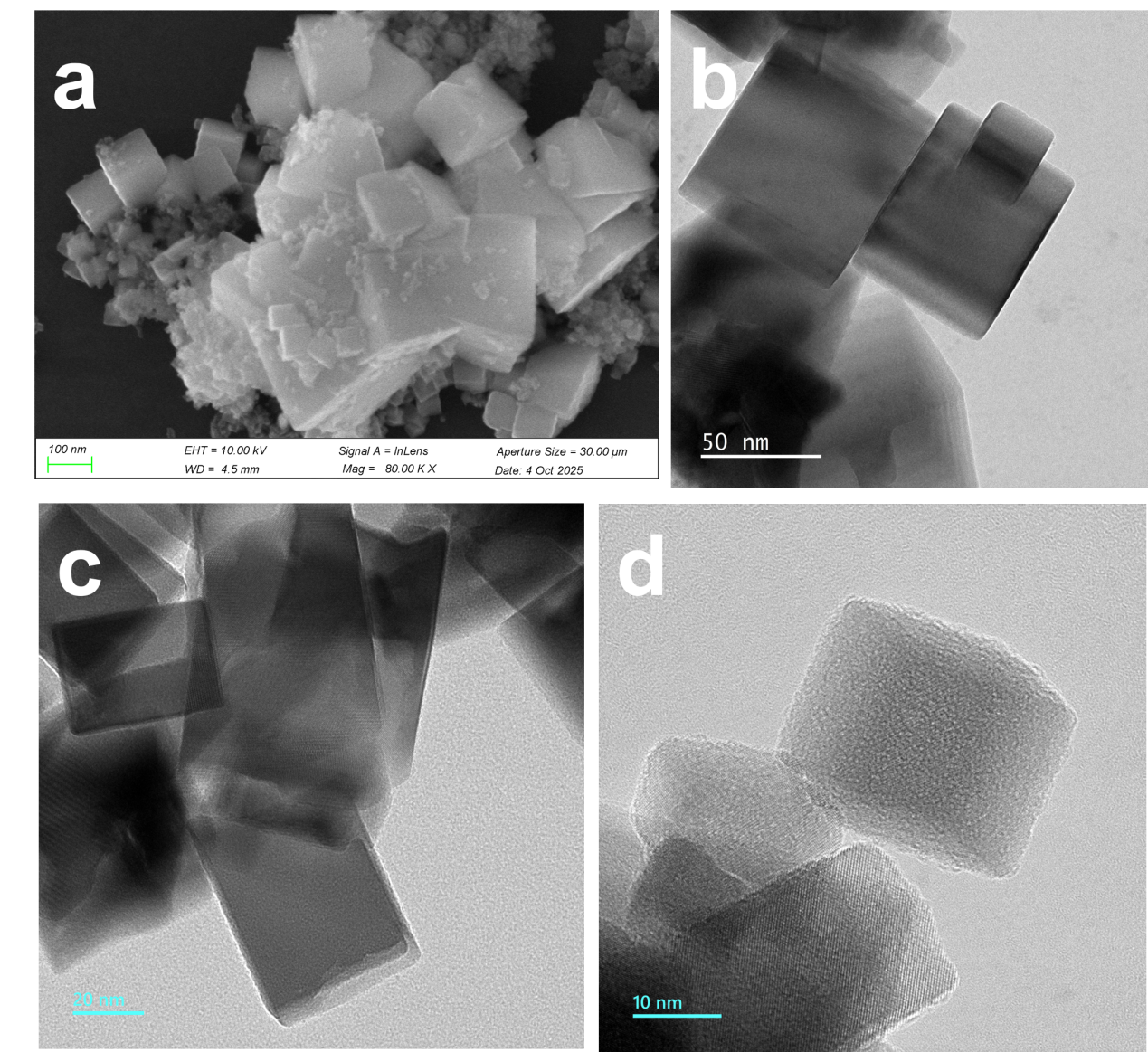


**Figure S7. a: SEM image of Ag_n_-C-CeO_2_; b-d: TEM image of Ag_n_-C-CeO_2_.**


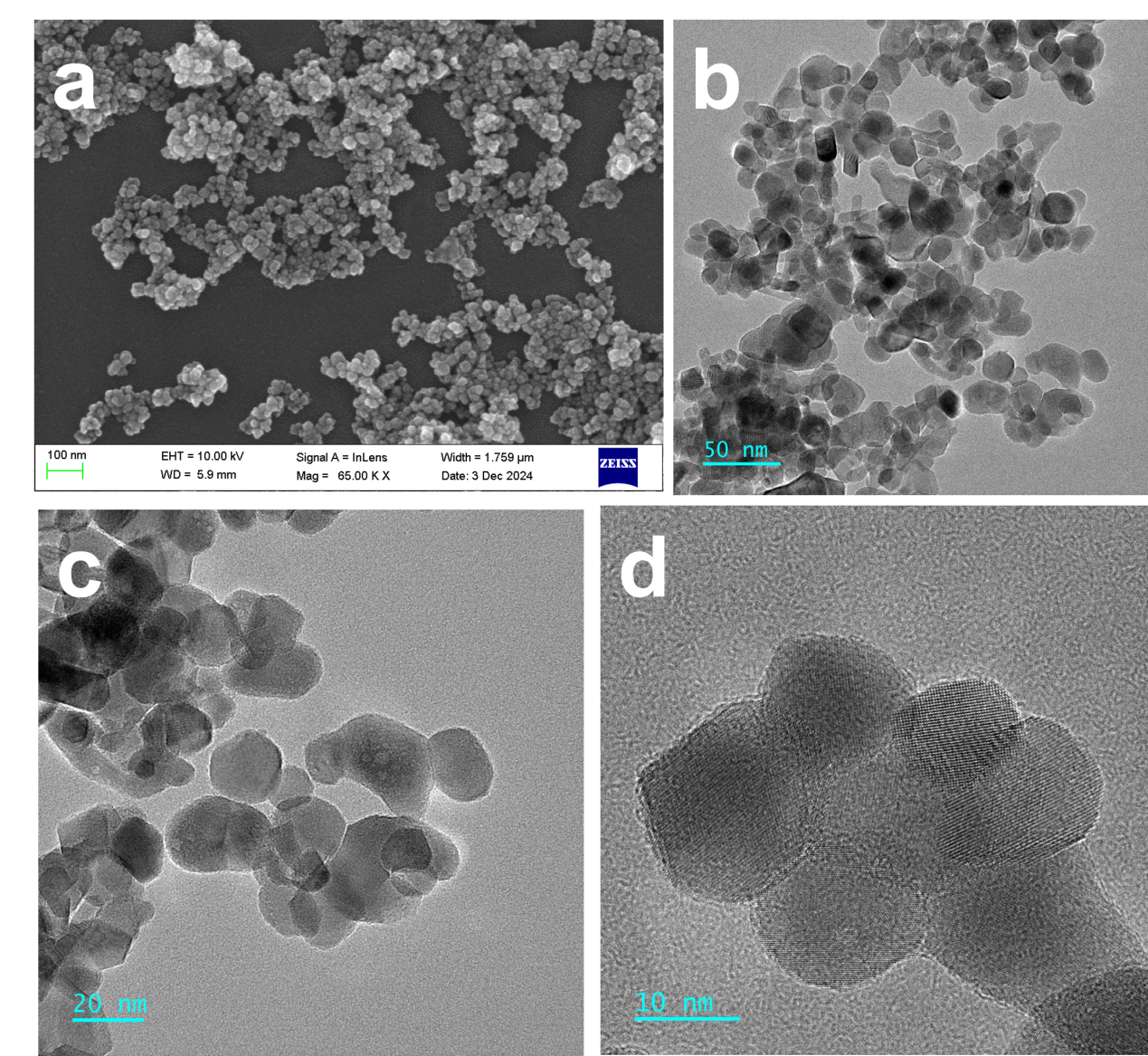


**Figure S8. a: SEM image of Ag_1_-G-CeO_2_; b-d: TEM image of Ag_1_-G-CeO_2_.**

***
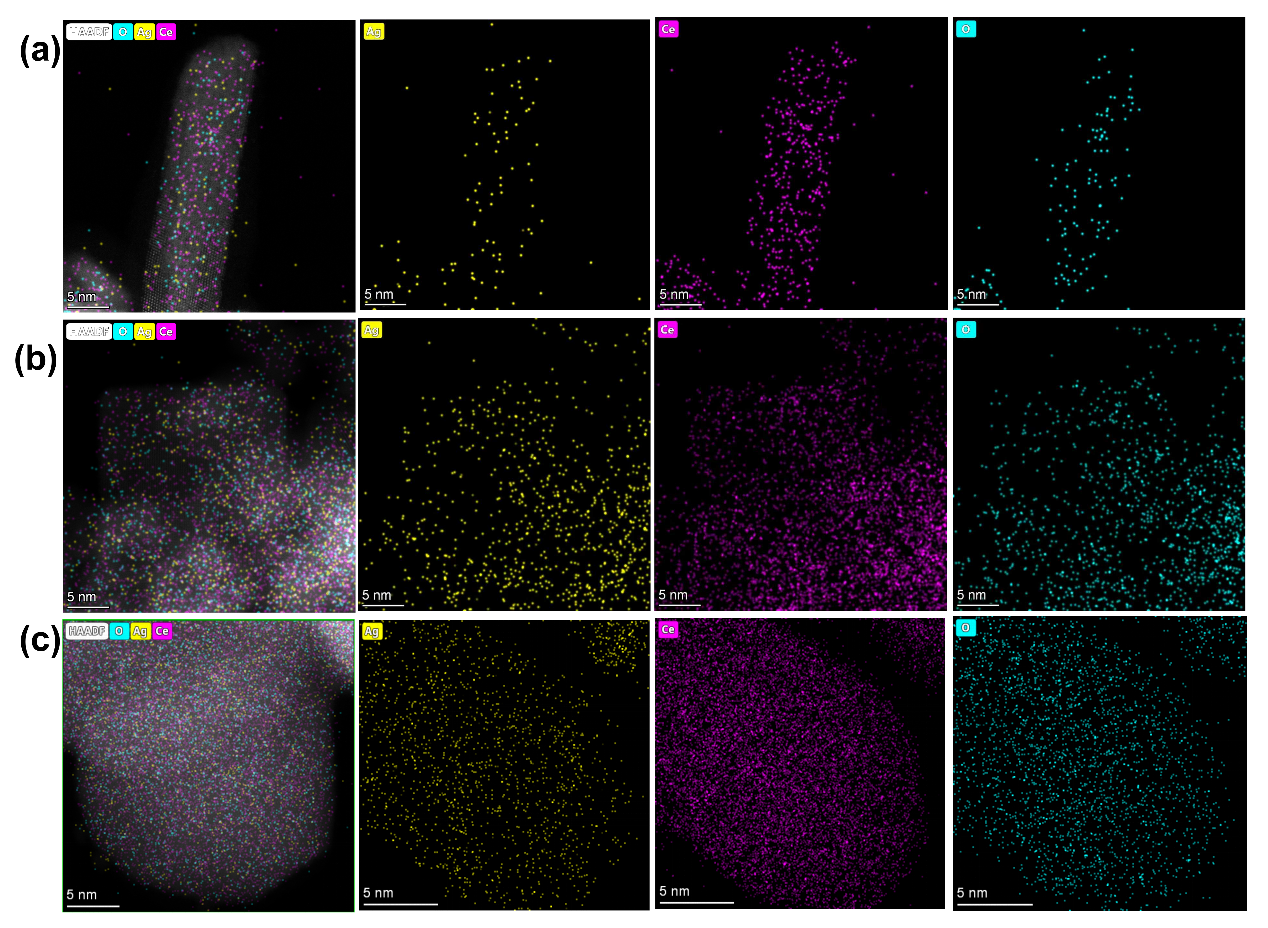
***

**Figure S9. a: EDS Mapping of Ag_1_/Ag_n_-R-CeO_2_; b: EDS Mapping of Ag_n_-C-CeO_2_ and c: EDS Mapping of Ag_1_-G-CeO_2_.**


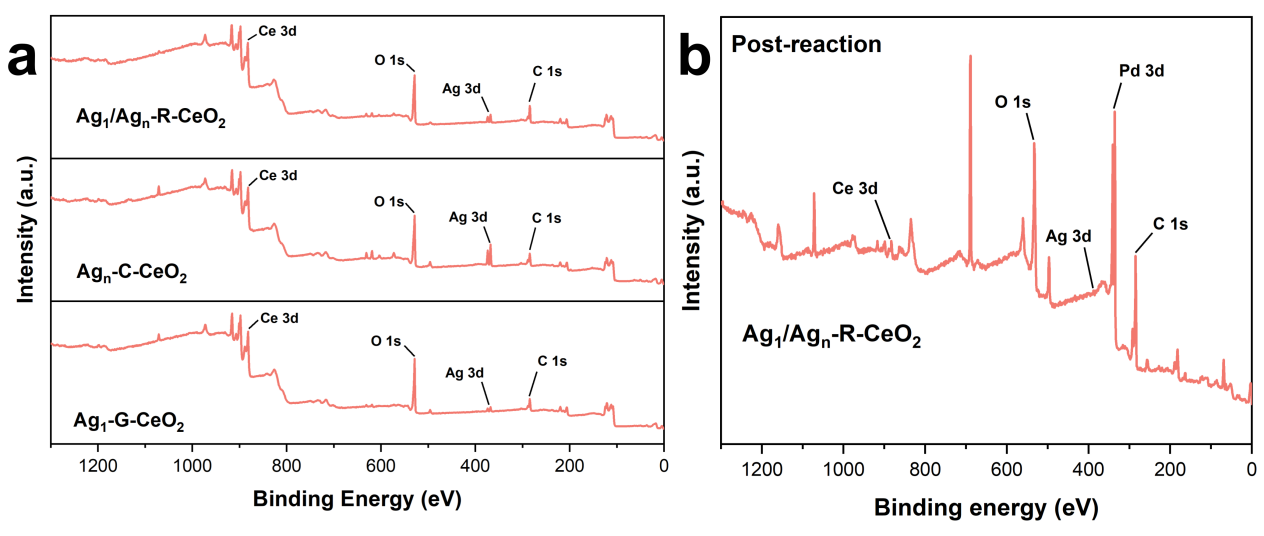


**Figure S10. a: XPS survey spectrum of Ag_1_/Ag_n_-R-CeO_2_, Ag_n_-C-CeO_2_ and Ag_1_-G-CeO_2_ Pre-reaction; b: XPS survey spectrum of Ag_1_/Ag_n_-R-CeO_2_ after 20 cycles of reaction.**

**
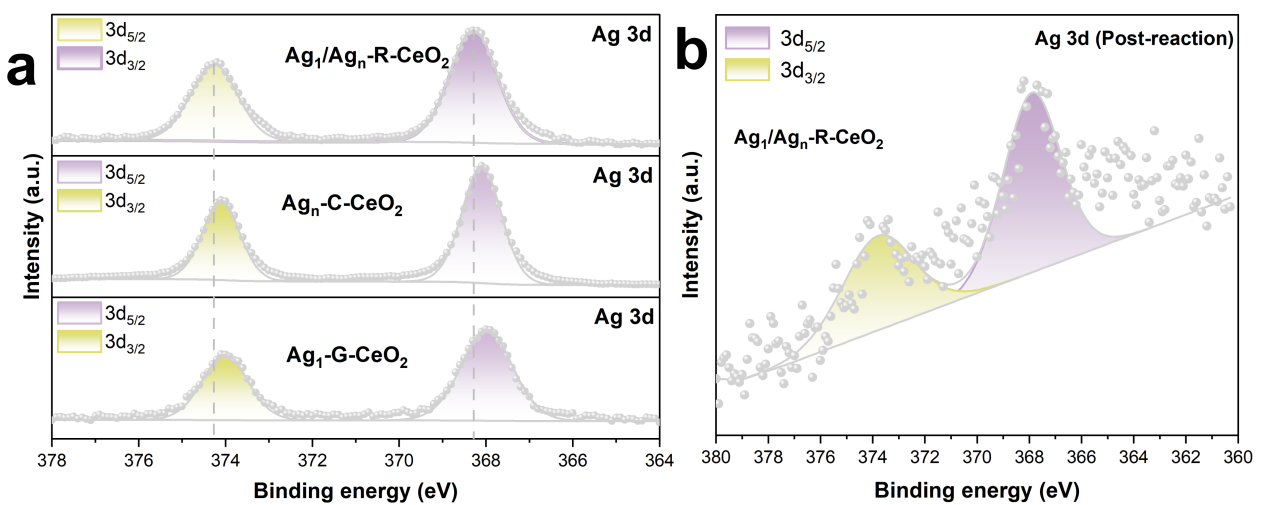
**

**Figure S11. a: XPS spectra for Ag 3d (Ag_1_/Ag_n_-R-CeO_2_, Ag_n_-C-CeO_2_ and Ag_1_-G-CeO_2_) Pre-reaction; b: XPS spectra for Ag 3d (Ag_1_/Ag_n_-R-CeO_2_) after 20 cycles of reaction.**


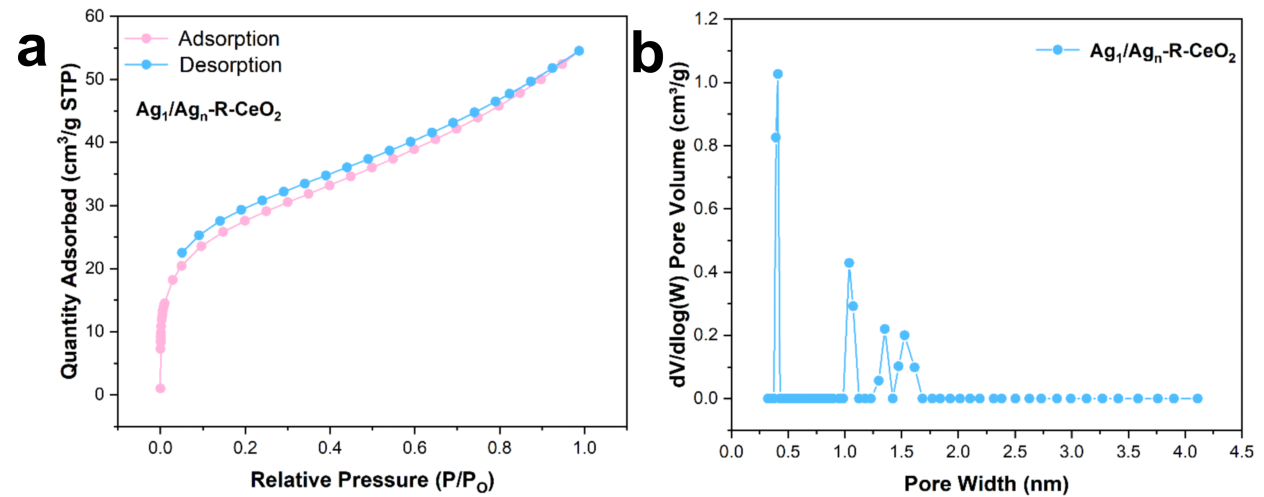


**Figure S12. a CO_2_ adsorption–desorption isotherms of Ag_1_/Ag_n_-R-CeO_2_; b: Pore size distribution of Ag_1_/Ag_n_-R-CeO_2_.**


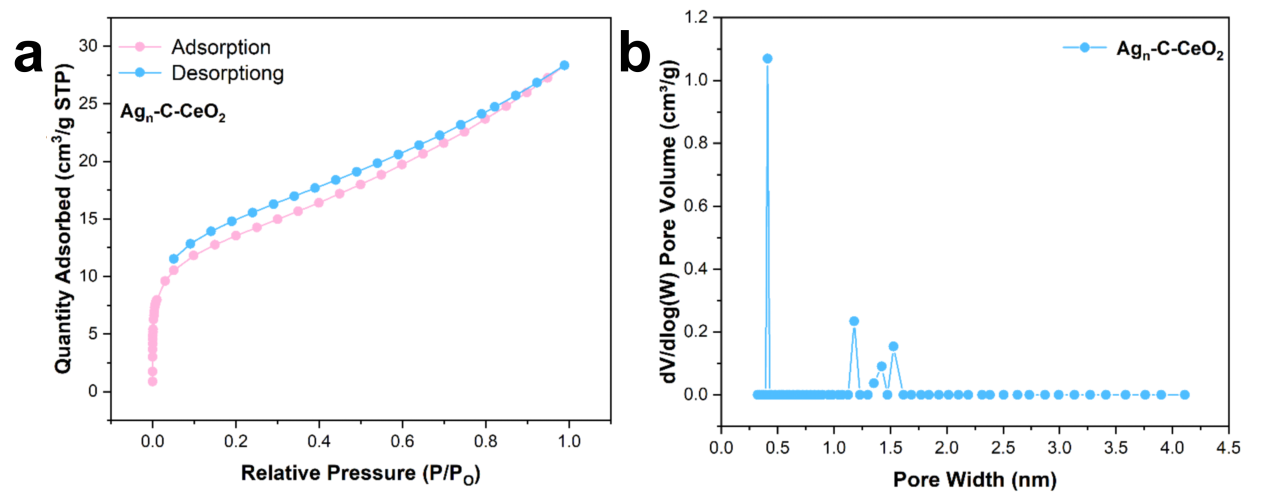


**Figure S13. a: CO_2_ adsorption–desorption isotherms of Ag_n_-C-CeO_2_; b: Pore size distribution of Ag_n_-C-CeO_2_.**


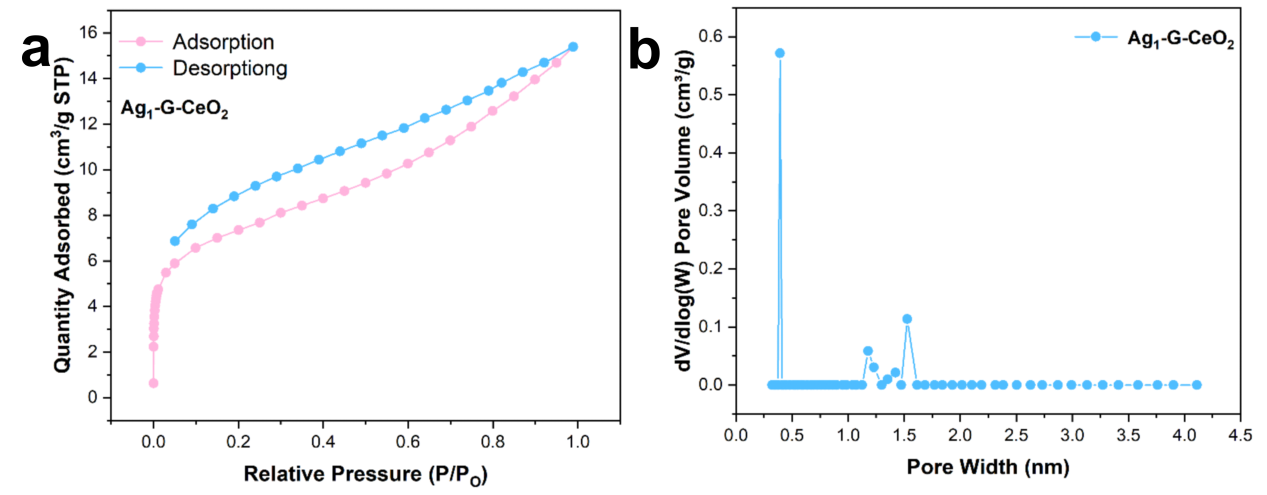


**Figure S14. a: CO_2_ adsorption–desorption isotherms of Ag_1_-G-CeO_2_; b: Pore size distribution of Ag_1_-G-CeO_2_.**


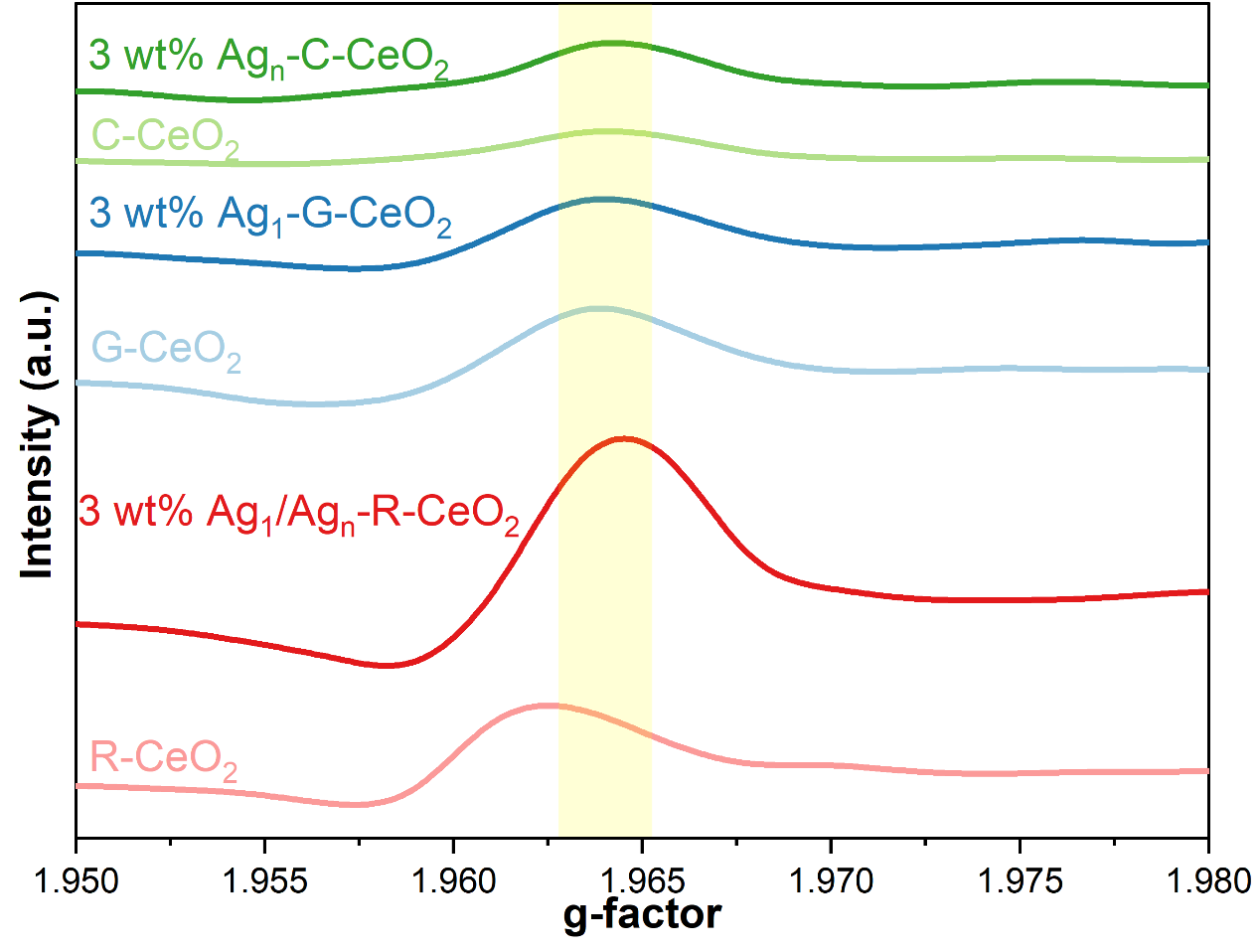


**Figure S15. EPR Spectra Illustrating Ce^3+^ Concentrations.**

**
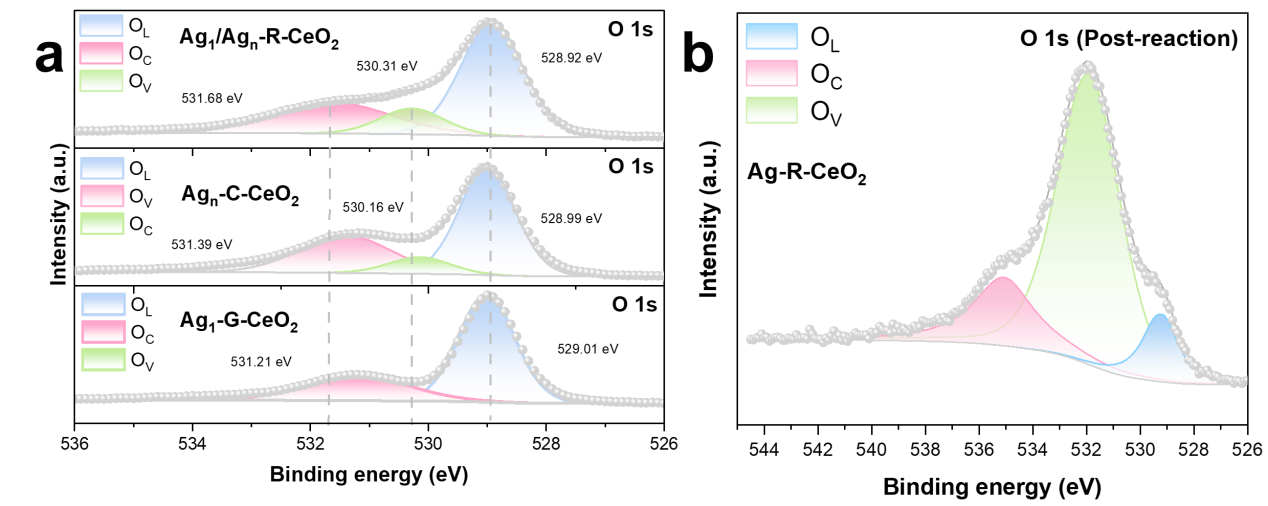
**

**Figure S16. a: XPS spectra for O 1s (Ag_1_/Ag_n_-R-CeO_2_, Ag_n_-C-CeO_2_ and Ag_1_-G-CeO_2_) Pre-reaction; b: XPS spectra for O 1s (Ag_1_/Ag_n_-R-CeO_2_) after 20 cycles of reaction.**

**
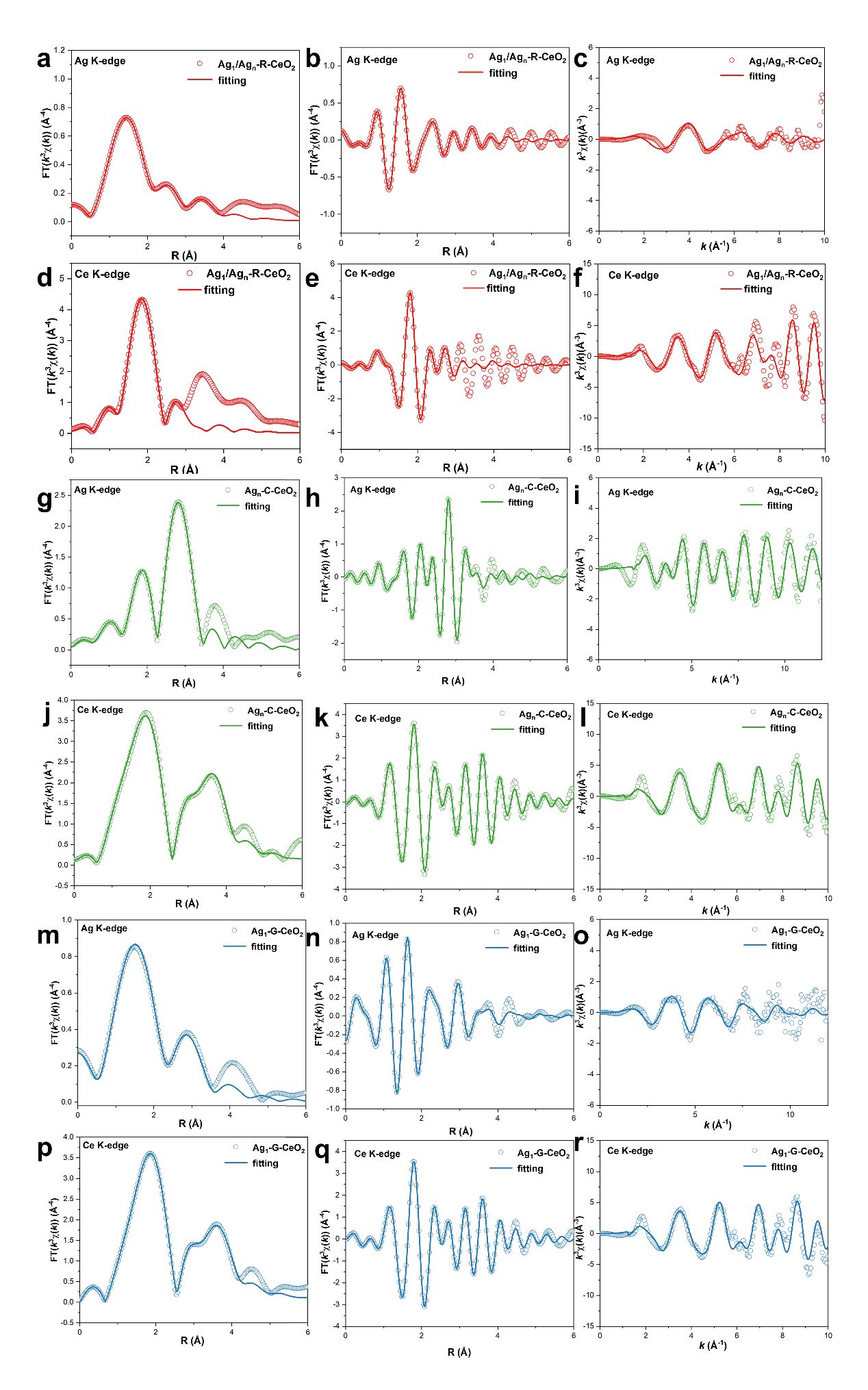
**

**Figure S17. Ag K-edge and Ce K-edge EXAFS Spectra (R-space curves, Rmr, k^3^-weighted χ(k) curves) of three catalysts, with subplots grouped as:**

**(a–f) Ag_1_/Ag_n_-R-CeO_2_: (a–c) Ag K-edge, (d–f) Ce K-edge**

**(g–l) Ag_n_-C-CeO_2_: (g–i) Ag K-edge, (j–l) Ce K-edge**

**(m–r) Ag_1_-G-CeO_2_: (m–o) Ag K-edge, (n–r) Ce K-edge**

**For each catalyst, the three subplot columns correspond to: R-space curve, Rmr (radial distribution curve after multiple scattering correction), and k^3^-weighted χ(k) curve (k-space).**


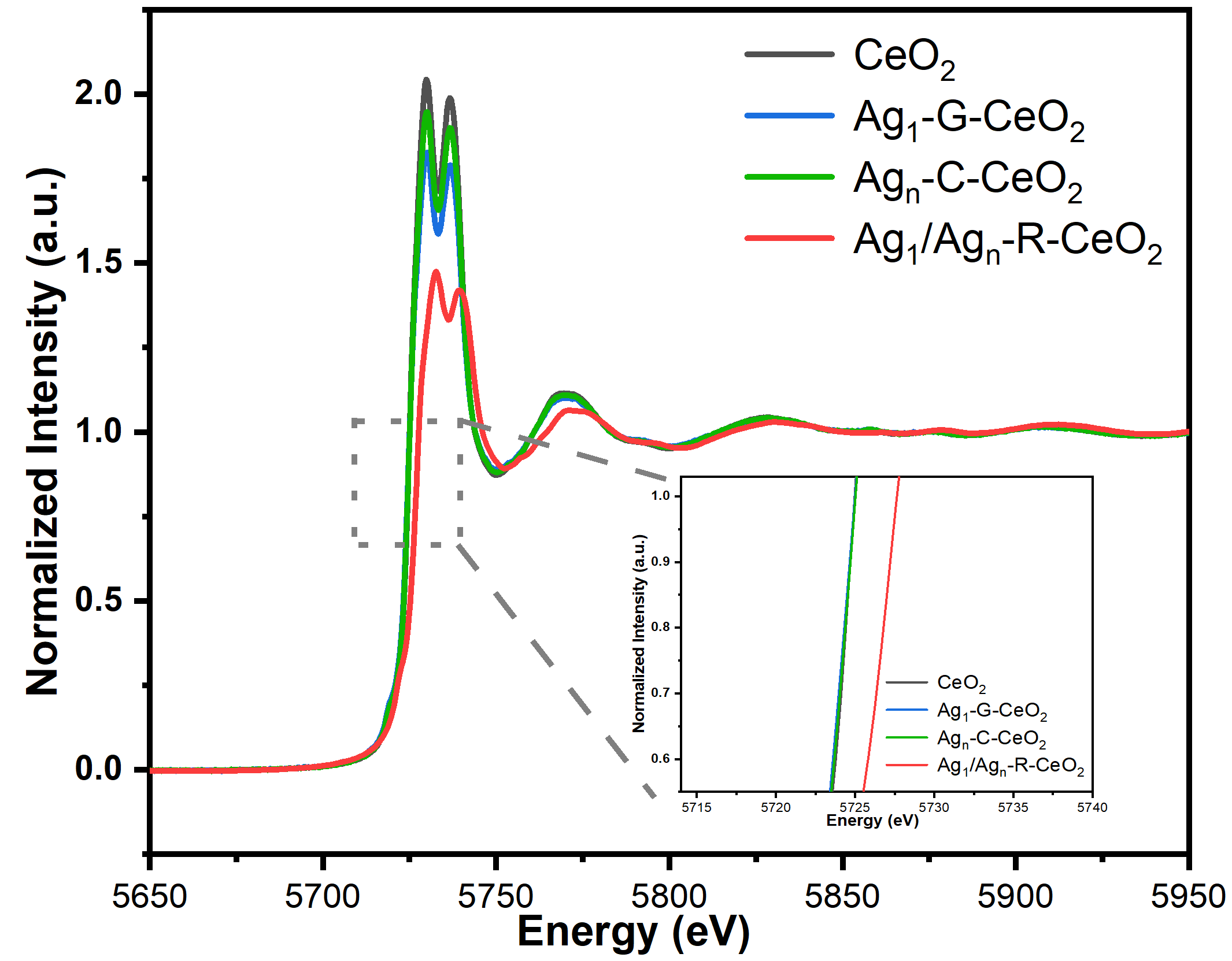


**Figure S18. Pre-reaction Ce K-edge XANES Spectra.**


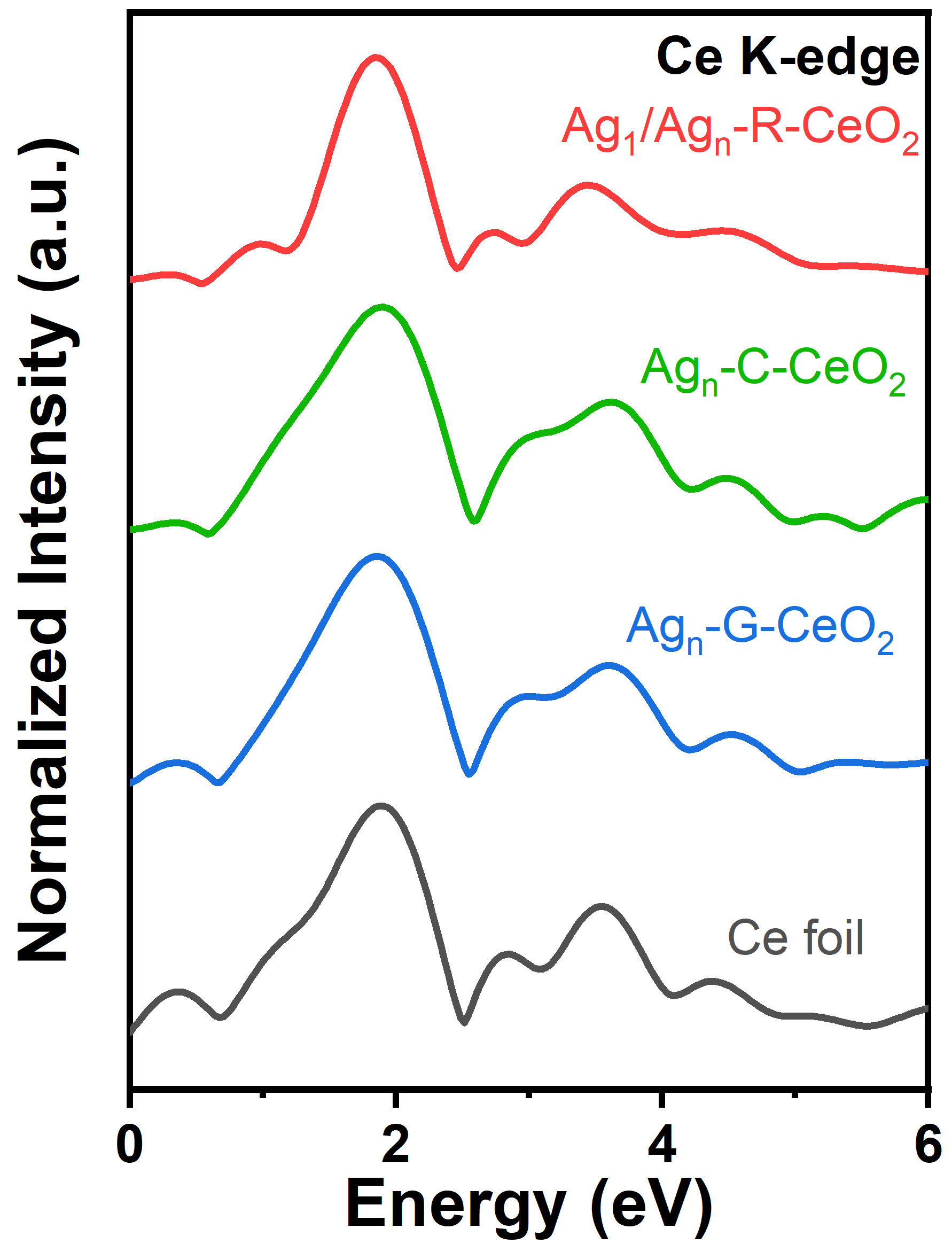


**Figure S19. Pre-reaction Ce K-edge EXAFS Spectra Depicting the Local Coordination Environments of Ce.**

**Figure S20. Pre-reaction WT-EXAFS spectra of Ce species (Ag_1_/Ag_n_-R-CeO_2_).**


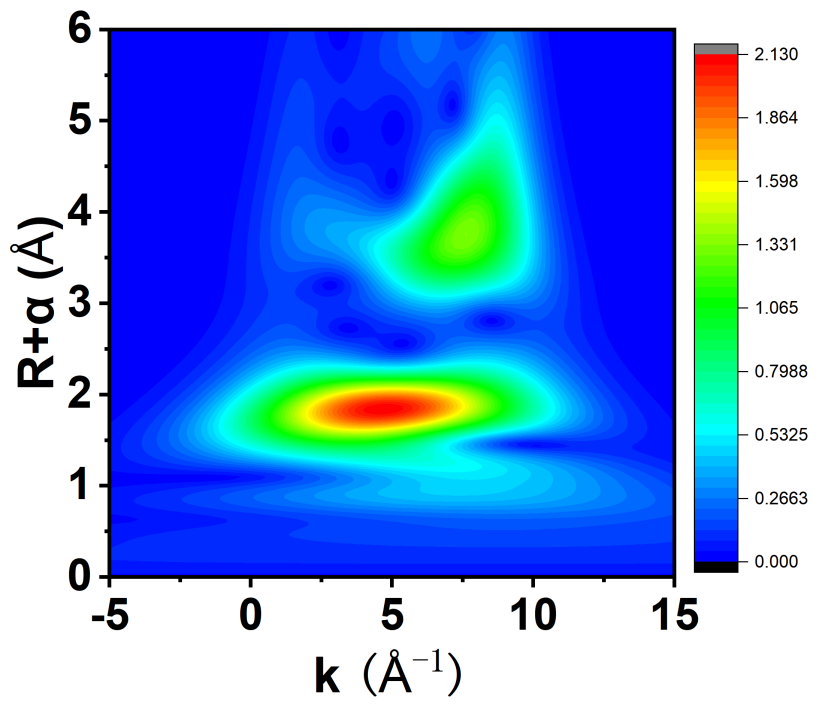


**Figure S21. Pre-reaction WT-EXAFS spectra of Ce species (Ag_n_-C-CeO_2_).**


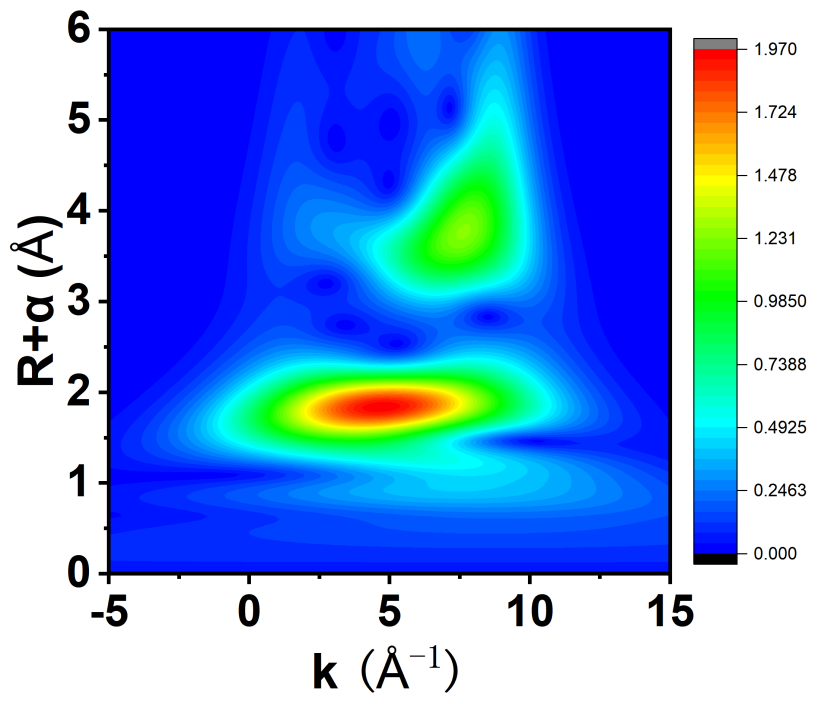


**Figure S22. Pre-reaction WT-EXAFS spectra of Ce species (Ag_1_-G-CeO_2_).**

**
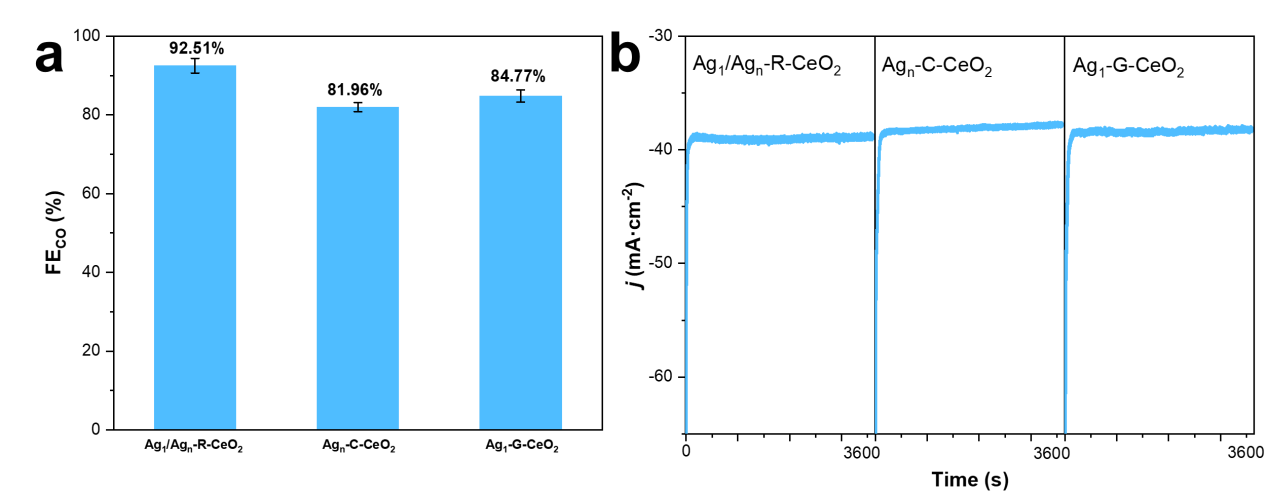
**

**Figure S23. a: FE_CO_ of Ag_1_/Ag_n_-R-CeO_2_, Ag_n_-C-CeO_2_ and Ag_1_-G-CeO_2_; b: Amperometric i-t curve of Ag_1_/Ag_n_-R-CeO_2_, Ag_n_-C-CeO_2_ and Ag_1_-G-CeO_2_. Data for FE and product concentration represent the mean values of three independent measurements**. **Error bars indicate the standard deviation.**


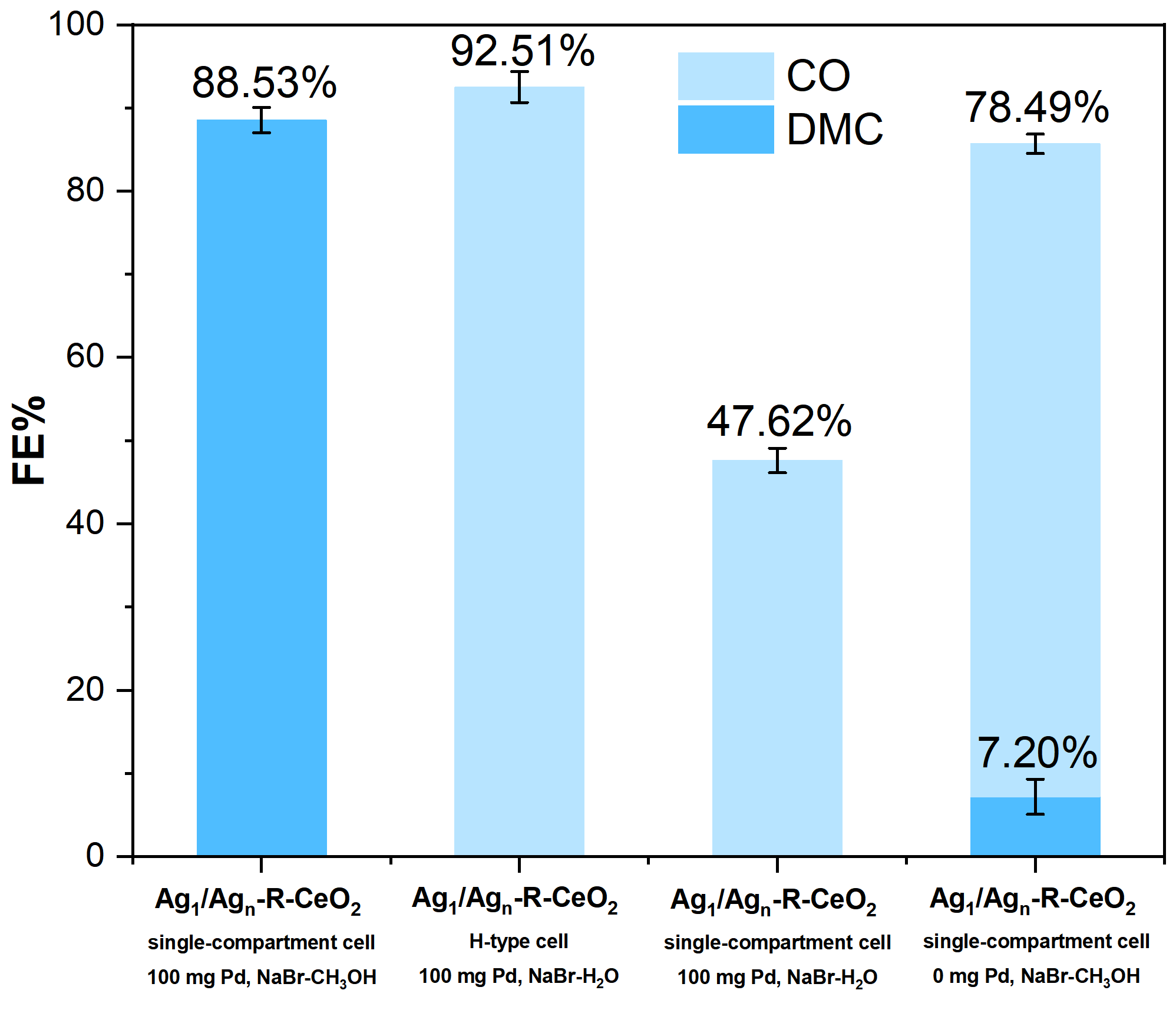


**Figure S24. FE of CO and DMC over Ag_1_/Ag_n_-R-CeO_2_ catalyst under different cell configurations, electrolytes, and Pd loadings.Data for FE and product concentration represent the mean values of three independent measurements**. **Error bars indicate the standard deviation.**


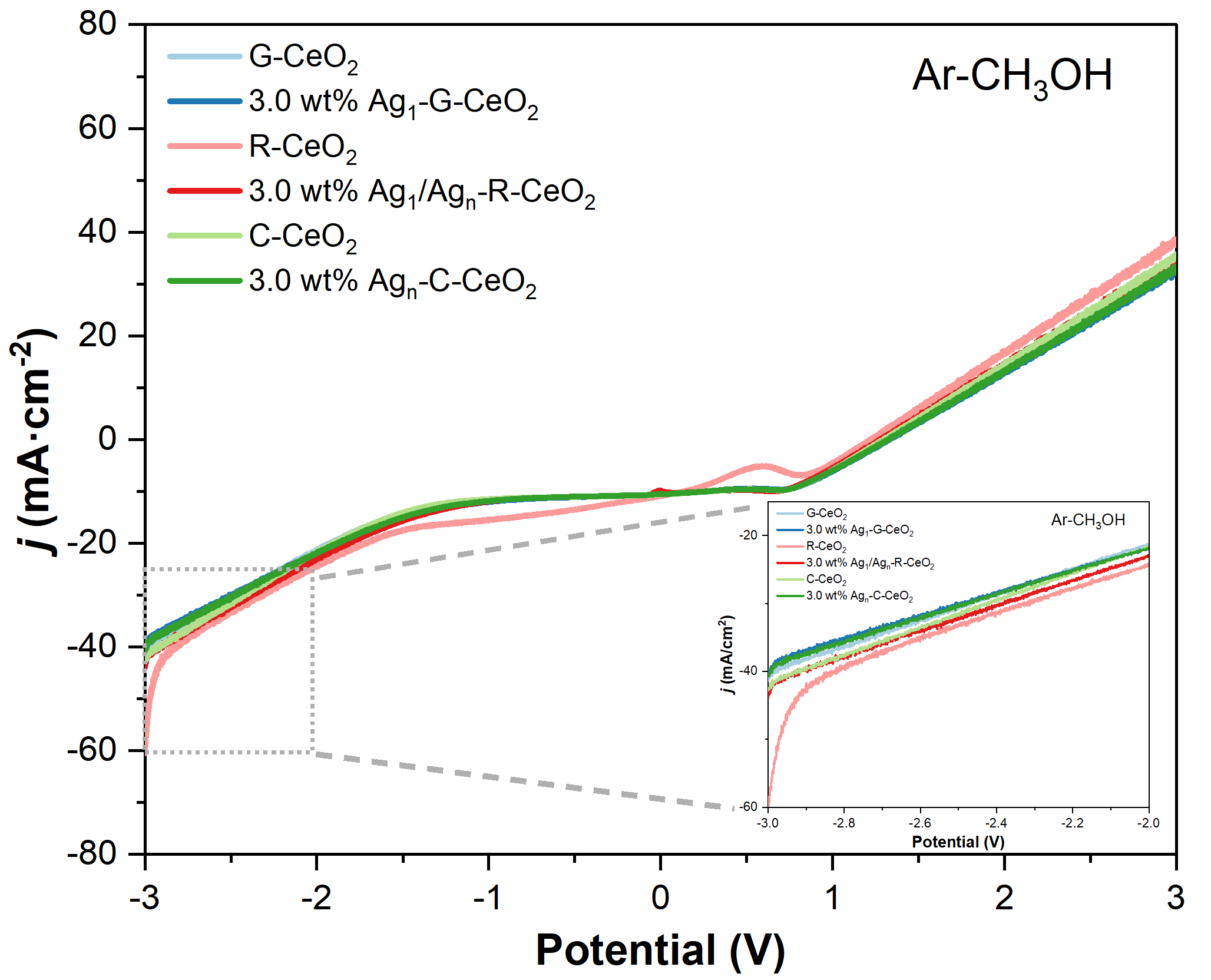


**Figure S25. Ar-saturated LSV curves.**


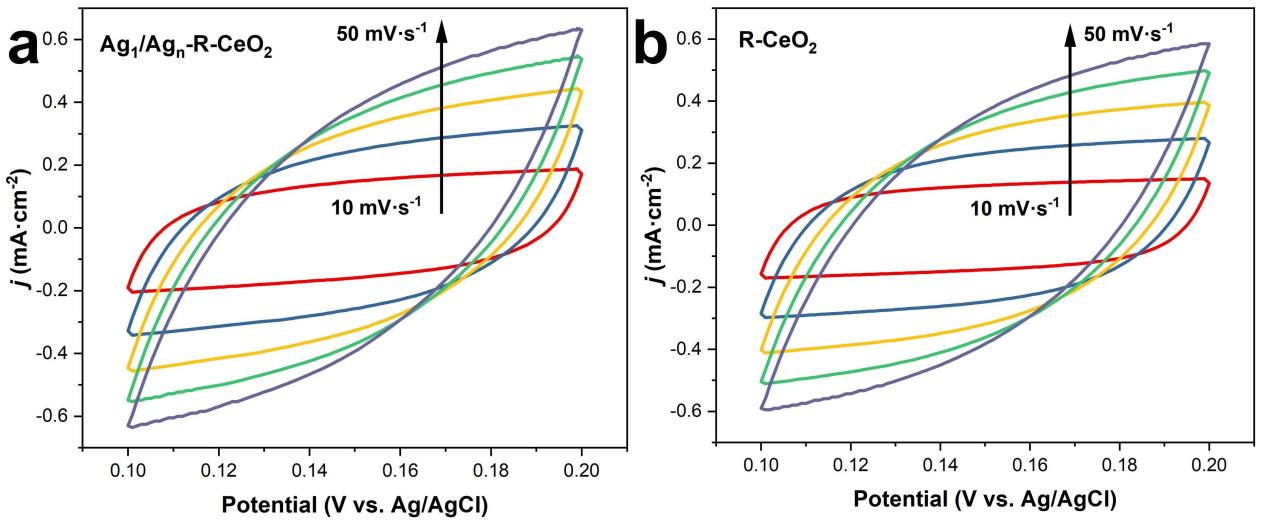


**Figure S26. a: current density difference and scan rate of Ag_1_/Ag_n_-R-CeO_2_; b: current density difference and scan rate of R-CeO_2_.**


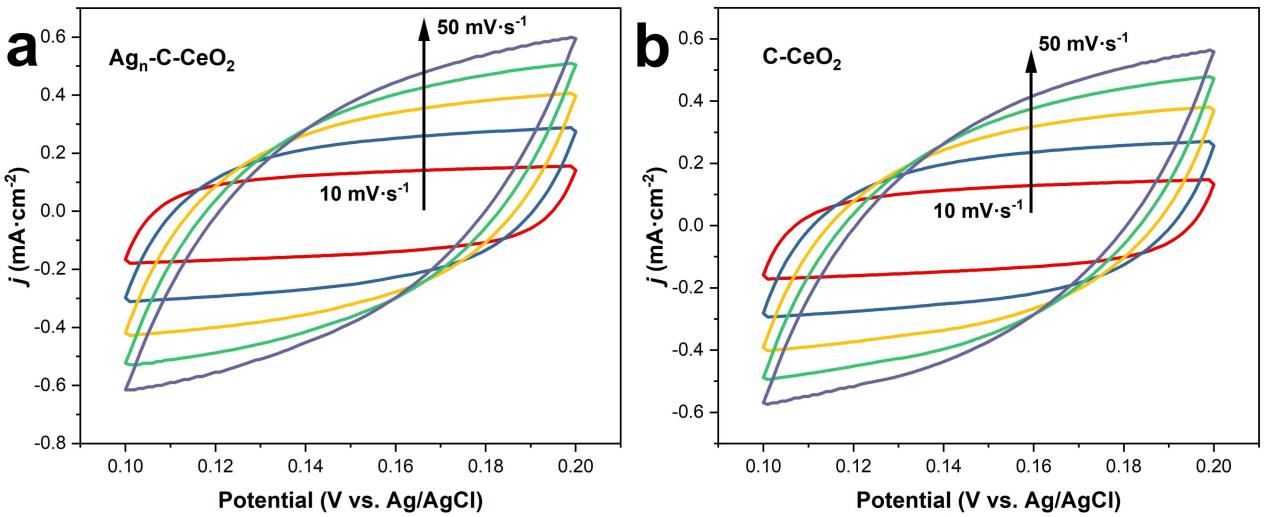


**Figure S27. a: current density difference and scan rate of Ag_n_-C-CeO_2_; b: current density difference and scan rate of C-CeO_2_.**


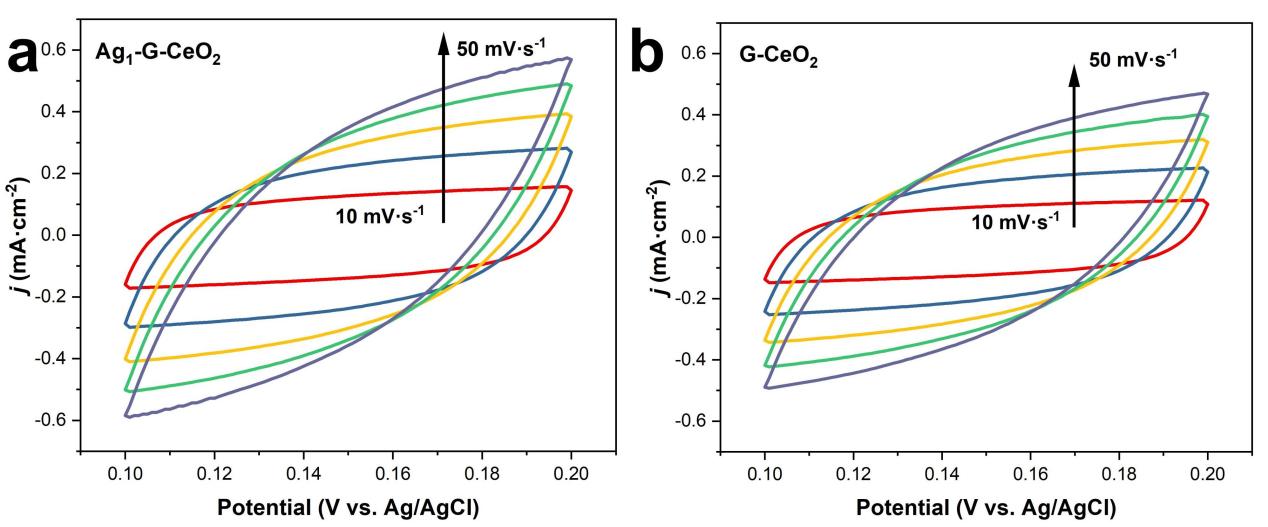


**Figure S28. a: current density difference and scan rate of Ag_1_-G-CeO_2_; b: current density difference and scan rate of G-CeO_2_.**

**
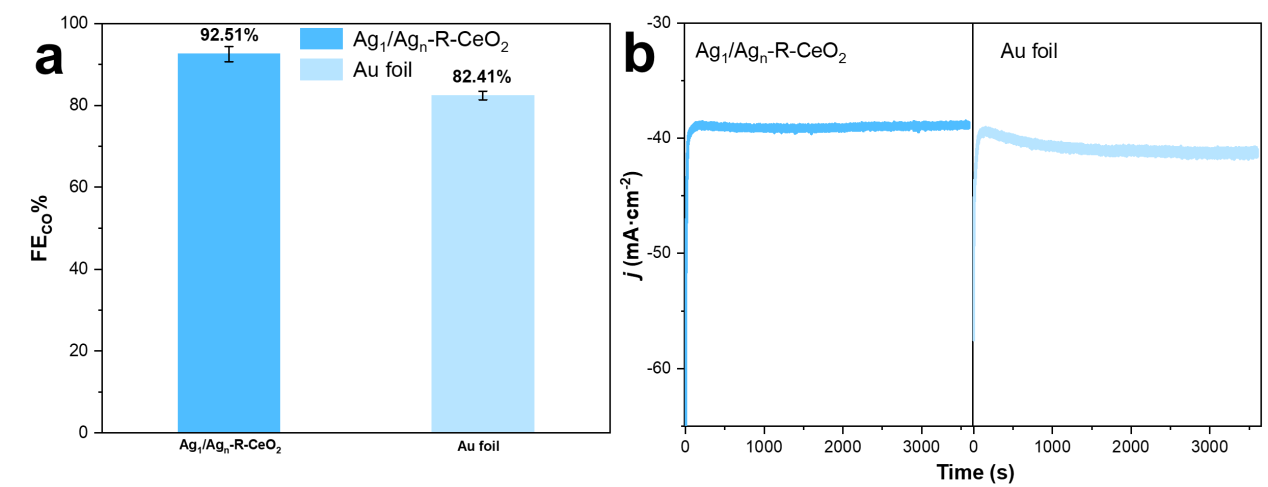
**

**Figure S29. a: FE_CO_ of Ag_1_/Ag_n_-R-CeO_2_ and Au foil; b: Amperometric i-t curve of Ag_1_/Ag_n_-R-CeO_2_ and Au foil. Data for FE and product concentration represent the mean values of three independent measurements**. **Error bars indicate the standard deviation.**


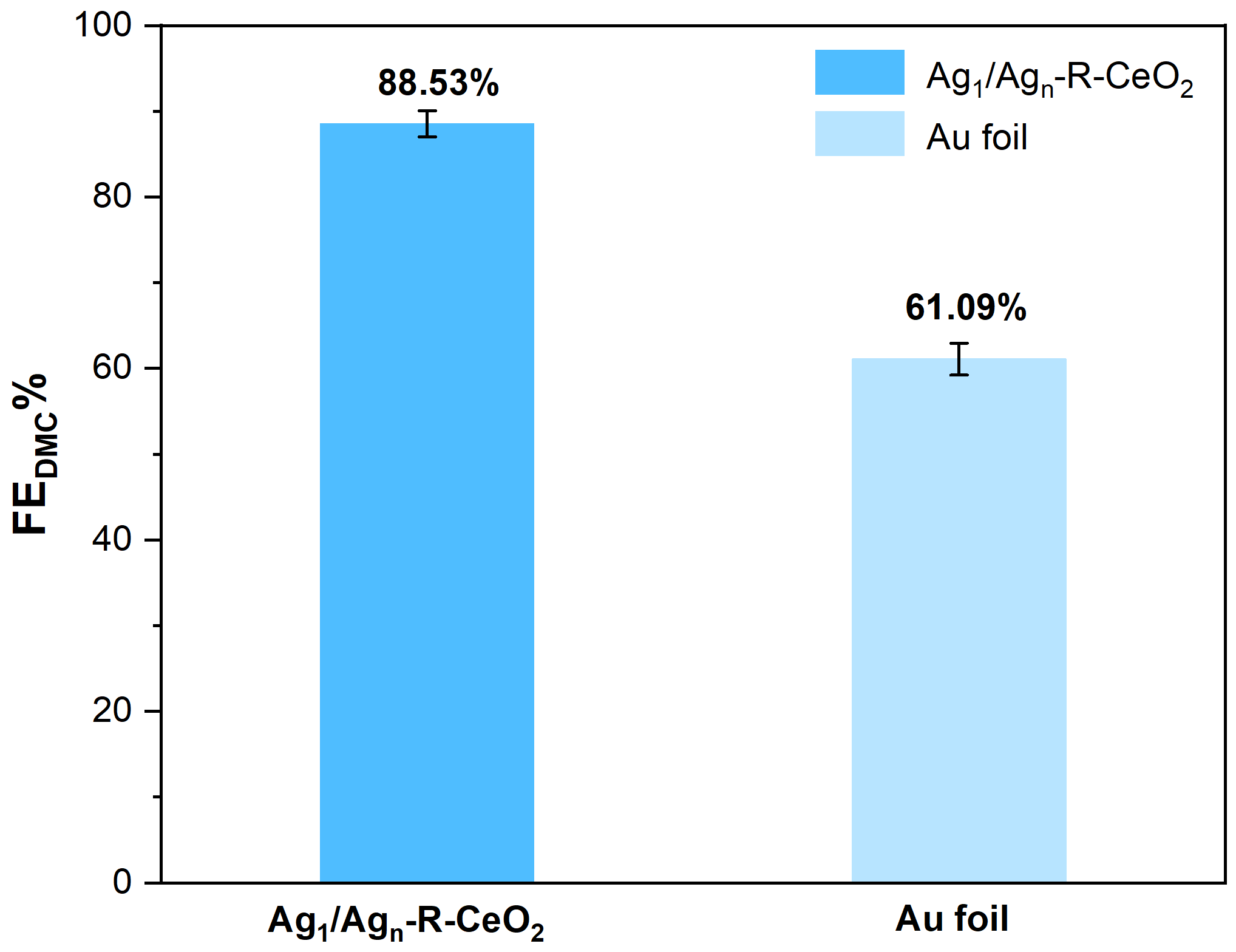


**Figure S30. FE_DMC_ of Ag_1_/Ag_n_-R-CeO_2_ and Au foil. Data for FE and product concentration represent the mean values of three independent measurements**. **Error bars indicate the standard deviation.**


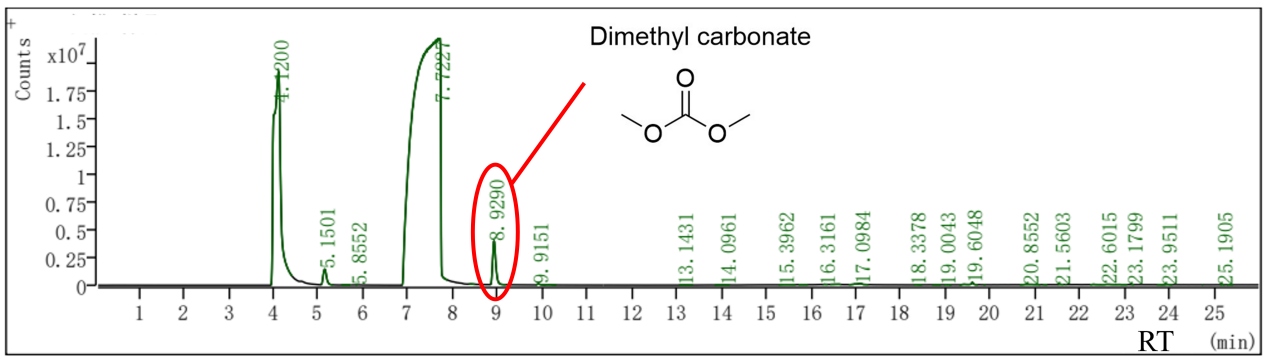


**Figure S31. GC-MS spectrum of DMC in post-reaction solution.**

**
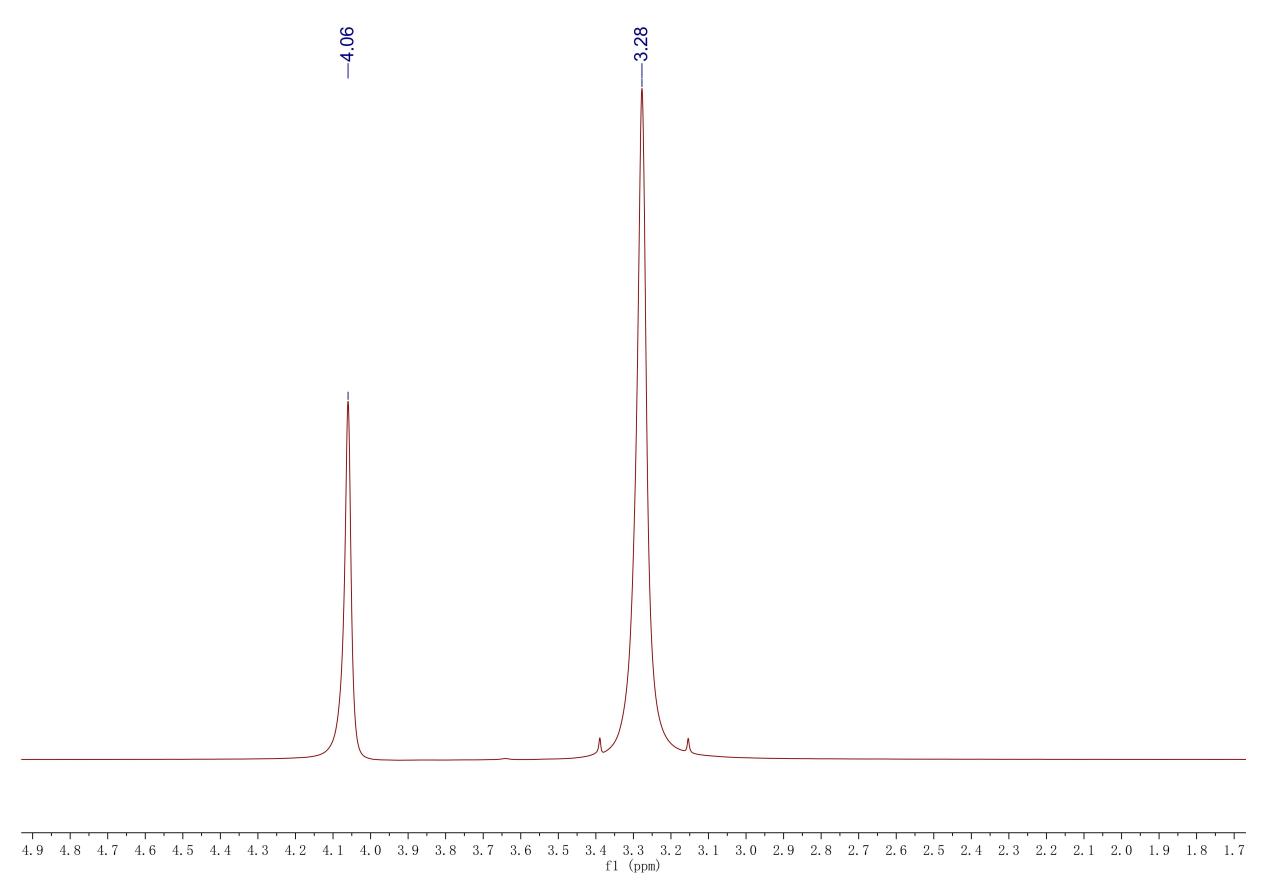
**

**Figure S32. ^1^H NMR (Pre-reaction).**

**
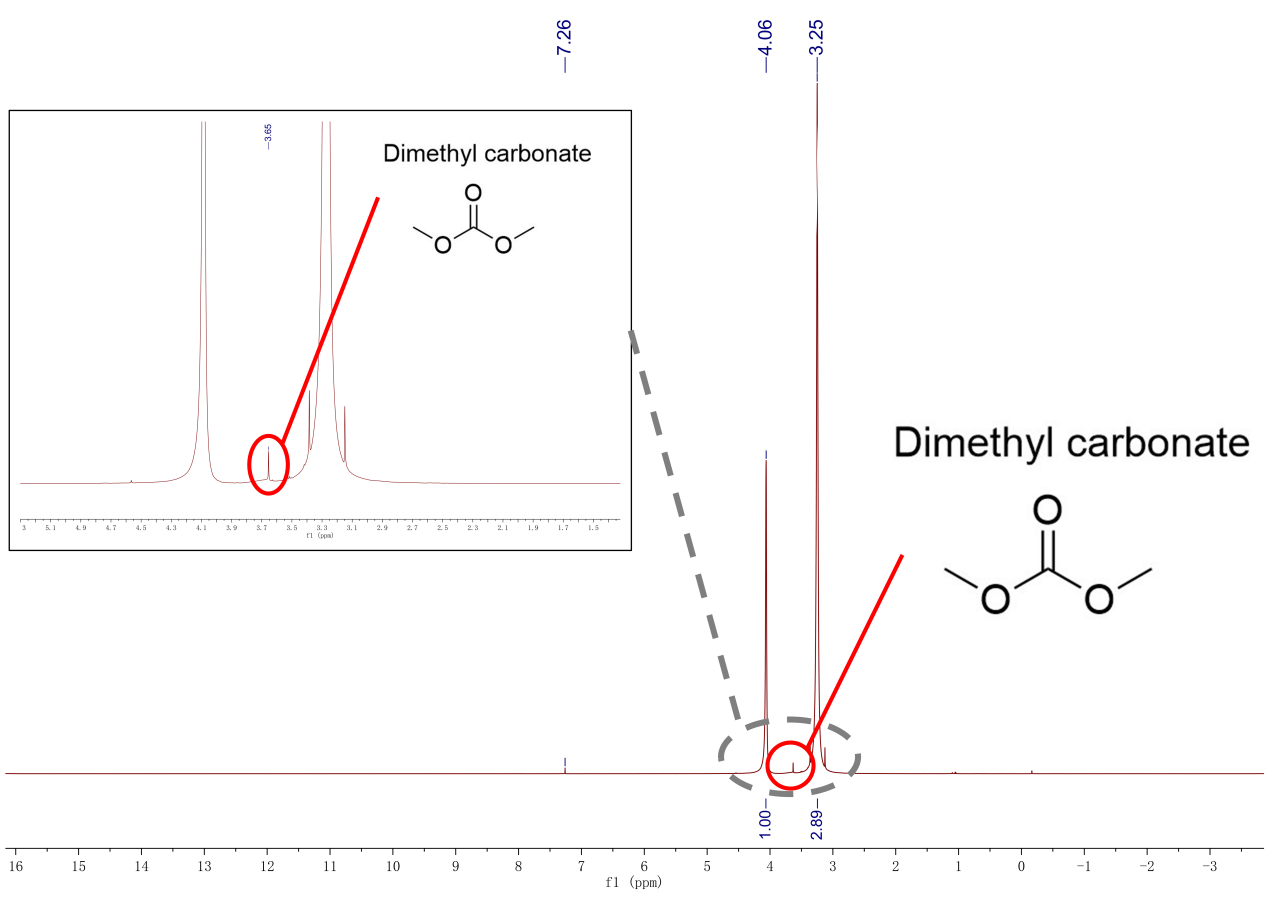
**

**Figure S33. ^1^H NMR (Post-reaction).**

**
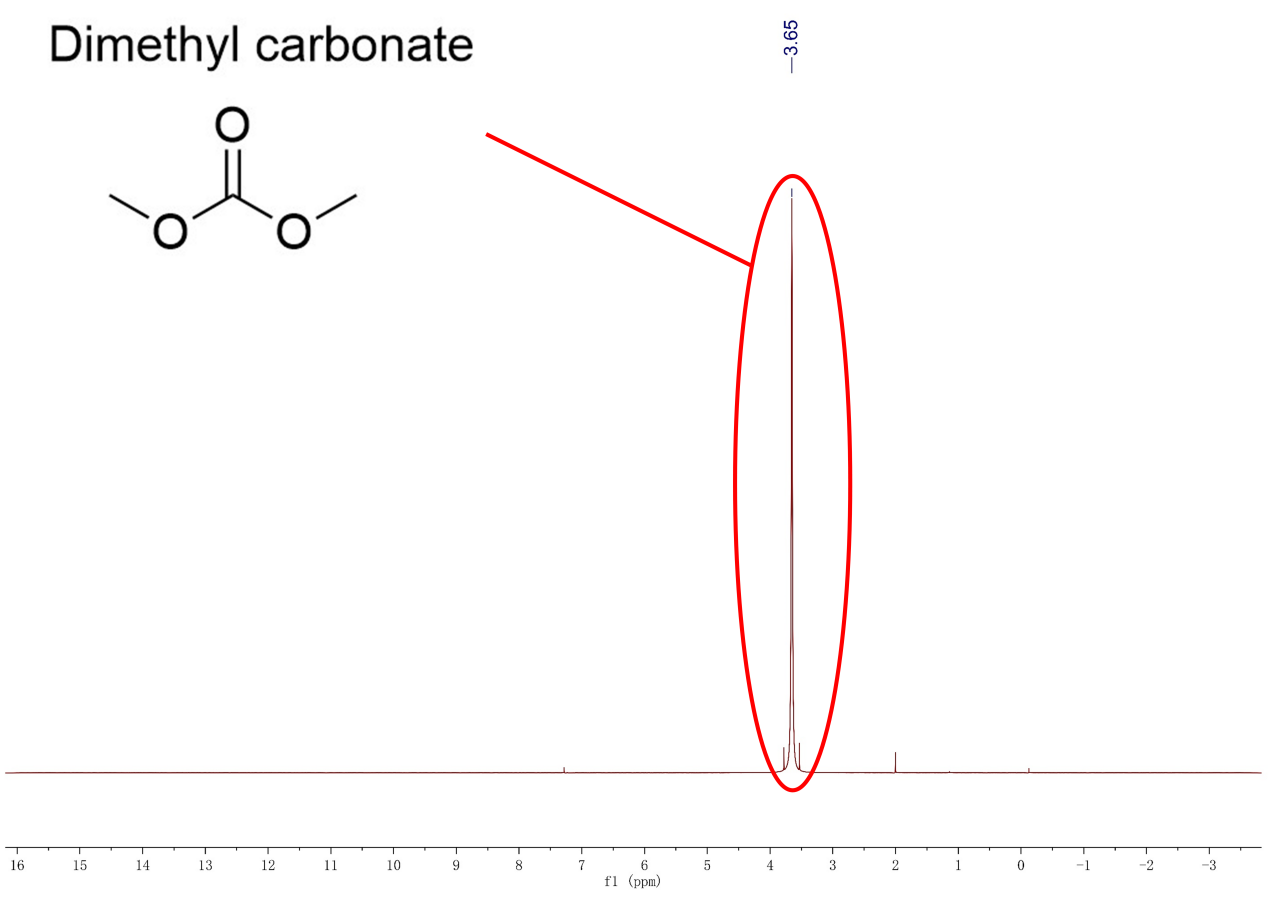
**

**Figure S34. ^1^H NMR of DMC.**


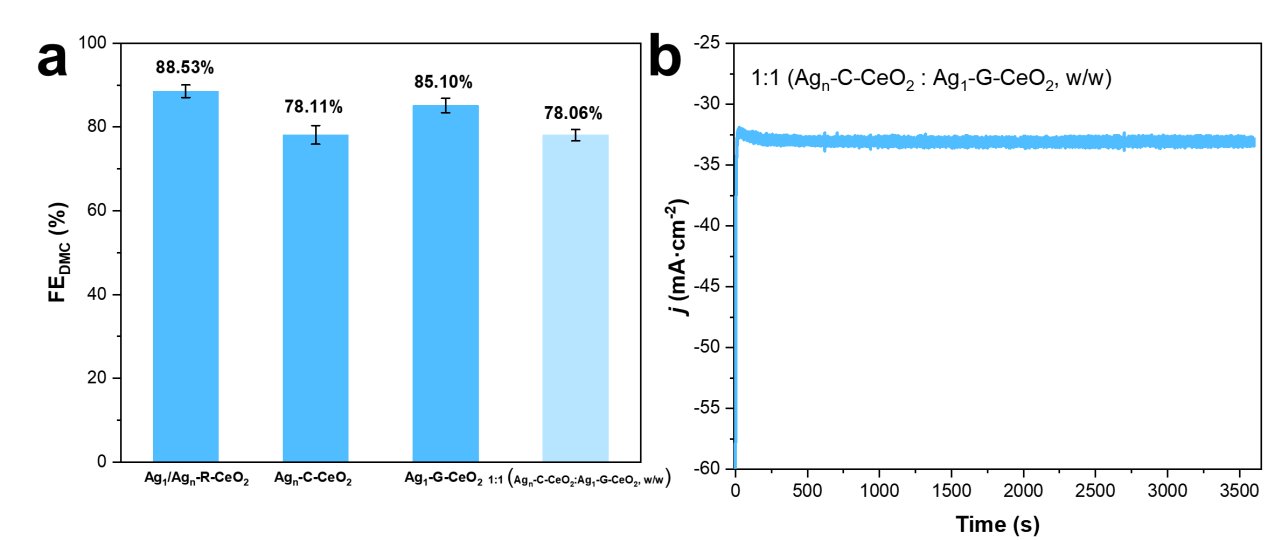


**Figure S35. a: FE_DMC_ of Ag_1_/Ag_n_-R-CeO_2_, Ag_n_-C-CeO_2_, Ag_1_-G-CeO_2_ and 1:1(Ag_n_-C-CeO_2_:Ag_1_-G-CeO_2_, w/w); b: Amperometric i-t curve of 1:1(Ag_n_-C-CeO_2_:Ag_1_-G-CeO_2_, w/w). Data for FE and product concentration represent the mean values of three independent measurements**. **Error bars indicate the standard deviation.**

**
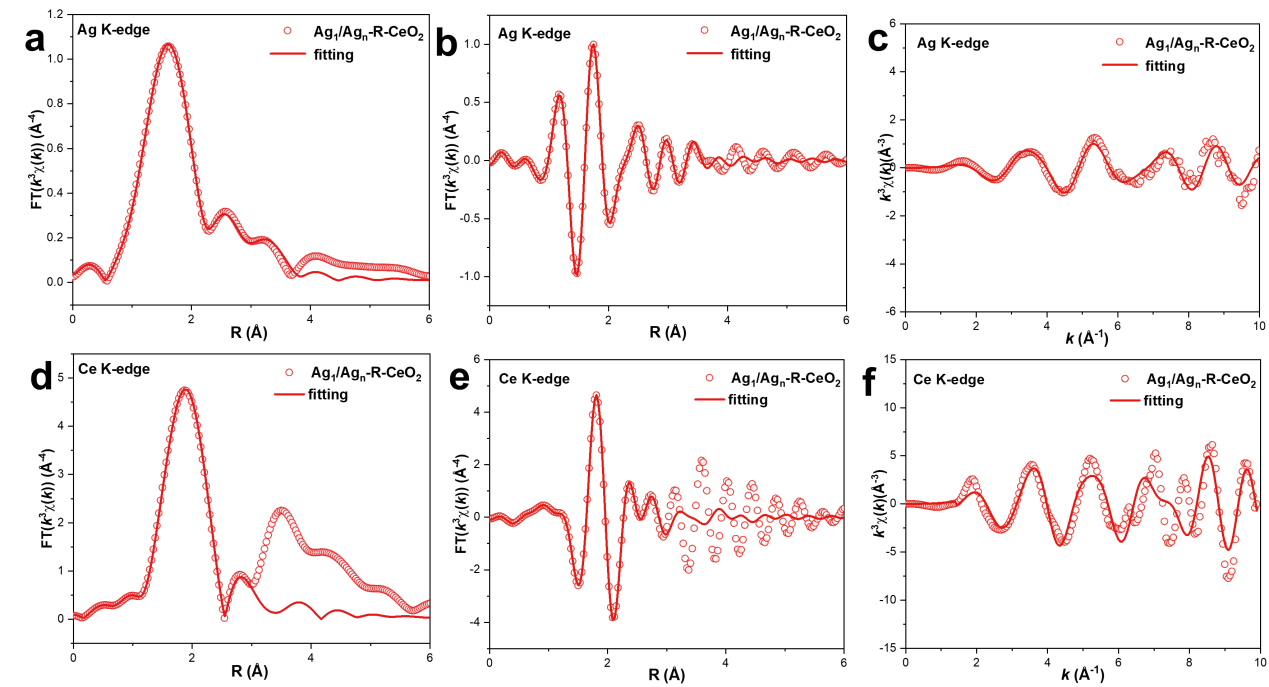
**

**Figure S36. Ag K-edge (a–c) and Ce K-edge (d–f) EXAFS Spectra of Post-Reaction Ag_1_/Ag_n_-R-CeO_2_: (a, d) R-space Curves, (b, e) Rmr (Radial Distribution Curves After Multiple Scattering Correction), and (c,f) k^3^-weighted χ(k) Curves (k-space).**

**
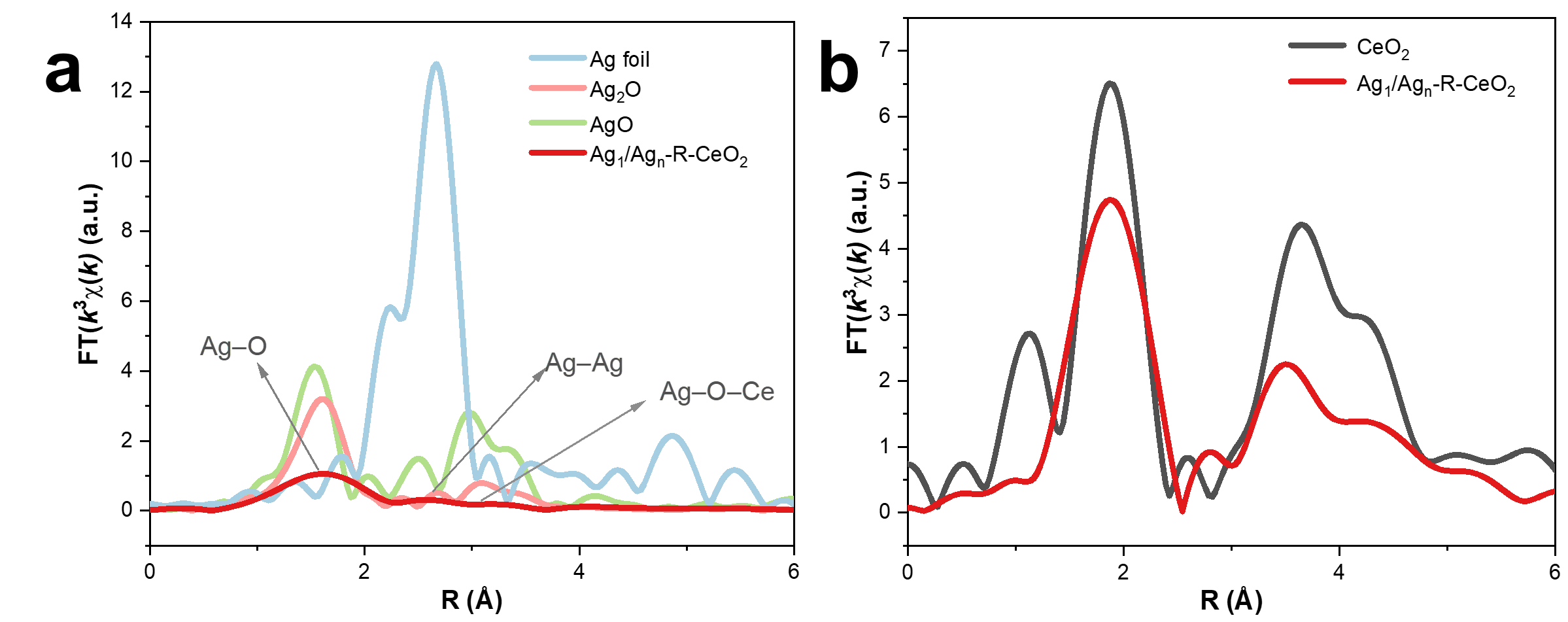
**

**Figure S37. Post-reaction Ag K-edge and Ce K-edge EXAFS Spectra Depicting the Local Coordination Environments of Ag and Ce.**

**Figure S38. Post-reaction Ce K-edge XANES Spectra.**

**
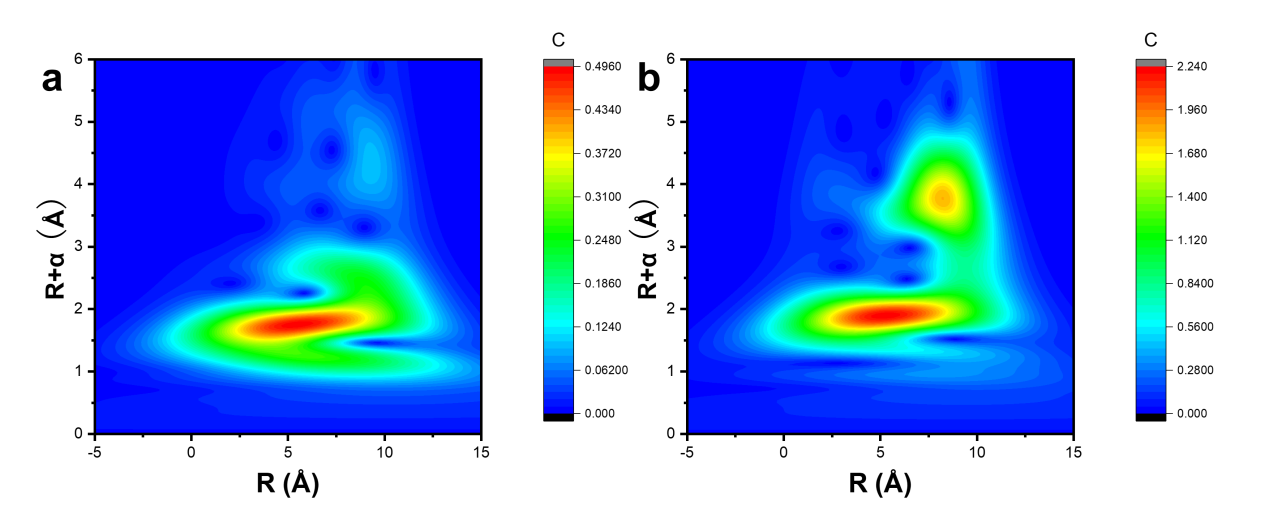
**

**Figure S39. Post-reaction WT-EXAFS spectra of Ag (a) and Ce (b) species (Ag_1_/Ag_n_-R-CeO_2_).**


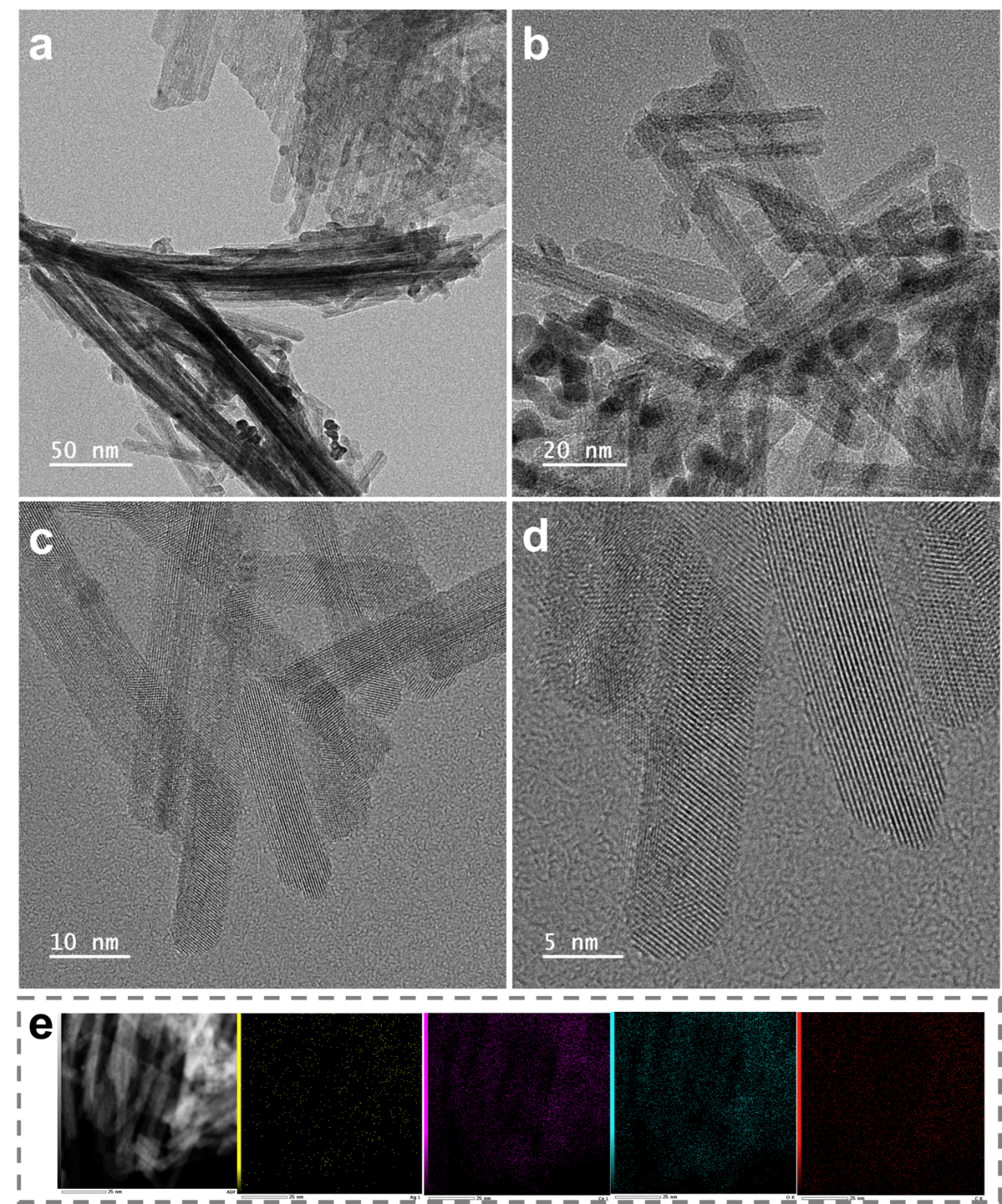


**Figure S40. a-d: TEM image of Ag_1_/Ag_n_-R-CeO_2_ after cycles of reaction; e: Mapping of Ag_1_/Ag_n_-R-CeO_2_ after 20 cycles of reaction.**

**
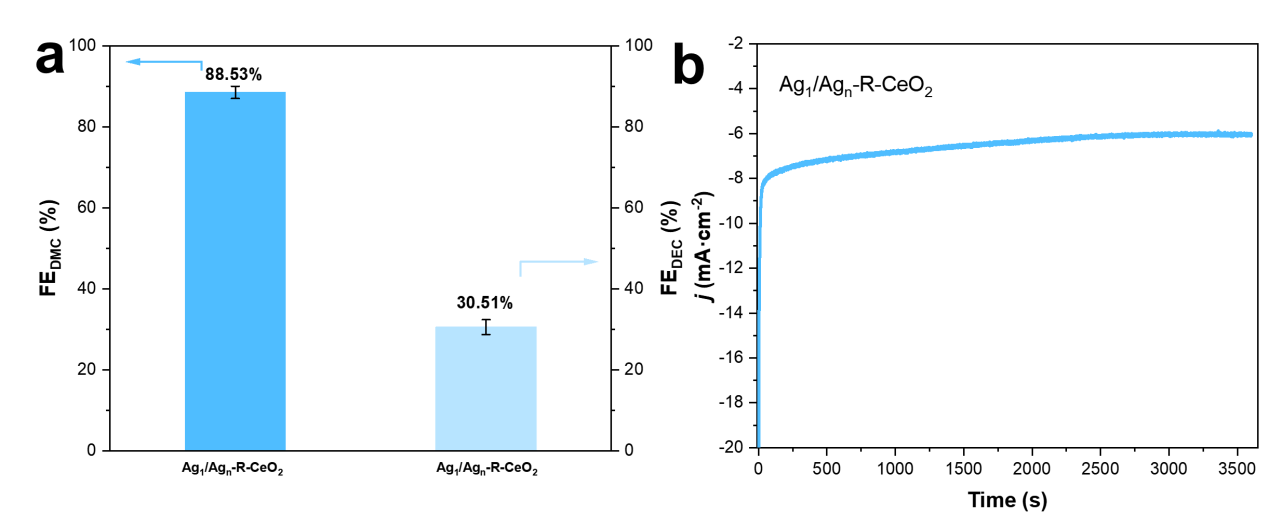
**

**Figure S41. a: FE_DMC_ of Ag_1_/Ag_n_-R-CeO_2_, Ag_n_-C-CeO_2_, Ag_1_-G-CeO_2_ and F_DEC_ of Ag_1_/Ag_n_-R-CeO_2_; b: Amperometric i-t curve of Ag_1_/Ag_n_-R-CeO_2_ for DEC. Data for FE and product concentration represent the mean values of three independent measurements**. **Error bars indicate the standard deviation.**


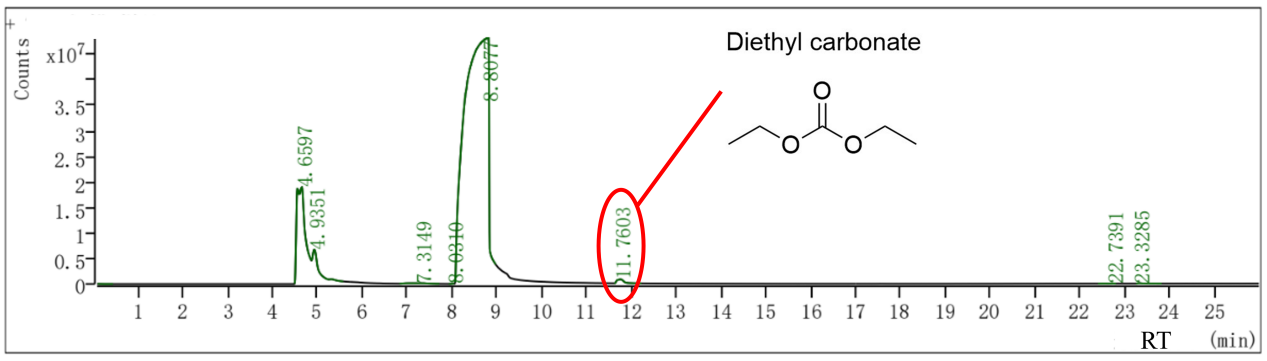


**Figure S42. GC-MS spectrum of DEC in post-reaction solution.**


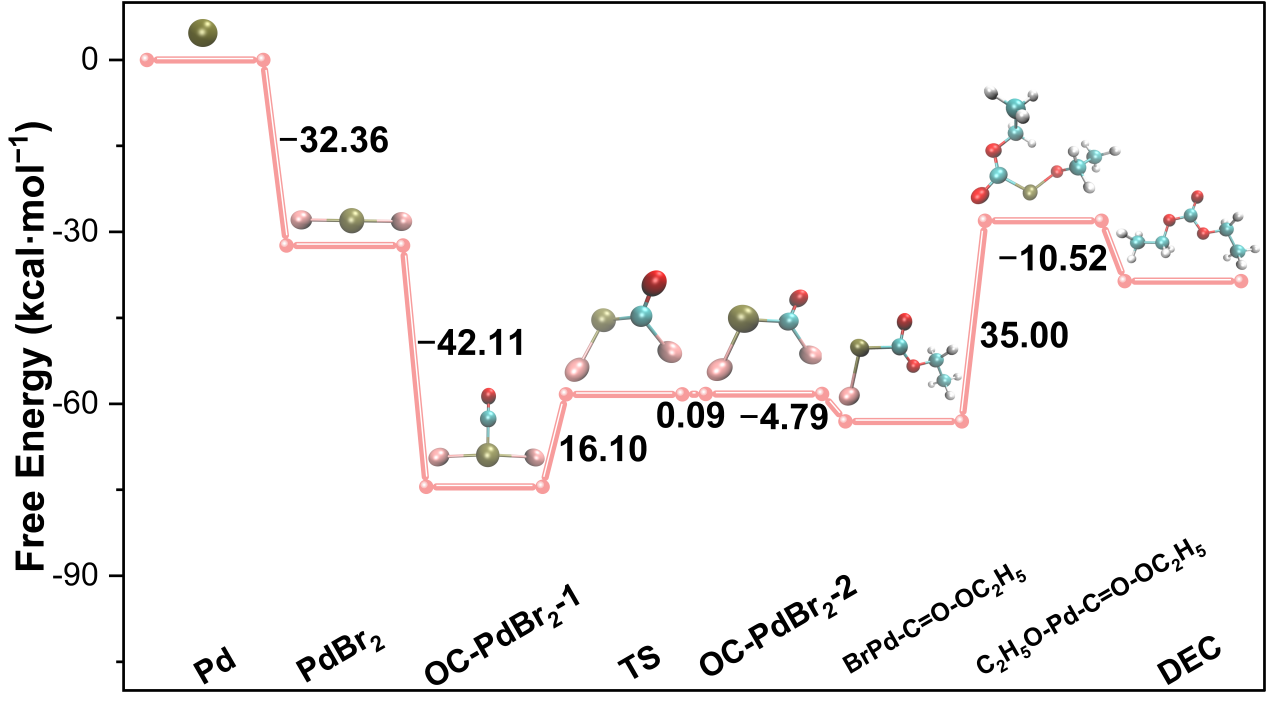


**Figure S43. DFT-calculated free energy profile for the Pd(CO)Br2-mediated catalytic cycle of DEC.**

**
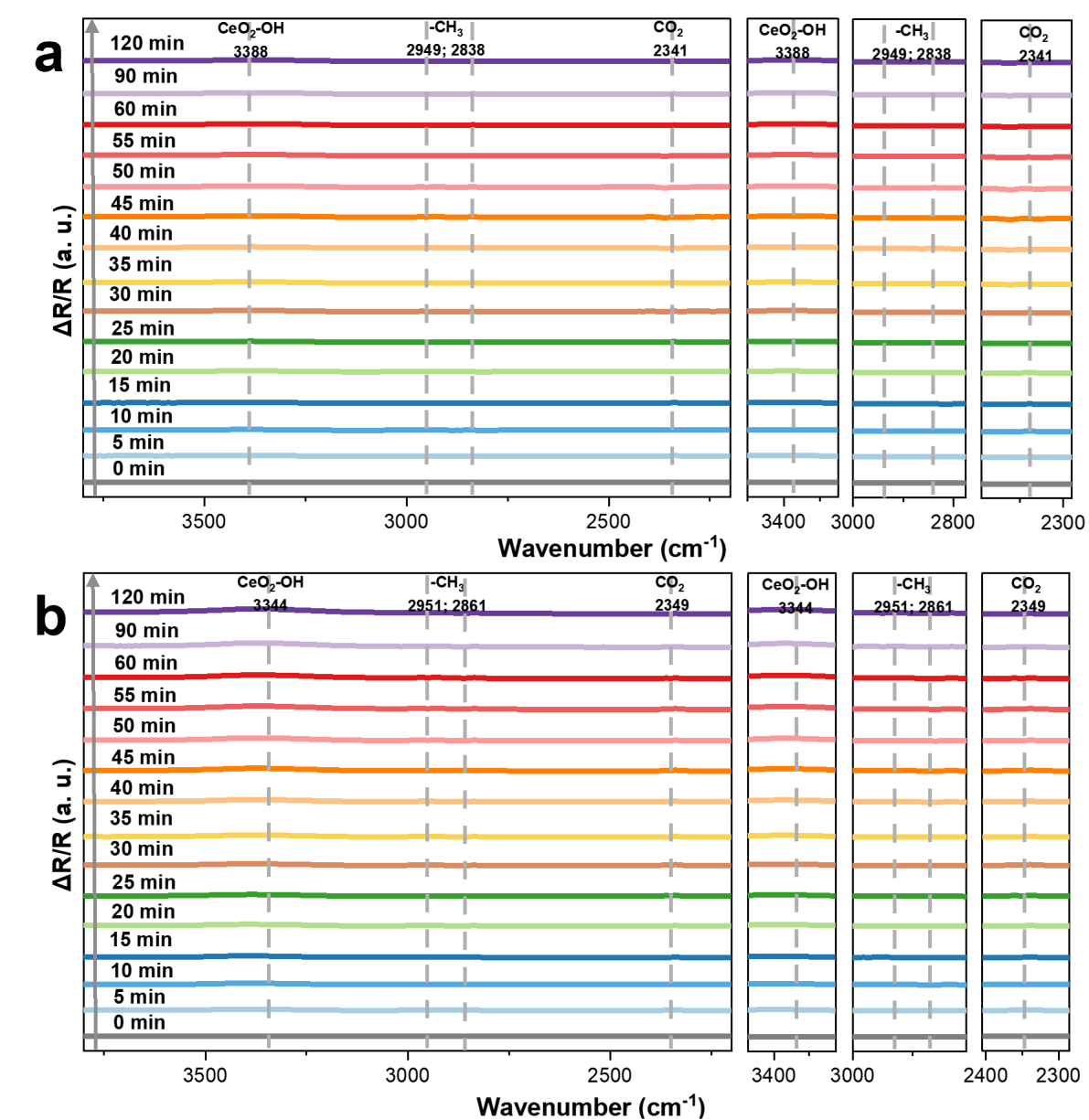
**

**Figure S44. a: *In situ* ATR–FTIR spectra of Ag_1_/Ag_n_-R-CeO_2_ under varying times; b: *In situ* ATR–FTIR spectra of R-CeO_2_ under varying times.**


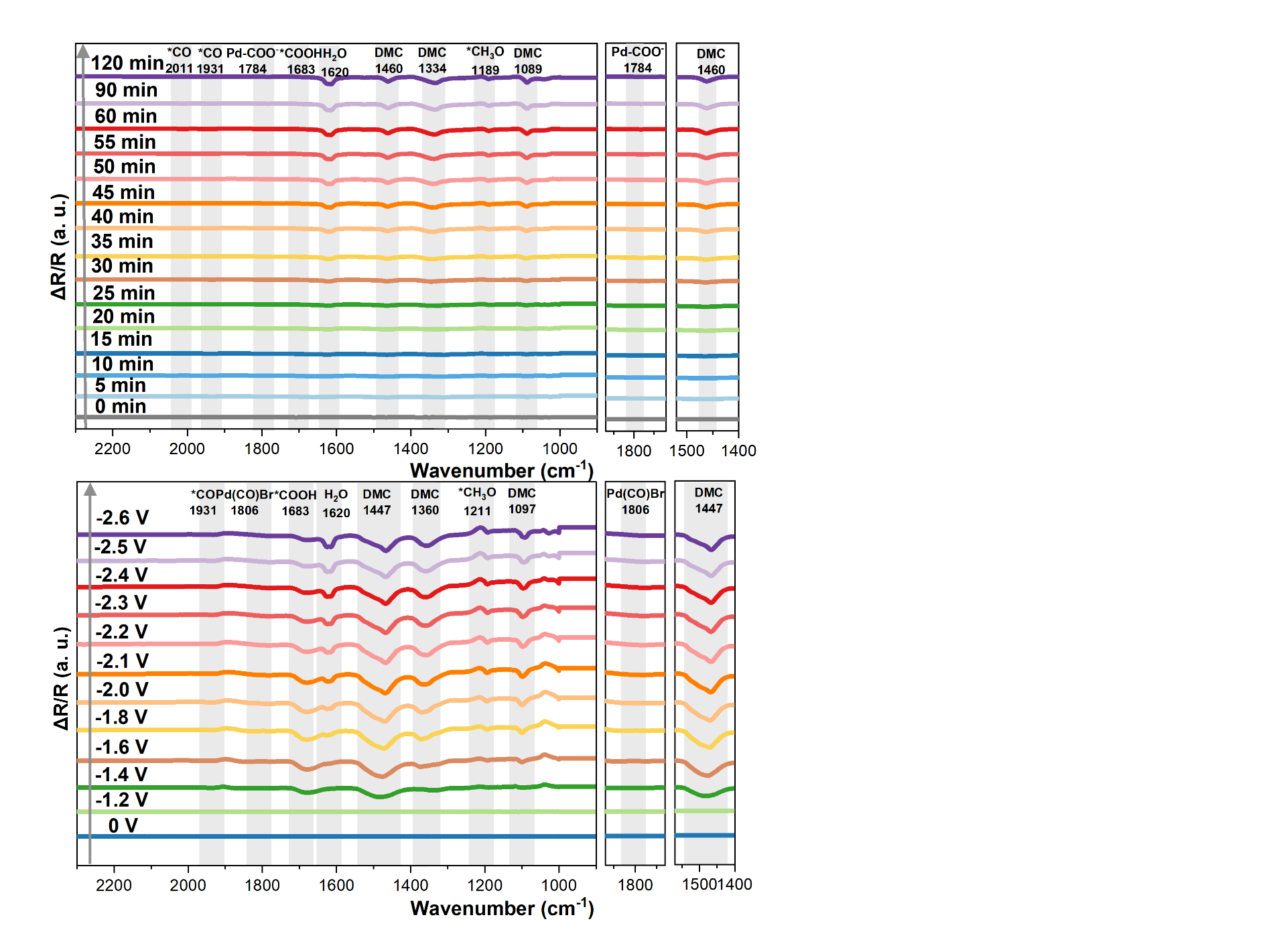


**Figure S45. a: *In situ* ATR–FTIR spectra of R-CeO_2_ under varying times;** **b:** ***In situ* ATR–FTIR spectra of R-CeO_2_ under varying voltages.**


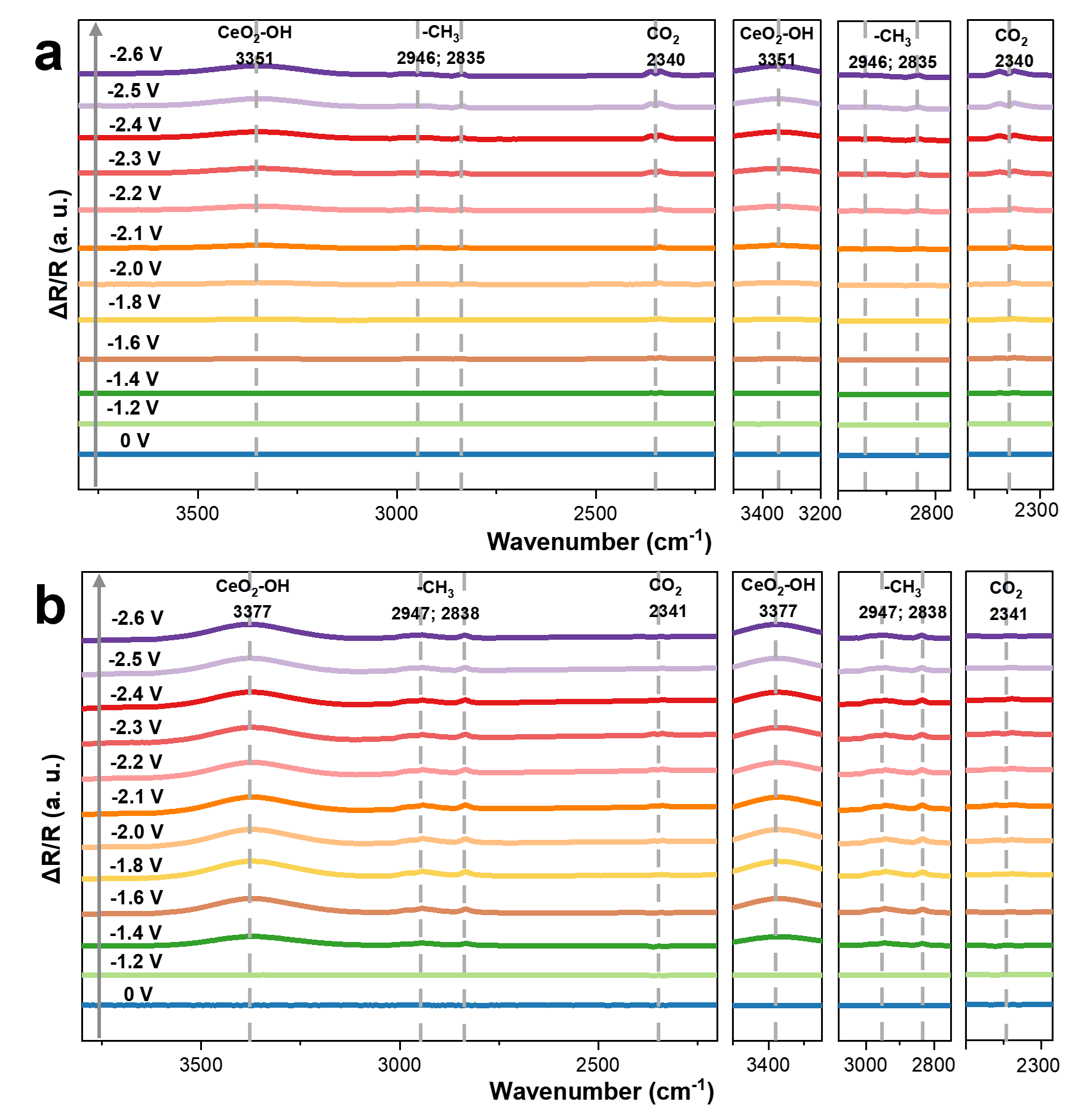


**Figure S46. a: *In situ* ATR–FTIR spectra of Ag_1_/Ag_n_-R-CeO_2_ under varying voltages; b: *In situ* ATR–FTIR spectra of R-CeO_2_ under varying voltages.**

**
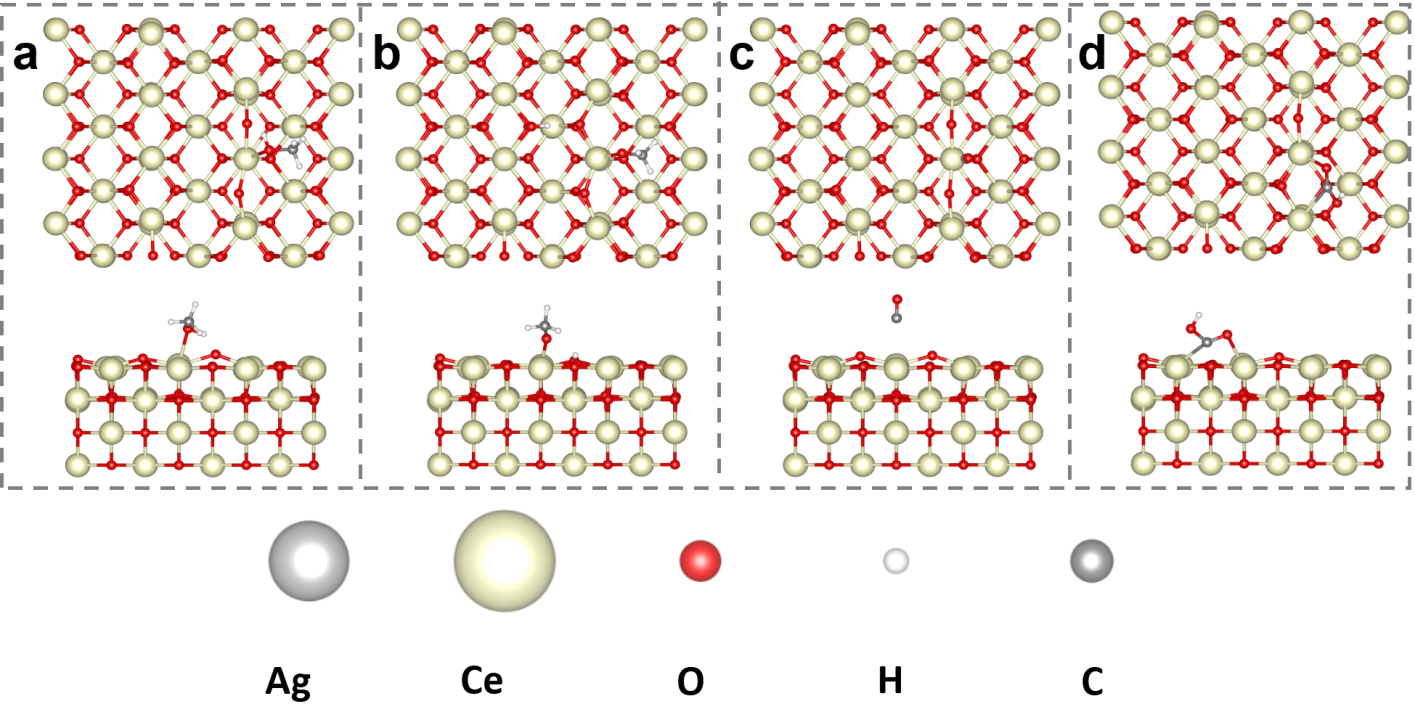
**

**Figure S47. DFT-Optimized Adsorption Configurations of Key Intermediates on the R-CeO_2_ Surface (a: with CH_3_OH; b: with CH_3_O-H; c: with *CO** **and d: with *COOH).**

**
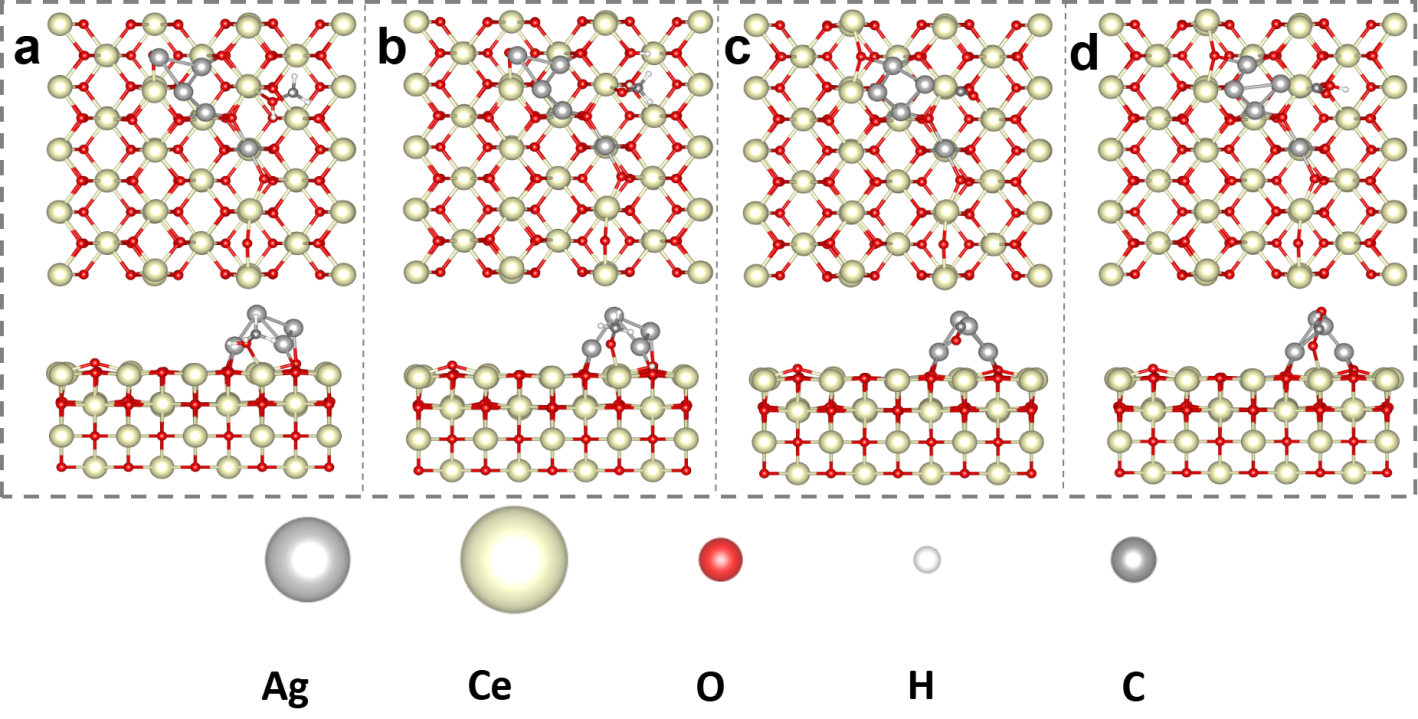
**

**Figure S48. DFT-Optimized Adsorption Configurations of Key Intermediates on the Ag_1_/Ag_n_-R-CeO_2_ Surface (a: with CH_3_OH; b: with CH_3_O-H; c: with *CO and d: with *COOH).**

**
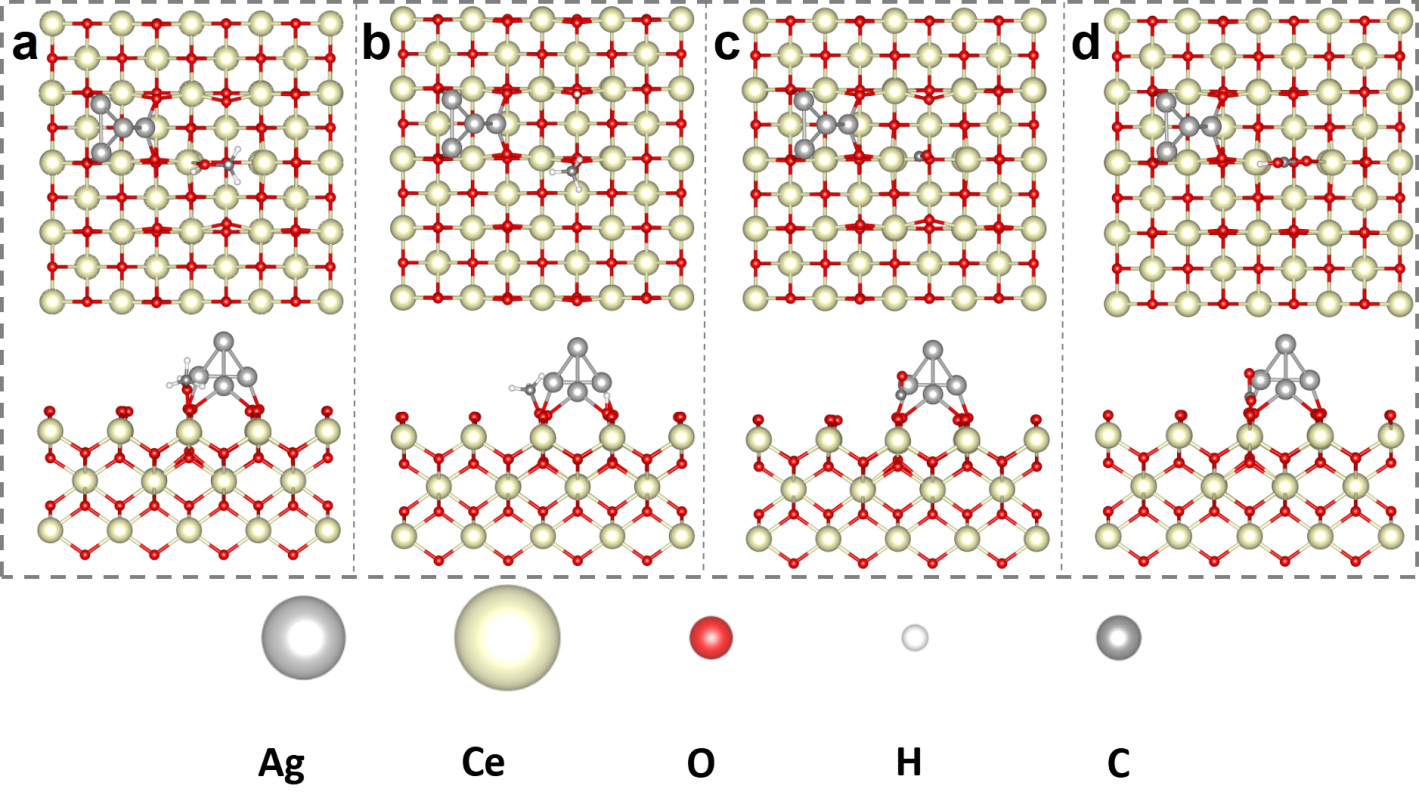
**

**Figure S49. DFT-Optimized Adsorption Configurations of Key Intermediates on the Ag_n_-C-CeO_2_ Surface (a: with CH_3_OH; b: with CH_3_O-H; c: with *CO and d: with *COOH).**

**
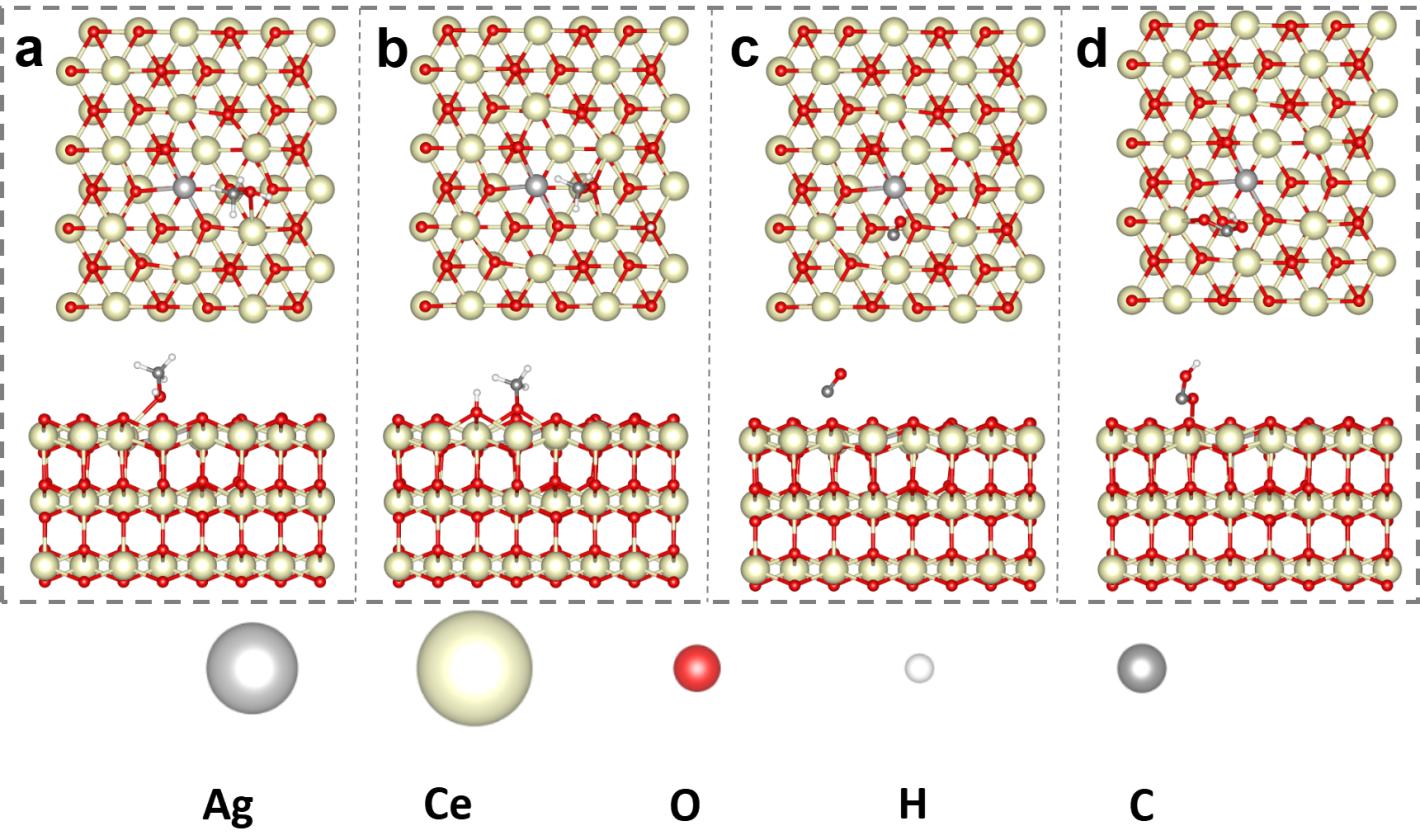
**

**Figure S50. DFT-Optimized Adsorption Configurations of Key Intermediates on the Ag_1_-G-CeO_2_ Surface (a: with CH_3_OH; b: with CH_3_O-H; c: with *CO and d: with *COOH).**


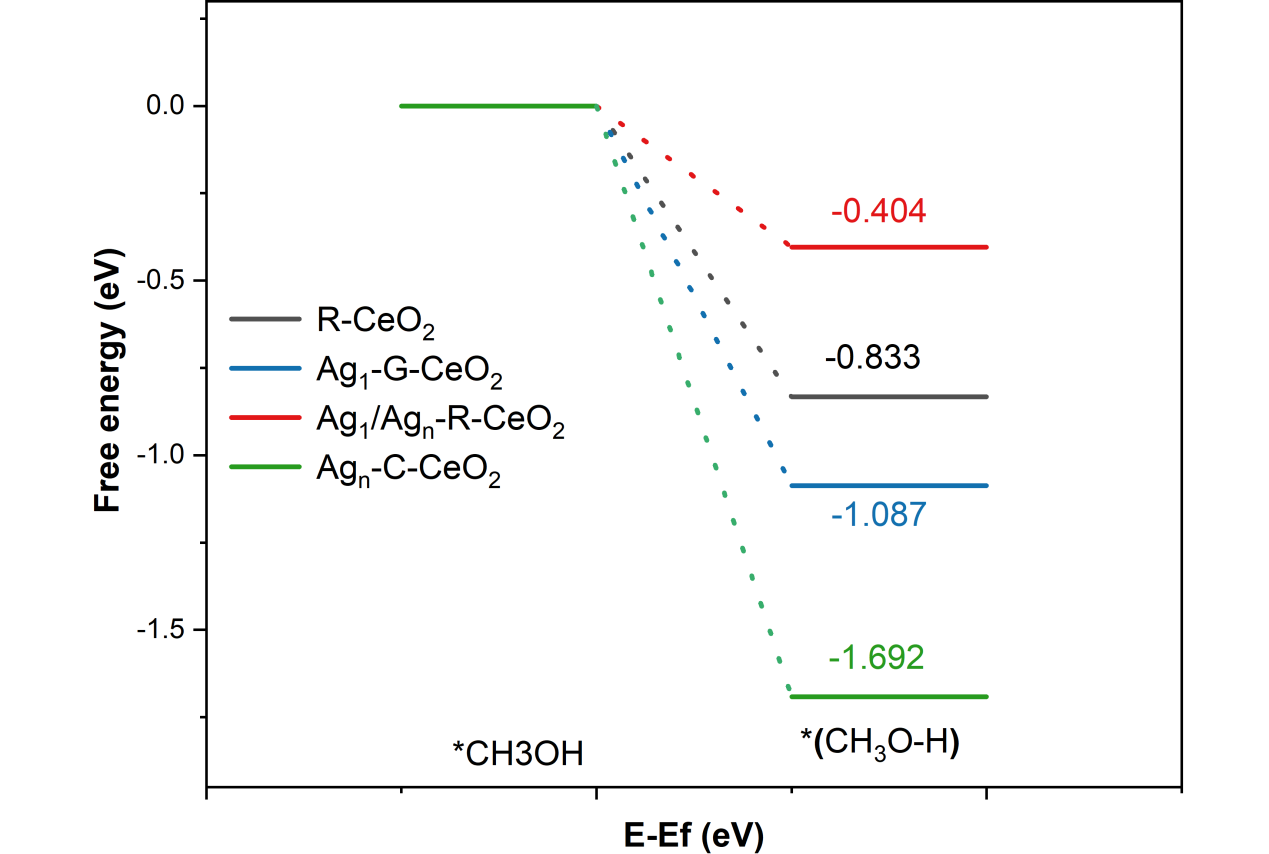


**Figure S51. CH_3_OH Decomposition Energy Barrier Diagram from DFT Calculations.**


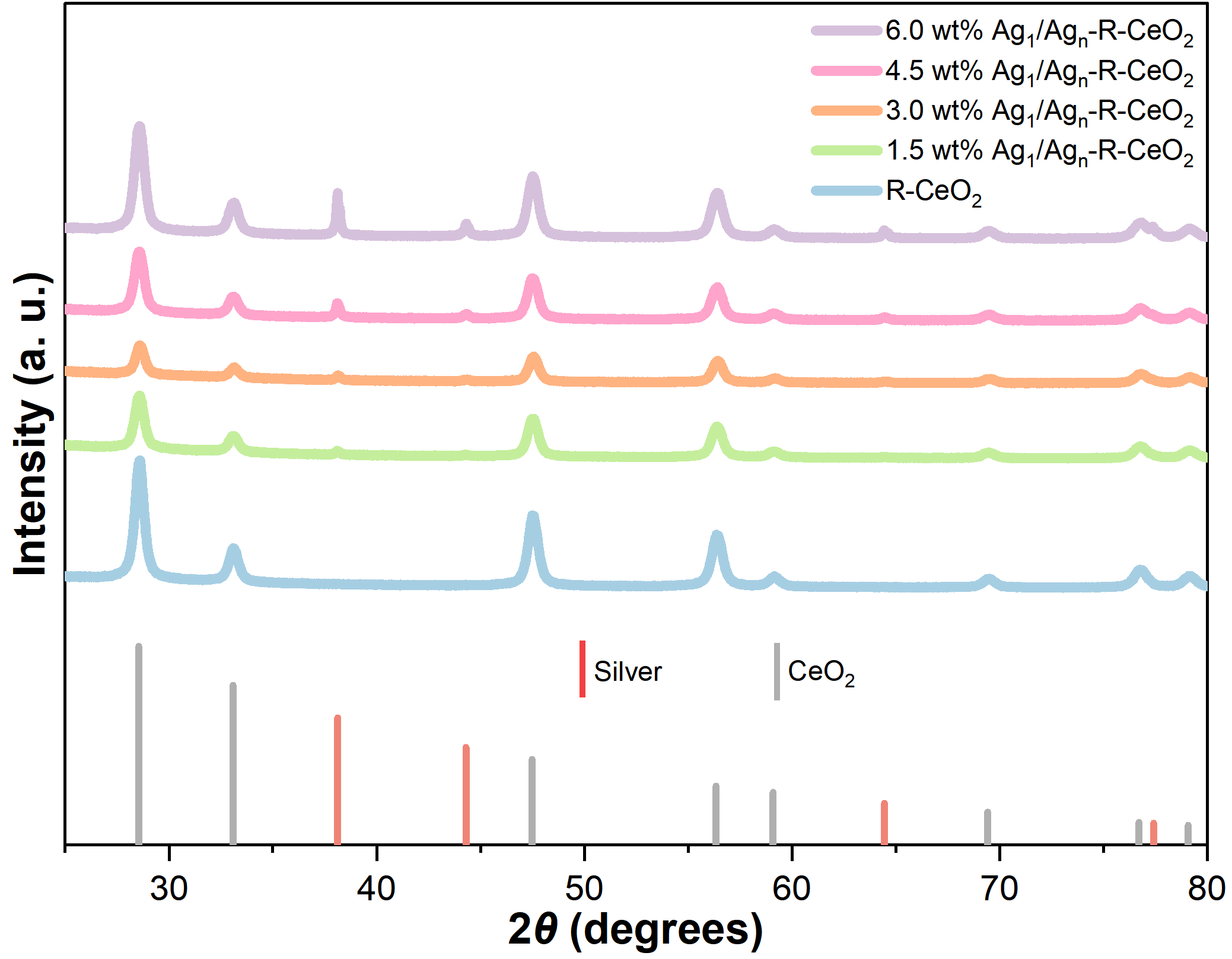


**Figure S52. XRD patterns of rod - shaped CeO_2_ (R-CeO_2_) and Ag_1_/Ag_n_-R-CeO_2_ with different Ag loadings (1.5-6.0 wt%).**


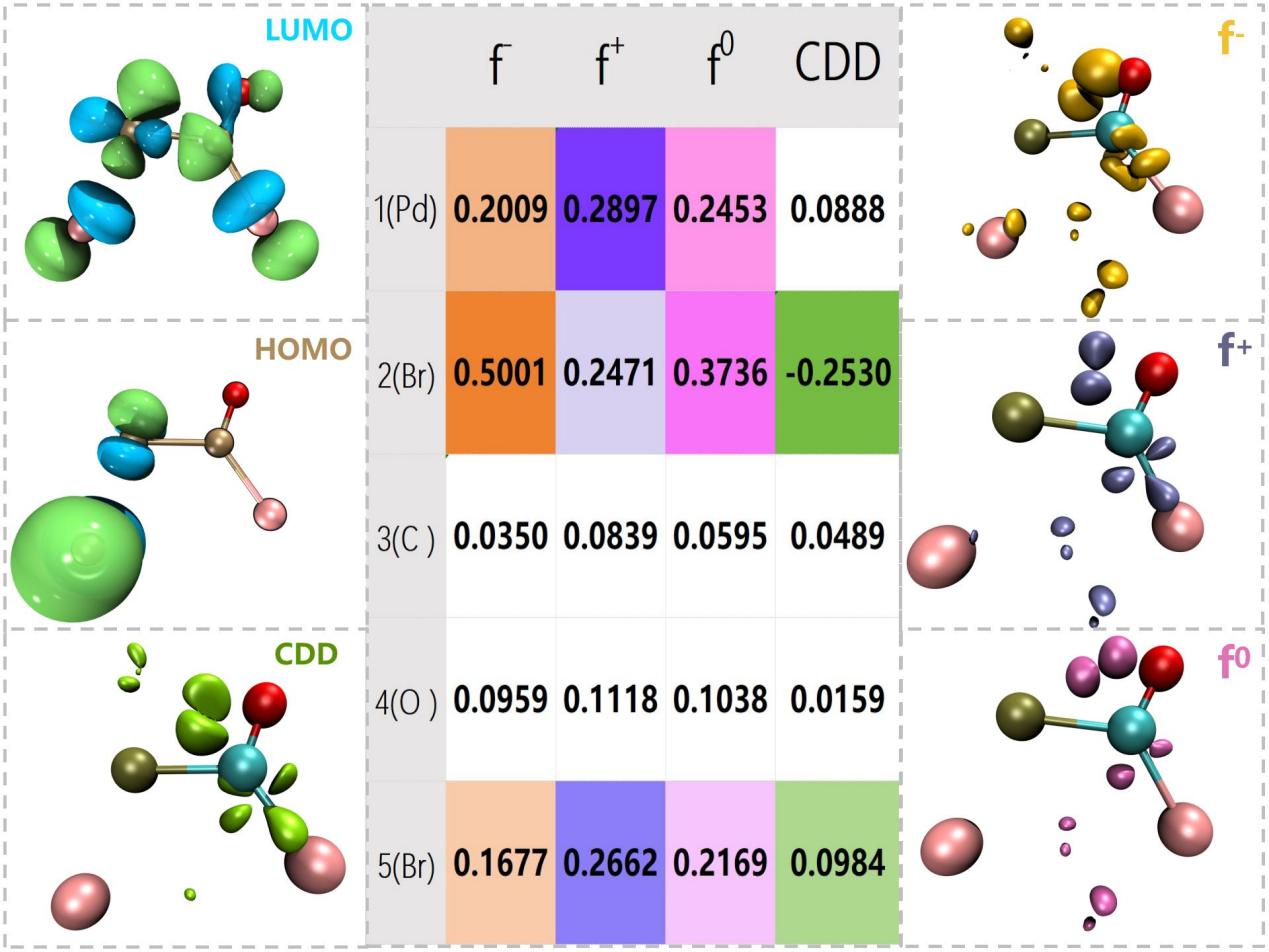


**Figure S53. The charge distribution and Fukui Index of Pd(CO)Br_2_.**


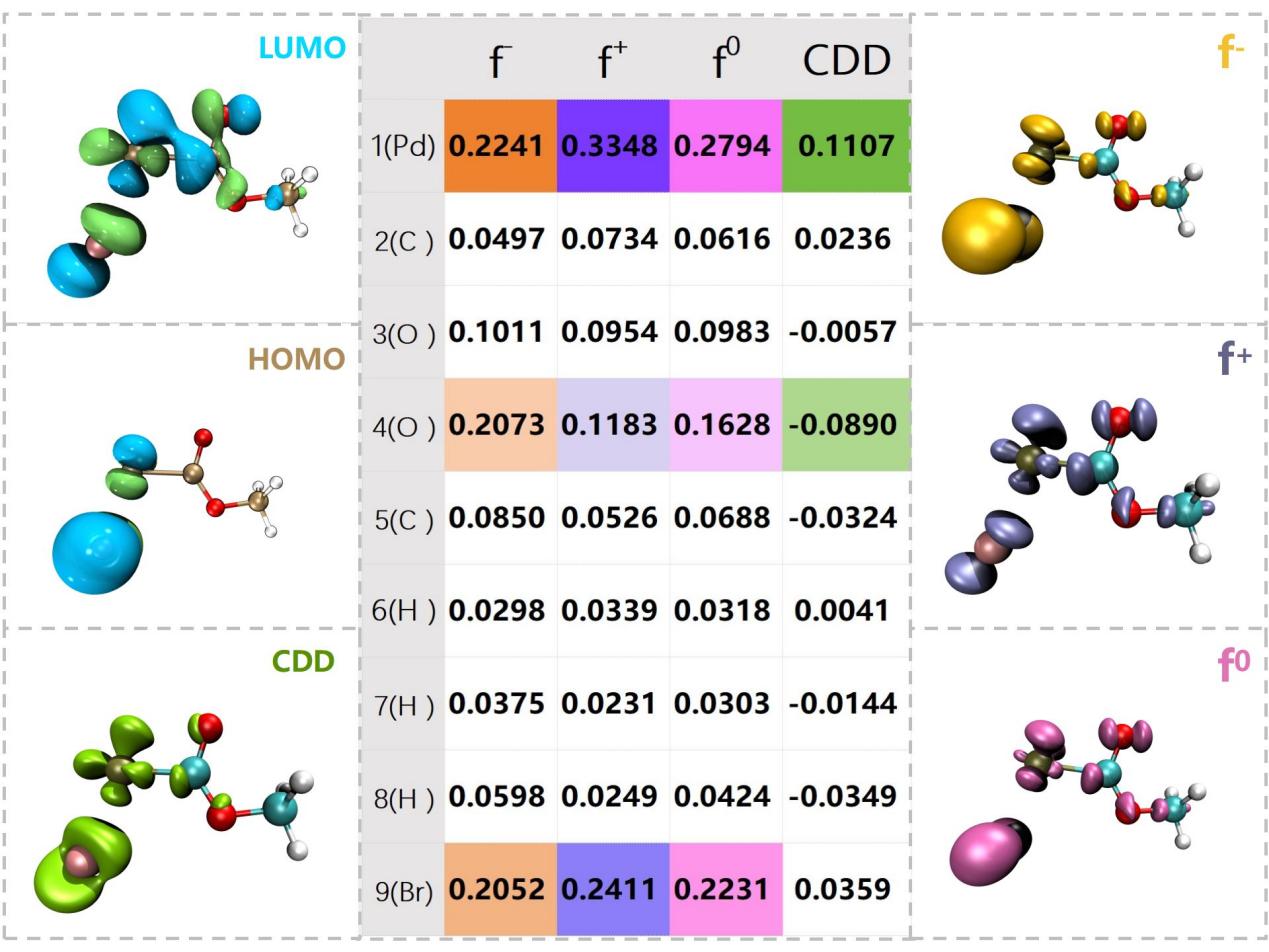


**Figure S54. The charge distribution and Fukui Index of CH_3_O–Pd(CO)Br.**

**Table S2. ICP of Ag_1_/Ag_n_-R-CeO_2_ Pre-reaction.**

| **Element** | **Wt%** |
| --- | --- |
| Ag | 3.04 |
| Ce | 78.53 |

**Table S3. Structural parameters from XRD Rietveld refinement of CeO_2_-based samples with different morphologies and Ag loadings.**

| **Sample** | **Phase** | ***a* (Å)** | ***b* (Å)** | ***c* (Å)** | ***V* (Å^3^)** | **Fraction** | **ε (%)** | **R_wp_** | **Chi2** |
| --- | --- | --- | --- | --- | --- | --- | --- | --- | --- |
| Ag_1_/Ag_n_-R-CeO_2_ | CeO_2_ | 5.42231 | 5.42231 | 5.42231 | 159.424 | 99.42 | 0.209 | 4.99 | 2.71 |
|  | Ag | 4.08596 | 4.08596 | 4.08596 | 68.216 | 0.58 | - |  |  |
| R-CeO_2_ | CeO_2_ | 5.43203 | 5.43203 | 5.43203 | 160.283 | 100 | 0.3887 | 4.39 | 1.88 |
| Ag_n_-C-CeO_2_ | CeO_2_ | 5.41427 | 5.41427 | 5.41427 | 158.716 | 95.97 | 0.0604 | 5.95 | 3.90 |
|  | Ag | 4.08625 | 4.08625 | 4.08625 | 68.230 | 4.03 | - |  |  |
| C-CeO_2_ | CeO_2_ | 5.41206 | 5.41206 | 5.41206 | 158.522 | 100 | 0.0196 | 6.46 | 4.94 |
| Ag_1_-G-CeO_2_ | CeO_2_ | 5.40997 | 5.40997 | 5.40997 | 158.338 | 95.85 | −0.019 | 6.01 | 2.87 |
|  | Ag | 4.08770 | 4.08770 | 4.08770 | 68.303 | 4.15 | - |  |  |
| G-CeO_2_ | CeO_2_ | 5.41010 | 5.41010 | 5.41010 | 158.349 | 100 | −0.017 | 4.91 | 2.89 |

$\text{ε}\text{=}\frac{\text{a}_{\text{strained}}\text{−}\text{a}_{\text{original}}}{\text{a}_{\text{original}}}\text{×100\%}$ (4)

$\text{ε}$: strain, %;

$\text{a}_{\text{strained}}$: strained dimension, Å;

$\text{a}_{\text{original}}$: original dimension, Å.

**Table S4. The fitted Rs, Rct and CPE of Ag-CeO_2_ with different morphologies and different Ag loadings (3.0 wt% or 0 wt%).**

| **Sample** | **3.0 wt%**  **Ag_1_/Ag_n_-R-CeO_2_** | **3.0 wt%**  **Ag_n_-C-CeO_2_** | **3.0 wt%**  **Ag_1_-G-CeO_2_** | **R-CeO_2_** | **C-CeO_2_** | **G-CeO_2_** |
| --- | --- | --- | --- | --- | --- | --- |
| **Rs (Ω)** | 8.613 | 6.586 | 5.374 | 7.997 | 4.676 | 7.997 |
| **Rct (Ω)** | 12.68 | 13.07 | 15.63 | 17.83 | 18.65 | 17.83 |
| **CPE** | 1.324×10^-7^ | 3.248×10^−8^ | 2.263×10^−8^ | 5.410×10^−8^ | 2.761×10^−8^ | 5.410×10^−8^ |

**Table S5. structural parameters obtained EXAFS spectroscopy pre-reaction.**

| **Sample** | - | **S_0_^2^** | **shell** | **CN*** | **R(Å)** | **σ^2^** | **ΔE_0_** | **R factor** |
| --- | --- | --- | --- | --- | --- | --- | --- | --- |
| - | Ag foil | 0.75 | Ag–Ag | 12 | 2.86 ± 0.01 | 0.0090 | 1.99 ± 0.33 | 0.0046 |
|  | CeO_2_ | 0.78 | Ce–O | 6 | 2.31 ± 0.01 | 0.0043 | 5.87 ± 0.43 | 0.0076 |
| **Ag_1_/Ag_n_-R-CeO_2_** | Ag | 0.75 | Ag–O | 3.2 | 2.09 ± 0.01 | 0.0220 | −9.40 ± 0.01 | 0.0006 |
|  |  | - | Ag–Ag | 1.4 | 2.88 ± 0.01 | 0.0130 | - | - |
|  |  | - | Ag–Ce | 1.2 | 3.19 ± 0.01 | 0.0101 | - | - |
|  | Ce | 0.78 | Ce–O | 5 | 2.28 ± 0.01 | 0.0050 | 2.88 ± 1.27 | 0.0039 |
|  |  | - | Ce–Ag | 1.3 | 3.19 ± 0.01 | 0.0150 | - | - |
| **Ag_n_-C-CeO_2_** | Ag | 0.75 | Ag–Ag | 3.9 | 2.83 ± 0.01 | 9.2 ± 0.8 | 9.70 ± 0.01 | 0.0044 |
|  |  | - | Ag–Ce | 0.8 | 3.30 ± 0.01 | 8.7 ± 0.1 | - | - |
|  | Ce | 0.78 | Ce–O | 7.8 | 2.25 ± 0.01 | 10.4 ± 0.6 | −0.74 ± 0.41 | 0.0045 |
|  |  | - | Ce–O–Ag | 3.4 | 3.10 ± 0.01 | 10.4 ± 2.0 | - | - |
|  |  | - | Ce–O–Ce | 8.6 | 3.76 ± 0.01 | 6.4 ± 0.8 | - | - |
| **Ag_1_-G-CeO_2_** | Ag | 0.75 | Ag–O | 3.8 | 2.25 ± 0.01 | 13.8 ± 2.4 | −9.80 ± 0.01 | 0.0059 |
|  |  | - | Ag–O–Ce | 0.9 | 3.29 ± 0.01 | 14.6 ± 0.1 | - | - |
|  | Ce | 0.78 | Ce–O | 7.6 | 2.25 ± 0.01 | 11.4 ± 0.6 | −0.22 ± 0.01 | 0.0040 |
|  |  | - | Ce–O–Ag | 2.3 | 3.07 ± 0.01 | 2.7 ± 1.5 | - | - |
|  |  | - | Ce–O–Ce | 9.7 | 3.75 ± 0.01 | 10.2 ± 1.2 | - | - |

CN: coordination numbers;

R: bond distance;

*σ*^2^: Debye-Waller factors;

Δ*E*_0_: the inner potential correction.

R factor: goodness of fit.

Error bounds that characterize the structural parameters obtained EXAFS spectroscopy were estimated as·CN+20%; R· ± ·1%; σ^2^ ± 20%.

**Table S6. Bader Charge Analysis of Ag_1_/Ag_n_-R-CeO_2_, Ag_n_-C-CeO_2_ and Ag_1_-G-CeO_2_.**

| **Ag_1_/Ag_n_-R-CeO_2_** | | | | **Ag_n_-C-CeO_2_** | | | | **Ag_1_-G-CeO_2_** | |
| --- | --- | --- | --- | --- | --- | --- | --- | --- | --- |
| Ce24 | −2.24 | Ag1 | −0.58 | Ce26 | −2.33 | Ag1 | −0.08 | Ce6 | −2.31 |
| Ce32 | −2.32 | Ag2 | 0.04 | Ce29 | −2.31 | Ag2 | −0.38 | Ce17 | −2.34 |
| Ce34 | −2.38 | Ag3 | −0.18 | Ce32 | −2.24 | Ag3 | −0.29 | Ce22 | −2.33 |
| Ce43 | −2.24 | Ag4 | 0.06 | Ce38 | −2.32 | Ag4 | −0.34 | Ce28 | −2.36 |
| Ce45 | −2.36 | Ag5 | −0.22 | Ce41 | −2.30 | - | - | Ce29 | −2.34 |
| Ce47 | −2.36 | - | - | Ce44 | −2.32 | - | - | Ce34 | −2.26 |
| - | - | - | - | - | - | - | - | Ag1 | −0.53 |

**Table S7. Calculated ICOHP and bond length parameters for Ce-O, Ag-O, and Ag-Ce interactions in CeO_2_-based Ag catalysts with different morphologies.**

| **Morphology** | **Ce-O ICOHP** | **Ce-O (Å)** | **Ag-O ICOHP** | **Ag-O (Å)** | **Ag-Ce ICOHP** | **Ag-Ce (Å)** |
| --- | --- | --- | --- | --- | --- | --- |
| **R-CeO_2_** | −4.91 | 2.231 | **-** | **-** | **-** | **-** |
| **C-CeO_2_** | −5.41 | 2.220 | **-** | **-** | **-** | **-** |
| **G-CeO_2_** | −3.88 | 2.340 | **-** | **-** | **-** | **-** |
| **Ag_1_/Ag_n_-R-CeO_2_** | −3.47 | 2.350 | −1.55 | 2.145 | −0.18 | 3.910 |
| **Ag_n_-C-CeO_2_** | −4.31 | 2.328 | −0.89 | 2.218 | −0.34 | 3.513 |
| **Ag_1_-G-CeO_2_** | −2.73 | 2.525 | −1.52 | 2.193 | −0.12 | 3.859 |

**Table S8. GC - MS test information of DMC in post - reaction Solution.**

| **Retention Time** | **Name** | **CAS#** | **Molecular Formula** | **Area** | **Match Score** | **Area%-T** | **Area%-M** |
| --- | --- | --- | --- | --- | --- | --- | --- |
| 5.1720 | Methyl formate | 107-31-3 | C_2_H_4_O_2_ | 13165589 | 97.4 | 0.76 | 0.96 |
| 7.7445 | Methyl Alcohol | 67-56-1 | CH_4_O | 1367137042 | 96.4 | 78.91 | 100 |
| 8.9619 | Dimethyl Carbonate | 616-38-6 | C_3_H_6_O_3_ | 50948125 | 98.0 | 2.94 | 3.73 |
| 9.9590 | Toluene | 108-88-3 | C_7_H_8_ | 3231564 | 98.3 | 0.19 | 0.24 |

**Table S9. ICP of Ag_1_/Ag_n_-R-CeO_2_ after 20 cycles of reaction.**

| **Element** | **Wt%** |
| --- | --- |
| Ag | 2.26 |
| Ce | 56.48 |

**Table S10. structural parameters obtained EXAFS spectroscopy post-reaction.**

| **Sample** | - | **S_0_^2^** | **shell** | **CN*** | **R(Å)** | **σ^2^** | **ΔE_0_** | **R factor** |
| --- | --- | --- | --- | --- | --- | --- | --- | --- |
| **Ag_1_/Ag_n_-R-CeO_2_** | Ag | 0.75 | Ag–O | 3.3 | 2.28 ± 0.01 | 0.0131 | −6.91 ± 0.01 | 0.0012 |
|  |  | - | Ag–Ag | 1.3 | 2.91 ± 0.01 | 0.0146 | - | - |
|  |  | - | Ag–Ce | 1.1 | 3.25 ± 0.01 | 0.0146 | - | - |
|  | Ce | 0.78 | Ce–O | 4.9 | 2.34 ± 0.01 | 0.0033 | 8.18 ± 0.30 | 0.0015 |
|  |  | - | Ce–Ag | 1.3 | 3.25 ± 0.01 | 0.0099 | - | - |

**Table S11. GC - MS test information of DEC in post - reaction Solution.**

| **Retention Time** | **Name** | **CAS#** | **Molecular Formula** | **Area** | **Match Score** | **Area%-T** | **Area%-M** |
| --- | --- | --- | --- | --- | --- | --- | --- |
| 11.7603 | Diethyl carbonate | 105-58-8 | C_5_H_10_O_3_ | 9094158 | 98.7 | 0.42 | 0.51 |

**Table S12. Performance comparison of convergent paired electrosynthesis from CO_2_ to DMC.**

| **Catalyst** | **Current Density**  **(mA·cm^-2^)** | **FE_DMC_/Yield** | **Cyclic Test**  **(n)** | **Long-term**  **Reaction**  **(h)** | **Ref** |
| --- | --- | --- | --- | --- | --- |
| **Ag_1_/Ag_n_-R-CeO_2_**  (Electrosynthesis from CO_2_) | **52.5** | **88.53%** | **20** | **-** | **This work** |
| Ni SAs/OMMNC^[1]^  (Electrosynthesis from CO_2_) | 12 | 80% | - | 17 | *EES.*2023,16,502 |
| Co-CPY/CNTs、  Co-TAA/CNTs、  Co-PHE/CNTs^[3]^  (Electrosynthesis from CO_2_) | 16 | 96 μmol cm^−^² h^−^¹ | - | 12 | *CEJ*.2024,486 |
| Cathode: Au electrodes  Anode: Glassy carbon^[2]^  (Electrosynthesis from CO_2_) | 12 | 60% | - | - | *Nat.Energy.*2021, 6,733 |
| Pd-B^[4]^  (Electrosynthesis from CO) | 30 | 43% | - | 3 | *CEJ.*2024,498 |
| Pd-B (iii)^[5]^  (Electrosynthesis from CO) | 4 | 83% | - | - | *Nat.Commun*.2019,10,4807 |
| Pd/VGCF^[6]^  (Electrosynthesis from CO) | 12 | 67% | 3 | - | *J.Catal.* 2004,221,110 |
| Copper carbonyl^[7]^  (Electrosynthesis from CO) | 3 | 6% | - | - | *ACS.Catal.*2018,9,859 |
| Au/carbon^[8]^  (Electrosynthesis from CO) | 11 | 35% | 5 | 4 | *J.Phys.Chem.B* 2005,109,9140 |
| HAuCl_4_/AC^[9]^  (Electrosynthesis from CO) | 2.4 | 5.1% | - | - | *J.Am.Chem.Sco*. 2004,126,5346 |

**Table S13. ICP of the concentration of Br**^−^ **Pre-reaction and after 2 hours of reaction**

| **Element** | **Pre-reaction**  **mol/L** | **Post-reaction**  **mol/L** |
| --- | --- | --- |
| **Br**^−^ | 0.2 | 0.0087 |

1. **The DFT calculation**

**3.1 The DFT calculation of VASP**

We have employed the Vienna Ab Initio Package (VASP) [10, 11] to perform all spin-polarized density functional theory (DFT) calculations within the generalized gradient approximation (GGA) using the Perdew-Burke-Ernzerhof (PBE) [12] formulation. We have chosen the projected augmented wave (PAW) potentials [13, 14] to describe the ionic cores and take valence electrons into account using a plane wave basis set with a kinetic energy cutoff of 450 eV. Partial occupancies of the Kohn−Sham orbitals were allowed using the Gaussian smearing method and a width of 0.05 eV. The electronic energy was considered self-consistent when the energy change was smaller than 10^−5^ eV. A geometry optimization was considered convergent when the energy change was smaller than 0.02 eV Å^−1^. The vacuum spacing in a direction perpendicular to the plane of the structure is 18 Å. The weak interaction was described by DFT+D3 method using empirical correction in Grimme’s scheme [15]. To consider the strong correlation effects of transition metal in structure, all calculations were carried out by using the spin-dependent GGA plus Hubbard correction U method [16, 17], and the effective Ueff parameters was 4.0 eV Ce in CeO_2_.

The Gibbs free energy change (ΔG) of each chemical reaction was calculated by Eq. 4,

$\text{∆}\text{G}\text{=∆}\text{E}\text{+∆}\text{ZPE}\text{−}\text{T}\text{∆}\text{S}$ (5)

where E is the calculated total energy, ZPE is the zero-point energy, T is the temperature, and S is the entropy.

**3.2 Computational Details of Gaussian**

The free energy diagram of the reactions was calculated using ORCA (version 5.0.4) software package [18]. ORCA is a versatile quantum chemistry program capable of performing various types of calculations, including density functional theory (DFT), Hartree-Fock (HF), and post-HF methods. Geometry optimizations and frequency calculations were carried out using the B3LYP/Def2-TZVP basis set. To account for dispersion interactions, the DFT-D3 correlation correction was included in the calculations [15]. This correction accounts for the long-range dispersion forces that are not adequately captured by standard DFT functionals. The D3 correction was applied with the Becke-Johnson damping scheme. Thermal corrections were performed in Shermo code [19].

**References**

[1] X. Li, S.-G. Han, W. Wu, K. Zhang, B. Chen, S.-H. Zhou, D.-D. Ma, W. Wei, X.-T. Wu, R. Zou, Q.-L. Zhu, Convergent paired electrosynthesis of dimethyl carbonate from carbon dioxide enabled by designing the superstructure of axial oxygen coordinated nickel single-atom catalysts, Energy & Environmental Science, 16 (2023) 502-512.

[2] K.M. Lee, J.H. Jang, M. Balamurugan, J.E. Kim, Y.I. Jo, K.T. Nam, Redox-neutral electrochemical conversion of CO_2_ to dimethyl carbonate, Nature Energy, 6 (2021) 733-741.

[3] S.-G. Han, S. Zhou, X. Li, J. Zhao, W.-B. Wei, L. Zheng, D.-D. Ma, X.-T. Wu, Q.-L. Zhu, Structure-induced interfacial activation convoying the CO-relayed conversion of CO_2_ to dimethyl carbonate, Chemical Engineering Journal, 486 (2024).

[4] K.-A. Wang, Z.-L. Wang, H.-C. Hu, H.-B. Zhu, H. Yang, Single B-doped palladium electrocatalyst Enabling direct cathodic coupling of carbon dioxide with methanol into dimethyl carbonate, Chemical Engineering Journal, 498 (2024).

[5] T.-T. Zhuang, D.-H. Nam, Z. Wang, H.-H. Li, C.M. Gabardo, Y. Li, Z.-Q. Liang, J. Li, X.-J. Liu, B. Chen, W.R. Leow, R. Wu, X. Wang, F. Li, Y. Lum, J. Wicks, C.P. O’Brien, T. Peng, A.H. Ip, T.-K. Sham, S.-H. Yu, D. Sinton, E.H. Sargent, Dopant-tuned stabilization of intermediates promotes electrosynthesis of valuable C_3_ products, Nature Communications, 10 (2019).

[6] I. Yamanaka, Electrocatalytic synthesis of DMC over the Pd/VGCF membrane anode by gas–liquid–solid phase-boundary electrolysis, Journal of Catalysis, 221 (2004) 110-118.

[7] B.J.V. Davies, M. Šarić, M.C. Figueiredo, N.C. Schjødt, S. Dahl, P.G. Moses, M. Escudero-Escribano, M. Arenz, J. Rossmeisl, Electrochemically Generated Copper Carbonyl for Selective Dimethyl Carbonate Synthesis, ACS Catalysis, 9 (2018) 859-866.

[8] A. Funakawa, I. Yamanaka, K. Otsuka, Active Control of Methanol Carbonylation Selectivity over Au/Carbon Anode by Electrochemical Potential, *J. Phys. Chem. B* 2005, 109, 9140-9147.

[9] A. Funakawa, I. Yamanaka, S. Takenaka, K. Otsuka, Selectivity Control of Carbonylation of Methanol to Dimethyl Oxalate and Dimethyl Carbonate over Gold Anode by Electrochemical Potential, JACS, 2004.

[10] G. Kresse, J.F.J.C.M. Science, Efficiency of ab-initio total energy calculations for metals and semiconductors using a plane-wave basis set, 6 (1996) 15-50.

[11] G.G. Kresse, C.m. J.J. Furthmüller %J Physical review. B, Efficient Iterative Schemes for Ab Initio Total-Energy Calculations Using a Plane-Wave Basis Set, 54 (1996) 11169.

[12] J.P. Perdew, K. Burke, M.J.P.R.L. Ernzerhof, Generalized Gradient Approximation Made Simple, 77 (1998) 3865-3868.

[13] G. Kresse, D.J.P.R.B. Joubert, From ultrasoft pseudopotentials to the projector augmented-wave method, 59 (1999) 1758-1775.

[14] P.E.J.P.R.B.C.M. Blochl, Projector augmented-wave method, 50 (1994) 17953-17979.

[15] S. Grimme, J. Antony, S. Ehrlich, H.J.J.o.C.P. Krieg, A consistent and accurate ab initio parametrization of density functional dispersion correction (DFT-D) for the 94 elements H-Pu, 132 (2010) 154104.

[16] V.I. Anisimov, J. Zaanen, O.K.J.P.r.B. Andersen, Condensed matter, Band Theory and Mott Insulators: Hubbard U Instead of Stoner I, 44 (1991) 943-954.

[17] Anisimov V. I., Aryasetiawan F., Lichtenstein A. I. First-principles calculations of the electronic structure and spectra of strongly correlated systems: the LDA + U method[J]. Journal of Physics: Condensed Matter, 1997, 9(4):767-808.

[18] F.J.W.I.R.C.M.S. Neese, Software update: The ORCA program system—Version 5.0, 12 (2022).

[19] T. Lu, Q.J.C. Chen, T. Chemistry, Shermo: A general code for calculating molecular thermochemistry properties, (2021) 113249.
